# Antibody to HSV gD peptide induced by vaccination does not protect against HSV-2 infection in HSV-2 seronegative women

**DOI:** 10.1371/journal.pone.0176428

**Published:** 2017-05-11

**Authors:** Peter B. Gilbert, Jean-Louis Excler, Georgia D. Tomaras, Lindsay N. Carpp, Barton F. Haynes, Hua-Xin Liao, David C. Montefiori, Supachai Rerks-Ngarm, Punnee Pitisuttithum, Sorachai Nitayaphan, Jaranit Kaewkungwal, Gustavo H. Kijak, Sodsai Tovanabutra, Donald P. Francis, Carter Lee, Faruk Sinangil, Phillip W. Berman, Nakorn Premsri, Prayura Kunasol, Robert J. O’Connell, Nelson L. Michael, Merlin L. Robb, Rhoda Morrow, Lawrence Corey, Jerome H. Kim

**Affiliations:** 1 Statistical Center for HIV/AIDS Research and Prevention, Vaccine and Infectious Disease Division, Fred Hutchinson Cancer Research Center, Seattle, Washington, United States of America; 2 Department of Biostatistics, University of Washington, Seattle, Washington, United States of America; 3 The Henry M. Jackson Foundation for the Advancement of Military Medicine, Bethesda, Maryland, United States of America; 4 US Military HIV Research Program, Walter Reed Army Institute of Research, Silver Spring, Maryland, United States of America; 5 Duke University Human Vaccine Institute and the Center for HIV/AIDS Vaccine Immunology, Duke University School of Medicine, Durham, North Carolina, United States of America; 6 Duke University Medical Center, Durham, North Carolina, United States of America; 7 Department of Disease Control, Ministry of Public Health, Nonthaburi, Thailand; 8 Vaccine Trial Center, Faculty of Tropical Medicine, Mahidol University, Bangkok, Thailand; 9 Armed Forces Research Institute of Medical Sciences, Bangkok, Thailand; 10 Center of Excellence for Biomedical and Public Health Informatics, Faculty of Tropical Medicine, Mahidol University, Bangkok, Thailand; 11 Global Solutions for Infectious Diseases, South San Francisco, California, United States of America; 12 Department of Biomolecular Engineering, Baskin School of Engineering, University of California, Santa Cruz, California, United States of America; 13 Department of Retrovirology, Armed Forces Research Institute of Medical Sciences, Bangkok, Thailand; 14 Vaccine and Infectious Disease Division, Fred Hutchinson Cancer Research Center, Seattle, Washington, United States of America; 15 Department of Laboratory Medicine, University of Washington, Seattle, Washington, United States of America; 16 HIV Vaccine Trials Network, Vaccine and Infectious Disease Division, Fred Hutchinson Cancer Research Center, Seattle, Washington, United States of America; Rush University, UNITED STATES

## Abstract

**Background:**

In the HIV-1 vaccine trial RV144, ALVAC-HIV prime with an AIDSVAX^®^ B/E boost reduced HIV-1 acquisition by 31% at 42 months post first vaccination. The bivalent AIDSVAX^®^ B/E vaccine contains two gp120 envelope glycoproteins, one from the subtype B HIV-1 MN isolate and one from the subtype CRF01_AE A244 isolate. Each envelope glycoprotein harbors a highly conserved 27-amino acid HSV-1 glycoprotein D (gD) tag sequence that shares 93% sequence identity with the HSV-2 gD sequence. We assessed whether vaccine-induced anti-gD antibodies protected females against HSV-2 acquisition in RV144.

**Methods:**

Of the women enrolled in RV144, 777 vaccine and 807 placebo recipients were eligible and randomly selected according to their pre-vaccination HSV-1 and HSV-2 serostatus for analysis. Immunoglobulin G (IgG) and IgA responses to gD were determined by a binding antibody multiplex assay and HSV-2 serostatus was determined by Western blot analysis.

Ninety-three percent and 75% of the vaccine recipients had anti-gD IgG and IgA responses two weeks post last vaccination, respectively. There was no evidence of reduction in HSV-2 infection by vaccination compared to placebo recipients over 78 weeks of follow-up. The annual incidence of HSV-2 infection in individuals who were HSV-2 negative at baseline or HSV-1 positive and HSV-2 indeterminate at baseline were 4.38/100 person-years (py) and 3.28/100 py in the vaccine and placebo groups, respectively. Baseline HSV-1 status did not affect subsequent HSV-2 acquisition. Specifically, the estimated odds ratio of HSV-2 infection by Week 78 for female placebo recipients who were baseline HSV-1 positive (n = 422) vs. negative (n = 1120) was 1.14 [95% confidence interval 0.66 to 1.94, p = 0.64)]. No evidence of reduction in the incidence of HSV-2 infection by vaccination was detected.

**Conclusions:**

AIDSVAX^®^ B/E containing gD did not confer protection from HSV-2 acquisition in HSV-2 seronegative women, despite eliciting anti-gD serum antibodies.

## Introduction

The human herpes simplex virus type 2 (HSV-2) causes 50–80% of all genital ulcerative disease (GUD) and is rapidly acquired after sexual contact [1–4]. Moreover, HSV-2 infection is a major contributing risk factor for HIV-1 acquisition [5–10]. HSV-1 is also responsible for some of the burden of genital herpes in low- and middle-income countries and is similarly associated with GUD [3]. Only 20% of all persons who are HSV-2 seropositive report genital ulcerations, only 10% have a known diagnosis, and over 80% of all acquisitions are asymptomatic. This cycle of sub-clinical reactivation and acquisition has led to the continued spread and increasing worldwide burden of HSV-2, making an effective HSV vaccine an important but unmet need.

Multiple strategies have been taken in the pursuit of an HSV vaccine, including inactivated virus, recombinant subunit, and attenuated or replication incompetent live virus. The majority of vaccines in development have focused on the major surface HSV glycoproteins, which are known to harbor key epitopes that are targeted by HSV-neutralizing antibodies [11]. Here we focus our attention on recombinant surface HSV glycoprotein subunit vaccines; for further discussion of the other approaches please see [12, 13]. Five phase III trials of recombinant HSV glycoprotein subunit vaccines have been completed to date. The first two phase III efficacy trials were performed in parallel and tested a glycoprotein B (gB) and gD HSV-2 subunit vaccine formulated with the adjuvant MF59. Although high HSV-2-specific neutralizing antibody titers were elicited, the vaccine was not effective in reducing either HSV-2 acquisition or genital herpes disease [14]. Next, an HSV-2 gD subunit vaccine formulated with the adjuvants alum and 3-O-deacylated monophosphoryl lipid A (AS04) was tested in two phase III efficacy trials whose results were reported simultaneously [15]. In both trials, the gD/AS04 vaccine elicited significant protection against genital herpes disease in women who were seronegative for both HSV-1 and HSV-2 [73%, 95% confidence interval (CI) 19–91% and 74%, 95% CI 9–93%; both p<0.05]. However, the gD/AS04 vaccine did not provide any significant protection against genital herpes disease in either trial in women who were seropositive for HSV-1 and seronegative for HSV-2, nor was it efficacious in men (regardless of serostatus) in either trial. The fifth phase III trial was motivated by the previously demonstrated efficacy of the gD/AS04 vaccine in women who were seronegative for both HSV-1 and HSV-2 [15] and further evaluated the efficacy of this vaccine in this population; however, no discernable protection against HSV-2 acquisition or genital disease caused by either HSV type was observed [16]. Moreover, the vaccine actually increased the frequency of subsequent subclinical shedding [29% vs. 19%, relative risk 1.55, 95% CI 1.28 to 1.86], making further pursuit of the gD/AS04 vaccine unlikely. These results of these clinical trials were disappointing, considering that the gD/AS04 subunit vaccine conferred almost complete protection against primary disease in guinea pigs after challenge with either HSV-1 or HSV-2, albeit with highly passaged laboratory strains [17]. Moreover, passive transfer of antibodies against gD has been shown to be highly protective against disease and/or lethal infection in mice after challenge with a wide variety of HSV strains [18–20] and vaccination with recombinant gD protected mice against the establishment of latency by a neurovirulent HSV-1 strain (McKrae strain) [21]. Sequence differences between highly passaged laboratory strains, which are typically used in animal studies, and contemporary circulating strains to which humans are exposed may explain the discrepant results of vaccine efficacy studies in humans vs. animals [22].

**Fig 1:**
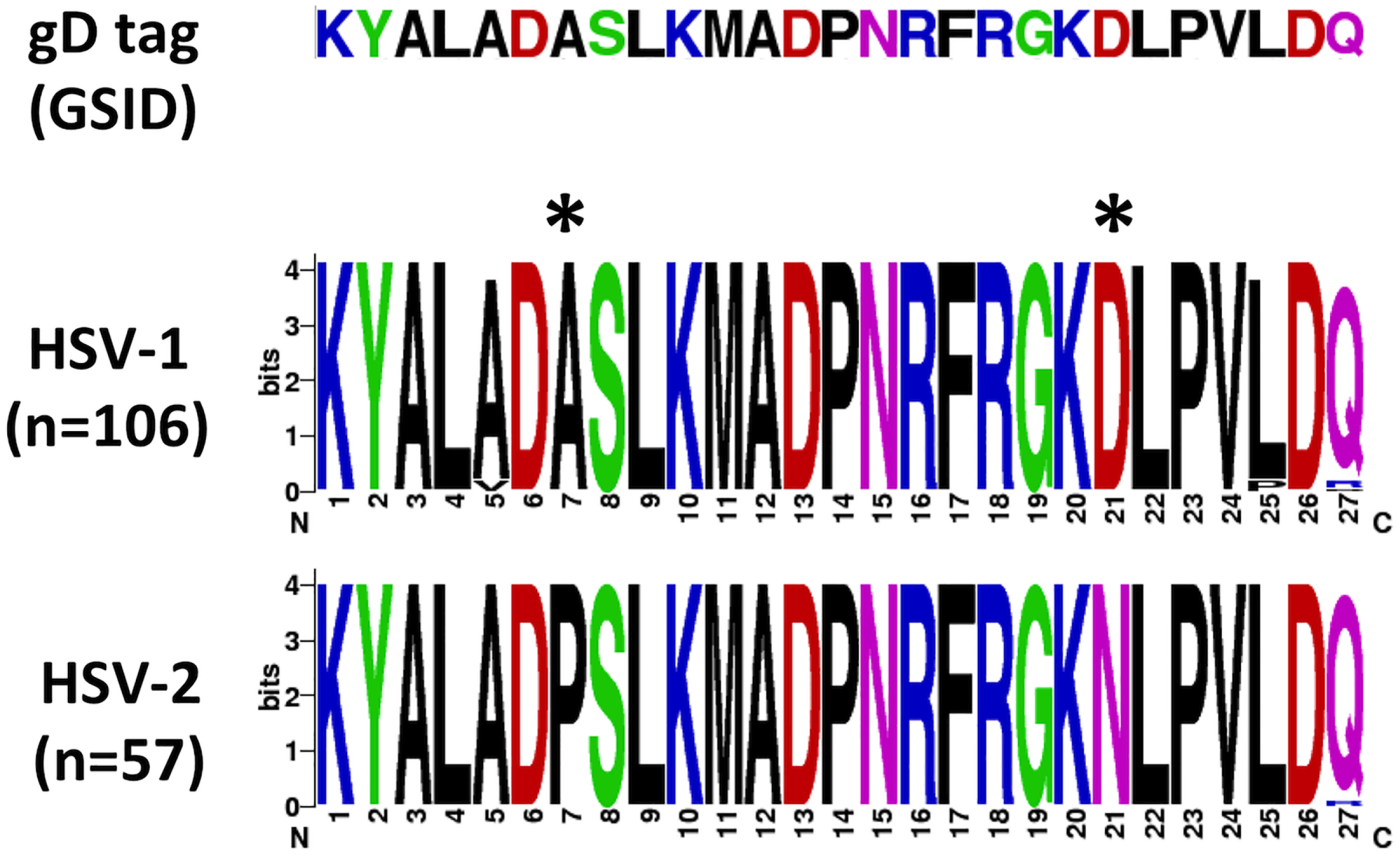
Comparison of the sequence of the gD tag expressed on the AIDSVAX^®^ B/E gp120 envelope glycoprotein antigens (Global Solutions for Infectious Diseases) with the corresponding published HSV-1 and HSV-2 sequences (retrieved from GenBank on 06/24/15). The amino acid sequences are shown in weblogos, depicting the different variants at each position as stacked symbols, with the height of each symbol indicating its relative frequency. Asterisks highlight the main differences between HSV-1 and HSV-2. Weblogos were constructed using [40].

In the HIV-1 vaccine trial RV144 (ClinicalTrials.gov NCT00223080), ALVAC-HIV prime with an AIDSVAX^®^ B/E boost reduced HIV-1 acquisition by an estimated 31% at 42 months post first vaccination [23–25]. Antibodies directed against the V1V2 epitope of the HIV-1 envelope protein gp120, likely induced by AIDSVAX^®^ B/E, were associated with a decreased risk of HIV-1 acquisition [24, 26]. AIDSVAX^®^ B/E contains two gp120 envelope glycoproteins, one from the subtype B HIV-1 MN isolate and one from the subtype CRF01_AE A244 isolate [27]. The MN and A244 gp120 glycoproteins from AIDSVAX^®^ B/E also each contain a highly conserved tag sequence consisting of the first 27 amino acids of HSV-1 gD (hereafter referred to as the gD peptide), which is used to facilitate expression and immunoaffinity purification during manufacturing [27, 28]. Due to cleavage of a signal sequence, the gD peptide is at the N terminus of the processed molecule. The HSV-1 gD peptide shares a high sequence identity with the HSV-2 gD peptide: specifically, 25/27 amino acids are identical. The only sequence differences occur at positions 7 (alanine to proline) and 21 (aspartic acid to asparagine) of the gD tag (Fig 1). This area is also known to be a defined T-cell epitope to HSV-2 as well as a target for a human monoclonal neutralizing antibody to gD2.

Somewhat unusually, the gD peptide contains overlapping T-cell and B-cell epitopes [29–32]. Specifically, this region contains defined CD4+ T-cell epitopes in mice [33, 34] (of note, one of these can protect against lethal HSV-1 challenge [35]) and humans [36, 37] and multiple B-cell epitopes recognized by mouse monoclonal antibodies against gD [31, 32, 38]. Moreover, the gD peptide is also a target of human HSV-neutralizing antibodies [39].

Although the AIDSVAX^®^ B/E gp120 glycoproteins contained the HSV-1 rather than the HSV-2 gD peptide, studies in mice [41] and guinea pigs [17, 42–44] have demonstrated cross-type protection against HSV infection acquisition and/or symptomatic HSV disease after immunization with recombinant HSV gD. Moreover, prior HSV-1 infection appears to provide some degree of protection against the development of symptomatic HSV-2 disease in humans [45, 46]. Considering these findings, we designed a retrospective evaluation to determine if the AIDSVAX^®^ B/E vaccine in the RV144 regimen elicited immune responses to HSV-2 and whether those immune responses could prevent HSV-2 acquisition. Women were preferentially selected for this analysis because of their possibly higher HSV-2 incidence [15] and because estimated VE against HSV-2 acquisition was higher in women than in men in a previous trial of a recombinant HSV-2 subunit vaccine [14]. We evaluated baseline (date of first HIV vaccination) and week 78 HSV-2 infections in female vaccine and placebo recipients in RV144 to determine whether the induced anti-gD responses might have protected women against incident HSV-2 infection over the first 18 months after the first vaccination.

## Materials and methods

### Study population and procedures

The RV144 trial was conducted in partnership with the Thai Ministry of Health in two southeastern provinces in Thailand (Rayong and Chon Buri). This study was a community-based, randomized, multicenter, double blind, placebo-controlled efficacy trial as described in S1 and S2 Files. The study inclusion criteria were: Thai citizen, 18–30 years old, male or female, available for participation for 3.5 years, passed a test of understanding about the trial and provided written informed consent, and completed enrollment in the associated screening protocol. The study exclusion criteria were: HIV-positive, previously participated in another HIV vaccine trial, active tuberculosis or other systemic disease, immunodeficiency or chronic use of immune modifying therapy, history of anaphylaxis or other serious adverse reactions to vaccines, and women breast feeding, pregnant, or planning to become pregnant during the first 9 months after enrollment [47]. A total of 16,402 volunteers were enrolled in the study; after excluding 7 volunteers who were found to be HIV-1 seropositive on the first test post-vaccination, 8197 volunteers were left in the vaccine group and 8198 volunteers were left in the placebo group. This group of 16,395 participants was 61.4% male and 38.6% female. Patients were enrolled from October 2003-December 2005 and followed up until June 2009 [47]. Further details are provided in [23, 47].

The prime-boost regimen consisted of two vaccines, ALVAC-HIV (vCP1521) (prime), a recombinant canarypox vaccine manufactured by Sanofi Pasteur, and AIDSVAX^®^ B/E (boost), a bivalent HIV-1 gp120 envelope glycoprotein vaccine developed by Global Solutions for Infectious Diseases. ALVAC-HIV was genetically engineered to express HIV-1 subtype B Gag and Pro and CRF01_AE gp120 linked to the transmembrane domain of gp41. AIDSVAX^®^ B/E was composed of recombinant CRF01_AE (A244) and subtype B (MN) gp120 envelope glycoproteins co-formulated with alum. Placebos for the two vaccines consisted of virus stabilizer/freeze drying medium in sodium chloride and alum adjuvant alone, respectively. The ALVAC-HIV vaccine (or placebo) was administered at baseline (day 0), 4 weeks, 12 weeks, and 24 weeks. The AIDSVAX^®^ B/E boost (or placebo) was co-administered at weeks 12 and 24. Further details are provided in [23, 47]. The authors confirm that all ongoing and related trials for this intervention are registered.

### Definitions of cohorts for analysis

#### Anti-gD responses cohort

Prior to analysis for immune correlates of HIV-1 infection, the assays under consideration were tested for detectability, reproducibility, and dynamic range, based on samples from random sets of HIV-1-uninfected participants (80 vaccine and 20 placebo recipients, split evenly among males and females) distributed to labs. In addition, samples from a random set of 205 HIV-1 uninfected vaccine recipients and 20 HIV-1 uninfected placebo recipients were generated for the RV144 case-control study [26]. Of these recipients, 75/205 (vaccinated) and 10/20 (placebo) participants were female and their samples were used for characterizing anti-gD responses. Data were uploaded to a database maintained by the Statistical Center for HIV/AIDS Research and Prevention (SCHARP).

#### Study cohort for HSV testing and assessing vaccine efficacy

**Fig 2:**
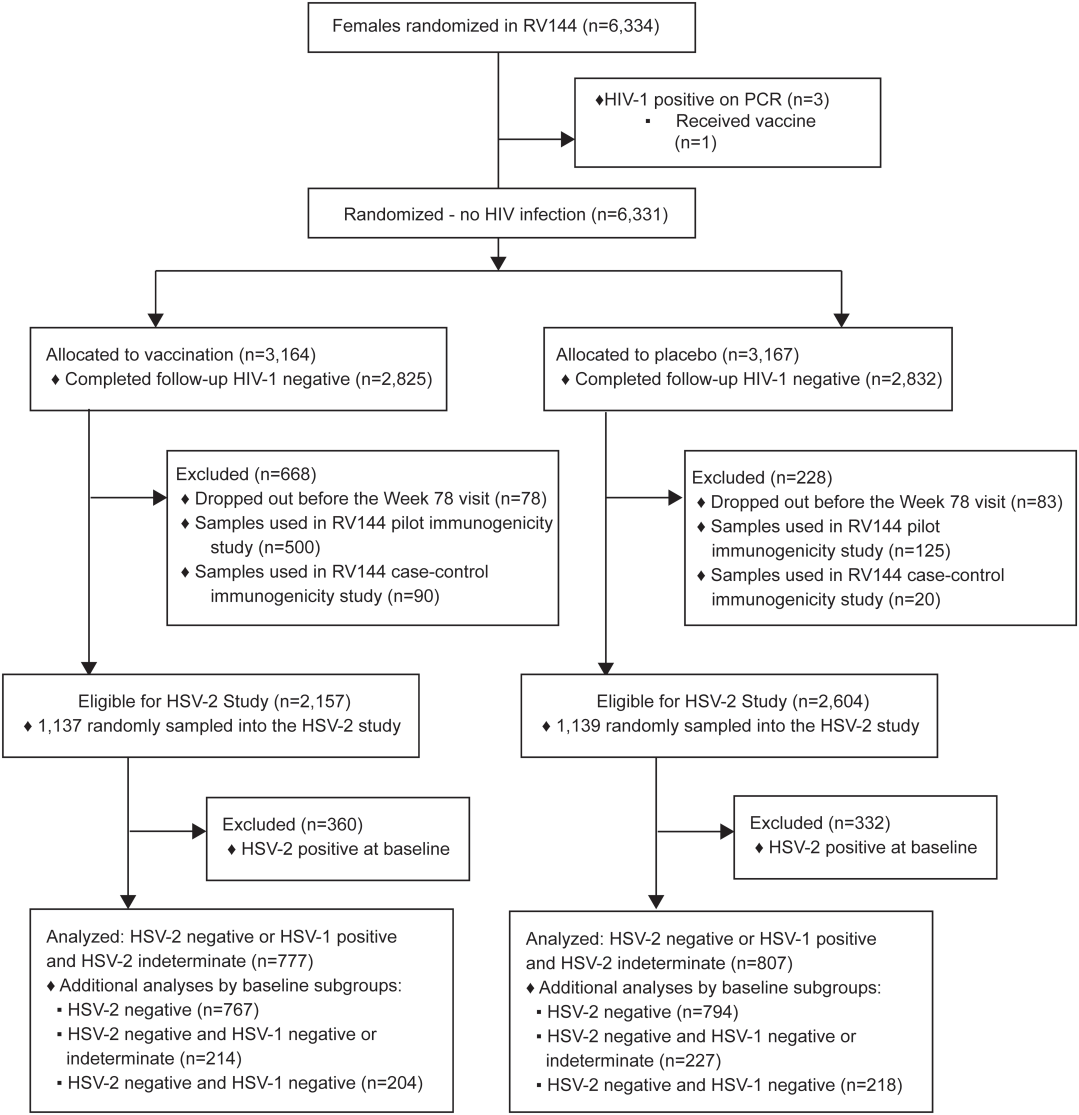
CONSORT diagram.

A total of 6,334 women were randomized in RV144 (Fig 2). After excluding women with HIV-1 infection and/or who did not complete the study through Week 78, or who were included in the immune correlates of HIV-1 risk studies, 2,157 and 2,604 women assigned to vaccine and placebo, respectively, were eligible for the present study. From these women, samples from 1,478 and 1,529 vaccine and placebo recipients, respectively, were randomly selected for HSV-1 and HSV-2 serotesting at baseline and Week 78. For each HSV type, participants were scored as having positive, negative, or indeterminate serostatus. These tests revealed that 777 vaccine recipients and 807 placebo recipients were highly likely to be HSV-2 uninfected at baseline based on being HSV-2 negative or HSV-1 positive and HSV-2 indeterminate (Cohort 1). As a sensitivity analysis, all HSV-2 seronegative women were additionally classified into one or more of the following cohorts based on their HSV-1 serostatus: Cohort 2, HSV-2 negative regardless of HSV-1 status (HSV-1 negative, indeterminate, or positive; this cohort included all HSV-2 negative women); Cohort 3, HSV-2 negative and HSV-1 negative or indeterminate (as in past analyses); and Cohort 4, HSV-2 negative and HSV-1 negative (double negative). These four cohorts are nested with Cohort k a subset of Cohort k−1 for each k = 2,3,4.

For each cohort, HSV-2 infection over 18 months was measured in two ways. In the first approach, HSV-2 infection was defined as being HSV-2 positive or indeterminate at the Week 78 visit (where an indeterminate result likely reflects a recent HSV-2 infection). In the second, more stringent approach, HSV-2 infection was defined as being HSV-2 positive at the Week 78 visit.

### Anti-gD HSV binding assays

Immunoglobulin G (IgG) and IgA responses to gD were determined by a binding antibody multiplex assay (BAMA) as previously described [26, 48, 49]. Responses to HSV gD after vaccination were considered positive if they met the pre-specified antigen-specific threshold and were 3-fold over the baseline values (pre-vaccination).

### HSV serology

HSV-2 serostatus was determined by Western blot analysis at the University of Washington as previously described [50]. Briefly, sera from patients were tested for antibodies specific for HSV-2 glycoprotein G (gG). Control serum pools were reacted with HSV-1 and HSV-2 antigens in each staining run. Each pool consisted of sera from 10 individuals with culture-confirmed and typed HSV isolates. In addition, in each run, an HSV-2 Western blot was performed with the monoclonal antibody AP1, which reacts primarily with the 92,000-Mr form of gG-2 [51].

### Statistical analysis

For each of the four cohorts and the two incident HSV-2 endpoint definitions, vaccine efficacy was assessed with a univariable logistic regression model by estimating the odds ratio (OR) of HSV-2 infection by Week 78 (vaccine vs. placebo), with a 95% CI and a 2-sided p-value for whether the OR differed from 1 (fit with the glm function in R version 3.2.0). An estimated OR below 1 is in the direction of vaccine protection against HSV-2. All statistical analyses were performed using R software version 3.2.0. All p-values were 2-sided.

### Sample size and power

The sample size of females sampled into Cohort 1 (baseline HSV-2 negative or HSV-1 positive and HSV-2 indeterminate) was chosen to achieve at least 80% power to reject the null hypothesis of zero vaccine efficacy against HSV-2 infection in favor of vaccine efficacy equal to 50%, based on a 2-sided 0.05-level Wald test in a logistic regression model for testing whether the odds ratio of HSV-2 infection by 18 months (vaccine vs. placebo) differs from unity. The power calculation method of Demidenko (2007) was used [52]. Successive batches of placebo recipient samples (with a smaller number of vaccine recipient samples for blinding) were tested for HSV-1 and HSV-2 status at baseline and Month 18 to learn about the rate of HSV-2 infection by 18 months in Cohort 1, and batches were stopped once there was enough data to achieve the desired power after testing approximately the same number of vaccine recipient samples for HSV-1/HSV-2 status. With 800 participants in each of the Cohort 1 vaccine and placebo groups and the observed 6.7% HSV-2 infection rate in the placebo group, there is 82% power to reject the null hypothesis if the odds ratio is one-half (equivalently, vaccine efficacy is 50%).

### Ethics

RV144 (ClinicalTrials.gov NCT00223080) was reviewed and approved as reported previously [23] by the Ethics Committees of the Human Subjects Research Review Board of the U.S. Army Medical Research and Materiel Command (September 23, 2003), Mahidol University (October 14, 2003), Royal Thai Army (September 18, 2003), and the Thai Ministry of Public Health (October 8, 2003). Written informed consent was obtained from all study participants. The first participant was vaccinated on October 20, 2003.

## Results

### Cohort characteristics

Table 1 describes the distributions of age and baseline self-reported behavioral risk score by vaccine and placebo group, for each of the four study cohorts of putatively baseline HSV-2 uninfected females included in HSV-2 vaccine efficacy analyses. It shows an approximately even distribution of ages in the three protocol-specified categories 18–20, 21–25, 26–30, a little more than half of participants have low behavioral risk with the remainder divided about equally between the medium and high behavioral risk categories. Table 1 also includes the distribution of these factors for females in the entire RV144 study, to compare the study populations for assessing HSV-2 vaccine efficacy and HIV-1 vaccine efficacy in the original RV144 study. It shows that the age distributions are fairly similar, whereas the HSV-2 cohorts had lower baseline risk scores than the whole RV144 cohort. This may be explained by the fact that the extra eligibility criterion of being diagnosed as HSV-2 uninfected at baseline selects for lower risk women. This result that the analyzed HSV-2 cohort is not a representative sample of the whole RV144 study population does not affect the validity of inferences from the analysis− given the randomization to vaccine versus placebo− rather the implication is that the inferences about HSV-2 vaccine efficacy are for a lower risk population than the original RV144 study population.

**Table 1:** Distribution of age (≤20, 21–25, ≥26)* and baseline self-reported behavioral risk score (Percent Low, Medium, High)** for female RV144 participants with HSV measurements included in the study.

Variable	Cohort 1 Vaccine, Placebo (Total %)	Cohort 2 Vaccine, Placebo (Total %)	Cohort 3 Vaccine, Placebo (Total %)	Cohort 4 Vaccine, Placebo (Total %)	RV144 (All) Vaccine, Placebo (Total %)
Age group					
≤ 20 yr	309, 342 (41%)	304, 338 (41%)	74, 93 (38%)	70, 86 (37%)	1346, 1424 (44%)
21–25 yr	256, 271 (33%)	254, 268 (33%)	90, 95 (42%)	86, 94 (43%)	816, 796 (25%)
≥ 26 yr	212, 194 (26%)	209, 188 (25%)	50, 39 (20%)	48, 38 (20%)	1002, 947 (31%)
Risk score					
Low	414, 422 (53%)	412, 414 (53%)	125,135 (59%)	118, 130 (59%)	724, 692 (22%)
Medium	178, 210 (25%)	173, 207 (24%)	42, 56 (22%)	41, 52 (22%)	1567, 1560 (49%)
High	185, 175 (23%)	182, 173 (23%)	47, 36 (19%)	45, 36 (19%)	873, 915 (28%)

*Age categories used in Rerks-Ngarm et al. [23–25].

**Low, medium, high self-reported risk as defined in Rerks-Ngarm et al. [23–25].

### Responses to gD in the RV144 vaccinated cohort

**Fig 3:**
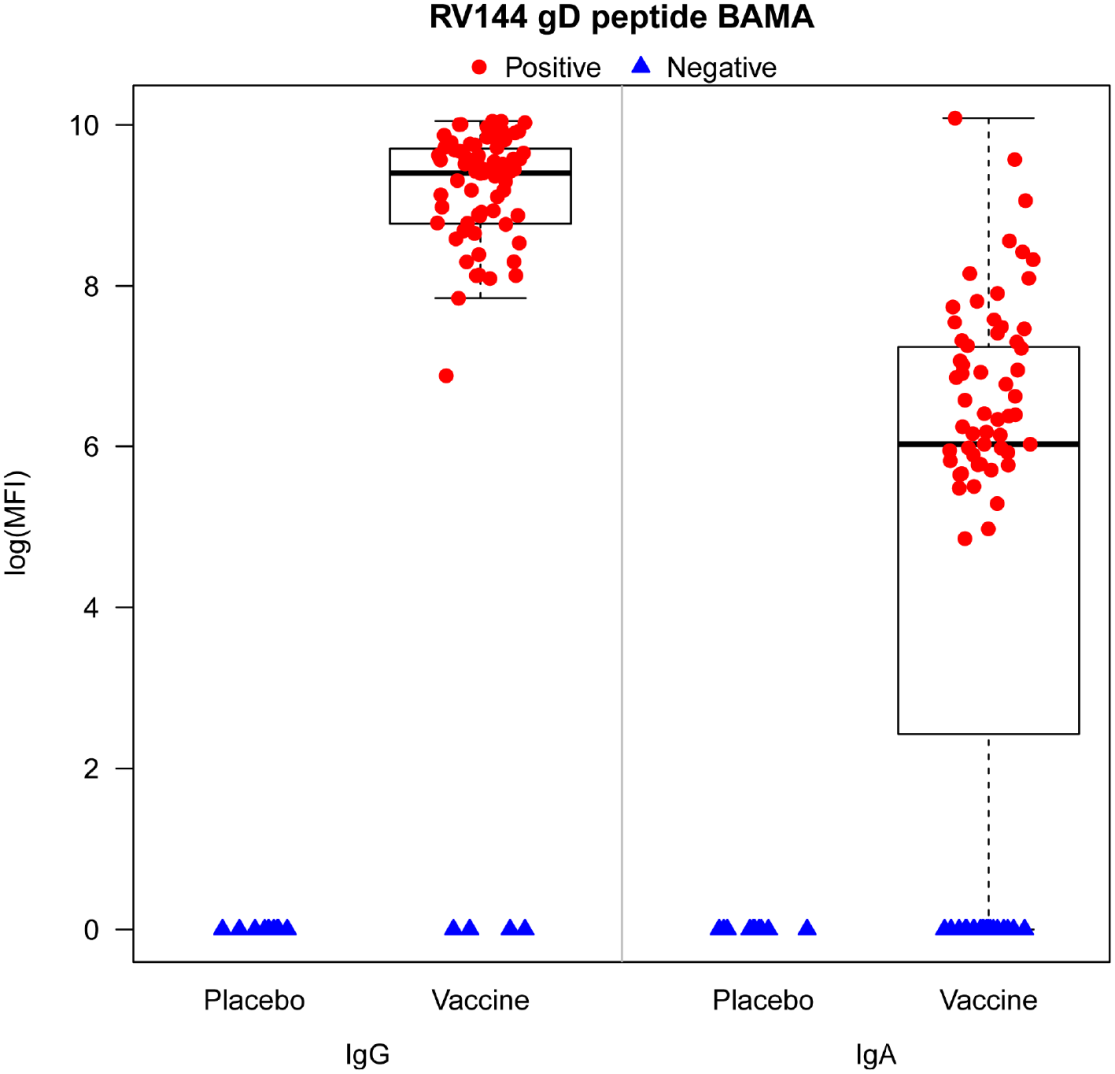
IgG and IgA responses and percentage of responders to the gD protein measured at week 26 by binding antibody multiplex array for the n = 85 HIV-1-uninfected female participants in the RV144 case-control study [26] (75 vaccine and 10 placebo recipients).

We have previously shown that antibodies to gD were not associated with risk of HIV-1 infection [26, 53]. Based on the case-control sample of HIV-1 uninfected RV144 female participants, we assessed anti-gD antibody responses at baseline and 2 weeks post final vaccination (Week 26) in 75 vaccine and 10 placebo recipients. Week 26 anti-gD responses were significantly higher in vaccine than placebo recipients for both IgG and IgA (p < 0.001, Wilcoxon rank sum test with a normal approximation critical value implemented with Wilcoxon test in R version 3.2.0) (Fig 3). There were no positive responses among placebo recipients, and 93.3% (95% CI 87.6% to 99.0%) and 74.7% (95% CI 64.9% to 84.5%) of all vaccine recipients had positive IgG and IgA responses, respectively. In addition, the magnitude of the IgG response was greater than that of the IgA response, with median response of 9.4 and 6.0 log (mean fluorescence intensity), respectively (Fig 3).

### Primary analysis

No evidence of reduction in HSV-2 infection by vaccination was seen in any of the 4 cohorts using either the broad (Table 2) or more stringent (Table 3) definition of HSV-2 infection (p-values > 0.50). By the first and second definitions, the estimated ORs for HSV-2 acquisition were 0.96 (95% CI 0.64 to 1.43, p = 0.84) and 1.16 (95% CI 0.73 to 1.83, p = 0.52), respectively. Moreover, by the first definition, the annual incidences of HSV-2 infection for Cohort 1 were 4.38/100 person-years (py) and 3.28/100 py for the vaccine and placebo groups, respectively.

**Table 2:** Comparison of HSV-2 infection rates by week 78 (Vaccine vs. Placebo Female Recipients) (HSV-2 Infection = Positive or Indeterminate at Week 78).

Cohort	HSV-2 Infection Rate*		Est. OR	95% CI	2-sided P-value
	Vaccine	Placebo			
1	50/777 (6.44%)	54/807 (6.69%)	0.96	0.64–1.43	0.84
2	40/767 (5.22%)	41/794 (5.16%)	1.01	0.65–1.58	0.96
3	10/214 (4.67%)	13/227 (5.73%)	0.81	0.35–1.88	0.62
4	10/204 (4.90%)	13/218 (5.96%)	0.81	0.35–1.90	0.63

*The second and third columns show HSV-2 infection counts, numbers of individuals in the analysis, and percent infected.

**Table 3:** Comparison of HSV-2 infection rates by week 78 (Vaccine vs. Placebo Female Recipients) (HSV-2 Infection = Positive at Week 78).

Cohort	HSV-2 Infection Rate*		Est. OR	95% CI	2-sided P-value
	Vaccine	Placebo			
1	41/777 (5.28%)	37/807 (4.58%)	1.16	0.73–1.83	0.52
2	35/767 (4.56%)	32/794 (4.03%)	1.14	0.70–1.86	0.60
3	9/214 (4.21%)	11/227 (4.85%)	0.86	0.35–2.12	0.75
4	9/204 (4.41%)	11/218 (5.05%)	0.87	0.35–2.14	0.76

*The second and third columns show HSV-2 infection counts, numbers of individuals in the analysis, and percent infected.

### HSV-1 infection not associated with HSV-2 acquisition

The incidence of HSV-2 acquisition was similar in placebo recipients with prior HSV-1 antibodies (Cohorts 1 and 2) vs. placebo recipients who were double seronegative (Cohorts 3 and 4), suggesting that HSV-1 did not protect against HSV-2 acquisition in Thai women. In particular, the estimated OR of HSV-2 (with the HSV-2 positive at Week 78 endpoint definition) for females who were HSV-1 positive (n = 422) vs. negative (n = 1120) at baseline was 1.14 (95% CI 0.66 to 1.94, p = 0.64).

### HIV-1 risk and HSV-2 infection

In RV144, risk factors for HIV-1 infection were collected at baseline and at 6-month intervals [23, 54]. Of the 1,584 participants in the analysis, 836 (52.8%), 388 (24.5%), and 360 (22.7%) were in the low, medium, and high baseline risk groups (as defined previously [23]). In the vaccine group, 414 (53.3%), 178 (22.9%), and 185 (23.8%) recipients were in the low, medium, and high baseline risk groups, respectively. For the placebo group, 422 (52.3%), 210 (26.0%), and 175 (21.7%) were in the low, medium, and high baseline risk groups, respectively.

The HSV-2 infection incidence was lowest in the group reporting the low HIV infection risk, and rose in both the medium and high-risk groups (Table 4). A logistic regression model to assess baseline behavioral risk as a predictor of incident HSV-2 infection considering the vaccine and placebo groups in aggregate showed a higher HSV-2 infection rate in the medium and high groups compared to the low group (overall 2-sided Wald p-value = 0.001). The estimated OR of HSV-2 (with the HSV-2 positive at Week 78 endpoint definition) was 2.73 for medium vs. low (95% CI 1.57 to 4.76) and 2.52 for high vs. low (95% CI 1.42 to 4.48).

**Table 4:** HSV-2 infection rates by baseline behavioral HIV-1 risk score (Primary Cohort).

	Baseline Behavioral HIV-1 Risk Score		
	Low	Medium	High
Vaccine Group	14/414 (3.38%)	12/178 (6.74%)	15/185 (8.11%)
Placebo Group	10/422 (2.37%)	17/210 (8.10%)	10/175 (5.71%)
Both Groups Pooled	24/836 (2.87%)	29/388 (7.47%)	25/360 (6.94%)

## Discussion

Our study evaluated whether an HIV vaccine regimen that included the HSV-1 gD tag at the N-terminus of gp120 elicited responses that could protect females against HSV-2 infection [26, 53]. The RV144 vaccine regimen was unique in that it elicited a high frequency of both IgG and IgA responses to HSV gD, in addition to high responses to HIV antigens. Since the HSV-1 gD sequence included in the vaccine regimen is highly conserved between HSV-1 and HSV-2 and represents a virus sequence critical for HSV cell entry, we hypothesized that vaccine-induced antibody responses to HSV-1 gD might be protective against HSV-2 acquisition. Our hypothesis was also supported by animal studies demonstrating that immunization with recombinant HSV gD confers cross-type protection against HSV infection acquisition and/or severity of symptomatic infection after experimental challenge [17, 41–44]. However, although circulating gD antibodies were detectable in participant serum samples, we found no evidence of protection from HSV-2 in this cohort over the first 18-month period of the study or in the 12 months post last vaccination.

With 104 total incident HSV-2 infection events, this cohort study nested within RV144 had reasonable precision to assess vaccine efficacy in HSV-2 seronegative women, where with approximately 800 participants in each of the vaccine and placebo groups and a 6.7% HSV-2 infection rate in the placebo group (Table 2), there was 82% power to reject the null hypothesis of zero vaccine efficacy in favor of vaccine efficacy equal to 50% (2-sided 0.05-level test of the ORs in a logistic regression model differing from unity). The point estimate and 95% CI for vaccine efficacy − estimated vaccine efficacy = 4% with 95% CI -43% to 36% − demonstrates that vaccine efficacy to prevent HSV-2 infection was low at best, and supports lack of any vaccine efficacy. However, we cannot determine whether potentially protective antibodies for HSV-2 infection were elicited, but were either not of high enough magnitude and/or were not present at the appropriate locations (i.e., mucosal surfaces and tissues). Ongoing work examining the B-cell repertoire of the RV144-elicited humoral response has identified and produced HSV gD-specific monoclonal antibodies (mAbs) (M.A. Moody, personal communication). These RV144-derived gD mAbs can now be tested in small animal studies to examine protection in a setting where concentration and mAb antibody dose can be controlled and measured. Such studies promise to help determine the potential of these vaccine-elicited gD antibodies.

This study was prospectively powered for assessing vaccine efficacy in HSV-2 seronegative women, not in women seronegative to both HSV-1 and HSV-2, for whom there were only 23 incident HSV-2 infection events and the 95% confidence interval about vaccine efficacy was wide: estimated vaccine efficacy = 19%, 95% CI -90% to 65%. Therefore, this study is inconclusive about vaccine efficacy in double HSV-1/HSV-2 seronegative women. It is not possible to adequately answer this objective, given that 63% of the eligible female participants were included in the study and adding the additional 37% by running additional HSV-1/HSV-2 tests would still yield an underpowered assessment. As discussed in the introduction, five earlier randomized controlled trials of a subunit vaccine containing gD did not support overall efficacy against HSV-2, consistent with our findings [15][16]. For the current study, we prioritized studying vaccine efficacy in HSV-2 seronegative women instead of in double HSV-1/HSV-2 seronegative women because HSV-2 causes more morbidity through genital disease and is a more important risk factor for HIV-1 infection.

A pilot study of 500 men and women from the placebo group of RV144 showed an annual HSV-2 incidence of 3.9% in women and 2.4% in men. A decision was therefore made to limit the analysis of the impact of vaccination on HSV-2 infection to women only. The annual incidence of HSV-2 infection (4%) in this seronegative cohort suggests that future HSV-2 vaccine trials might be conducted in this population.

Baseline HIV-1 risk was correlated with HSV-2 acquisition. HSV-2 infection rates were lowest in those with low baseline HIV-1 risk and were higher in those with higher HIV-1 risk. Conversely, the burden of HSV infection worldwide is significant and appears to increase the risk of HIV-1 infection. Like HIV-1 infection, both barrier protection and treatment lower the risk of infection, and a vaccine would be an important part of a comprehensive solution.

## Supporting information

S1 File RV144 protocol V3.8 1Dec14 CL. (PDF)

S2 File CONSORT 2010 checklist. (PDF)
