# Supplementary material for: Antibody to HSV gD peptide induced by vaccination does not protect against HSV-2 infection in HSV-2 seronegative women
Source: PLoS One. 2017 May 11;12(5):e0176428. doi: 10.1371/journal.pone.0176428 (PMC5426618; doi:10.1371/journal.pone.0176428)
Supplement: S1 File — (PDF) [file pone.0176428.s001.pdf]

CLINICAL STUDY PROTOCOL RV144

A Phase III Trial of Aventis Pasteur Live Recombinant ALVAC-HIV (vCP1521) Priming With VaxGen gp120 B/E (AIDSVAX® B/E) Boosting in HIV-uninfected Thai Adults

Principal Investigator:
Dr. Supachai Rerks-Ngarm
Department of Disease Control
Ministry of Public Health
C/O Nonthaburi, Thailand

Tel: 66 2 590 3006
Fax: 66 2 965 9569

Sponsor (Study IND Holder):
[BB-IND 8795 – held by OTSG]
Kenneth A. Bertram, MD, PhD
U.S. Army Medical Research and Materiel Command (USAMRMC)
1430 Veterans Drive
Fort Detrick, MD 21702-5009, USA
Tel: +1 301 619 0317; DSN: 343-0317
Fax: +1 301 619 0197
usarmy.detrick.medcom-usammda.mbx.usamrmc-regulatory-affairs@mail.mil

Vaccine Manufacturers:
sanofi pasteur
Discovery Drive
Swiftwater, PA 18370
Contact: Dr. John McNeil
Tel: 1 570 839 6185
Fax: 1 570 839 0934

Global Solutions for Infectious Diseases
800 Dubuque Ave. South San Francisco, CA 94080Contact: Dr. Don Francis
Tel: 1 650 228-7900
Fax: 1 650 228-7901

Clinical Sites:
Hospitals, Health Centers and Rayong STD Clinic, of the Ministry of Public Health located in Rayong and Chon Buri Provinces, Thailand

Confidential
The information contained herein is the property of Ministry of Public Health of Thailand, the U.S. and Royal Thai Armies, Mahidol University, Aventis Pasteur, VaxGen, Inc., the U.S. National Institutes of Health and the Henry M. Jackson Foundation, and may not be reproduced, published or disclosed to others without written authorization.

PROTOCOL SUMMARY

TITLE: A Phase III Trial of Aventis Pasteur Live Recombinant ALVAC-HIV (vCP1521) Priming With VaxGen gp120 B/E (AIDSVAX® B/E) Boosting in HIV-uninfected Thai Adults

SPONSOR: Office of The Surgeon General, U.S. Army Medical Department

OBJECTIVES:

Primary Objectives

To determine whether immunizations with an integrated combination of ALVAC-HIV (vCP1521) boosted by AIDSVAX® gp120 B/E prevent HIV infection in healthy Thai volunteers.
And
To determine whether immunization with this vaccine combination results in reduced HIV viral load among those acquiring HIV-1 infection, comparing vaccine recipients to placebo recipients.

Secondary Objectives

- To determine whether immunization with this vaccine combination results in an increased CD4 count among those acquiring HIV-1 infection, comparing vaccine recipients to placebo recipients.
- To confirm the safety of this vaccine combination in Thai volunteers.
- To evaluate whether participation in this HIV vaccine trial is associated with behavior change that may increase the risk of HIV infection.

SUBJECTS: 16,000 HIV-uninfected Thai subjects, male or female, aged 18 through 30 years (inclusive), available for 3.5 years of participation.

STUDY SITES: Ministry of Public Health (MOPH) facilities in Chon Buri and Rayong Provinces, Thailand, to include health centers, hospitals, and Rayong STD Clinic (See Appendix 17.2).

PRODUCT DESCRIPTIONS:

ALVAC-HIV (vCP1521), produced by Aventis Pasteur (Marcy L'Etoile, France), is a recombinant canarypox vector vaccine that has been genetically engineered to express subtype E HIV-1: gp120 (subtype E) linked to the transmembrane anchoring portion of gp41 (subtype B), and HIV-1 gag and protease (subtype B). ALVAC-HIV (vCP1521) is formulated at a dose of >10^6 $CCID_{50}$. The diluent supplied for reconstitution of ALVAC-HIV (vCP1521) consists of sterile 0.4% NaCl.

ALVAC Placebo (Aventis Pasteur) is supplied as a sterile, lyophilized product that consists of a mixture of virus stabilizer, and freeze drying medium. The diluent supplied for reconstitution of ALVAC-Placebo consists of sterile 0.4% NaCl.

AIDSVAX® B/E, produced by VaxGen, Inc. (Brisbane, CA), is a bivalent HIV gp120 envelope glycoprotein vaccine containing a subtype E envelope from the HIV-1 strain A244 and a subtype B envelope from the HIV-1 strain MN. The recombinant gp120s are produced in genetically engineered Chinese hamster ovary (CHO) cell lines. The envelope glycoproteins are coformulated and administered at a combined dose of 600 μg (300 μg of each antigen). AIDSVAX® B/E is formulated with 600 μg of alum adjuvant.

AIDSVAX Placebo (VaxGen, Inc.) is 600 μg alum adjuvant.

ROUTE OF ADMINISTRATION: Intramuscular into the deltoid muscle: ALVAC-HIV or ALVAC Placebo (1 mL) into left deltoid; AIDSVAX® B/E or AIDSVAX Placebo (1 mL) into right deltoid.

STUDY ENDPOINTS

Primary Endpoints

The acquisition of HIV infection as determined by repeatedly reactive EIA, positive Western blot, and positive HIV nucleic acid testing (from two different blood collections) as detailed in Section 6.7.3.

and

The determination of plasma viral load in volunteers developing HIV infection during the trial.

Secondary Endpoints

The secondary endpoints of the trial are:

- CD4 T cell count in volunteers developing HIV infection during the trial.
- Safety assessment of this vaccine combination in Thai volunteers
- Change in HIV risk behaviors associated with participation in the vaccine trial.

OVERALL STUDY DESIGN AND PLAN

Vaccine Trial Design

		Weeks			
Group	Number	0	4	12	24
I	8,000	ALVAC Placebo	ALVAC Placebo	ALVAC Placebo + AIDSVAX Placebo	ALVAC Placebo + AIDSVAX Placebo
II	8,000	ALVAC-HIV	ALVAC-HIV	ALVAC-HIV + AIDSVAX® B/E	ALVAC-HIV + AIDSVAX® B/E

Study Design: This will be a community-based, randomized (vaccine:placebo = 1:1), multicenter, double-blind, placebo-controlled clinical trial. Screening of potential volunteers will be carried out under a separate protocol entitled "Screening and evaluation of potential volunteers for a trial in Thailand of a candidate preventive HIV vaccine" (RV148). Eligible volunteers will be enrolled over approximately two years. The statistical assumptions of the study will require that 16,000 persons enroll into the study. For purposes of sample size calculations the dropout rate was assumed to be 5% per 6-month period, including the vaccination phase. If the rate of dropout during immunization exceeds 5%, additional subjects will be recruited and enrolled to achieve 15,200 volunteers completing the 24-week vaccination phase. If it becomes obvious that 15,200 volunteers will not complete the vaccine phase in a timely manner, after 16,000 volunteers have been enrolled an instruction will be sent out to the sites to stop screening. Those already screened will be allowed to enroll so slightly more than 16,000 will finally be enrolled. The concern about the sample size will be balanced by increased efforts in the tracking and retention of those volunteers who have completed the vaccine phase. Vaccinations for each individual will occur over a 24-week period (0, 4, 12, 24 weeks). Women will be tested for pregnancy and pregnant volunteers will not be vaccinated. The volunteers will be followed with HIV testing every 6 months for 3 years after immunization. Blood will be collected for plasma (for diagnostics and HIV-specific antibodies) at 0, 24 and 26 weeks, and every 6 months during the follow-up phase. The blood collection at 0, 12 and 42 (and potentially 6) months will also be used for cryopreservation and archiving of PBMCs (for HIV-specific cellular immune responses). At week 24 and at each six-month follow-up visit, volunteers will have HIV testing, preceded by pretest counseling and followed (approximately 2-3 weeks later) by post-test counseling. Assessment of HIV risk behavior will be performed at baseline, week 26 and at each 6-month follow-up visit. Education on risk behavior reduction will be given at each vaccination visit and at each post-test counseling visit.

TABLE OF CONTENTS

1. LIST OF ABBREVIATIONS AND DEFINITIONS OF TERMS ... 7
2. ETHICS ... 9
  2.1 Institutional Review Boards (IRBs) ... 9
  2.2 Test of Understanding (will be performed in the screening protocol, RV148) ... 9
  2.3 Informed Consent ... 9
  2.4 Data and Safety Monitoring Board ... 10
3. INVESTIGATORS AND STUDY ADMINISTRATIVE STRUCTURE ... 11
4. BACKGROUND AND RATIONALE ... 13
  4.1 Trial ... 19
5. OBJECTIVES ... 25
  5.1 Primary Objectives ... 25
  5.2 Secondary Objectives ... 25
6. INVESTIGATIONAL PLAN ... 25
  6.1 STUDY DESIGN AND PLAN ... 25
  6.2 RATIONALE FOR THE STUDY ARMS ... 26
  6.3 SELECTION OF STUDY POPULATIONS ... 26
    6.3.1 Inclusion Criteria ... 27
    6.3.2 Exclusion Criteria ... 27
    6.3.3 Discontinuation of Subjects from Trial or Assessment ... 28
  6.4 VACCINES AND PLACEBOS AND THEIR ADMINISTRATION ... 30
    6.4.1 Summary of Vaccines and Placebos ... 30
    6.4.2 Identity of Investigational Products ... 30
      6.4.2.1 Vaccine Supplies ... 31
      6.4.2.2 Reconstitution of Vaccines/Placebos ... 31
      6.4.2.3 Dosage Administration ... 32
      6.4.2.4 Disposition ... 32
      6.4.2.5 Precautions to be Observed in Administering Study Vaccine ... 33
    6.4.3 Method of Assigning Subjects to Study Groups ... 33
    6.4.4 Blinding Procedure ... 33
    6.4.5 Prior and Concomitant Medications and Vaccines ... 34
  6.5 REFERRAL AND MANAGEMENT OF HIV-INFECTED VOLUNTEERS ... 35
    6.5.1 Referral and Follow-up Procedures ... 35
    6.5.2 Treatment of Volunteers HIV Infected during Trial ... 35
  6.6 Study Procedures at Each Visit ... 36
  6.7 Study Variables and Their Measurement ... 39
    6.7.1 Study Primary Endpoints ... 39
    6.7.2 Safety Assessments ... 40
      6.7.2.1 Post-vaccination Events Monitored as Possible Reactions to Vaccine ... 40
      6.7.2.2 Other Adverse Events ... 40
    6.7.3 HIV Diagnostic Algorithm (see flow diagram below and Section 9.2) ... 43
  6.8 PROCEDURAL FLOW CHART ... 46
  6.9 ANALYTIC PLAN ... 47
    6.9.1 Intent-to-Treat vs. Per-Protocol Analyses ... 47
    6.9.2 Sample Size, Accrual and Follow-up ... 47

6.9.3 Analysis of Primary Endpoints 48
6.9.3.1 Primary Efficacy Analysis 48
6.9.3.2 Viral load 51
6.9.4 Analysis of Secondary Endpoints 53
6.9.4.1 Safety and Risk Behavior Data 53
6.9.4.2 CD4 T cell counts. 54
6.9.4.3 Correlates of protection and supplemental analyses. 55
6.9.5 Interim Efficacy Analysis (for interim safety analyses, see Section 6.9.4.1) 55
6.9.6 Interim Futility Analysis 56
6.9.7 Additional Laboratory Studies and Data Analysis 56
6.10 DATA MANAGEMENT PLAN 57
7. BENEFITS, COMPENSATION, RESEARCH-RELATED ILLNESS/INJURY, CONFIDENTIALITY AND ADVOCACY 58
7.1 Benefits 58
7.1.1 Benefits and Risks to Volunteers who Become Prisoners 59
7.2 Compensation 59
7.3 Research-related Illness/Injury 59
7.4 Confidentiality 59
7.5 Advocacy 59
8. VACCINE ACCOUNTABILITY 60
9. LABORATORY REQUIREMENTS 60
9.1 Specimen Processing and Storage 60
9.2 Laboratory Assays 61
10. CASE REPORT FORMS 63
11. STUDY MONITORING AND COMPLIANCE 63
12. RETENTION OF RECORDS 64
13. USE OF INFORMATION AND PUBLICATION 64
14. PROTOCOL AMENDMENTS 64
15. REFERENCES 65
16. SIGNATURE PAGE 73
17. APPENDICES 74
17.1 STATEMENT OF OBLIGATIONS OF CLINICAL MONITORS, CLINICAL INVESTIGATORS AND MEDICAL MONITORS 74
17.2 VACCINE TRIAL STUDY SITES 76
17.3 INFORMED CONSENT PROCESS AND CONSENT FORM (attached) 76
17.4 TEST OF UNDERSTANDING (From Screening Protocol RV148) 77
17.5 PREGNANCY REPORTING 77
17.6 Detailed Description of Sample Size Calculations 77
17.7 Repository Management 79

1. LIST OF ABBREVIATIONS AND DEFINITIONS OF TERMS

Abbreviation/Acronym	Definition
AA	amino acid
ACD	acid citrate dextrose
ADCC	antibody-dependent cytotoxicity
AE	adverse event
AIDS	acquired immunodeficiency syndrome
AFRIMS	Armed Forces Research Institute of Medical Sciences
ANOVA	analysis of variance
Ab	antibody
AVEG	AIDS Vaccine Evaluation Group (U.S.)
CBC	complete blood count
$CCID_{50}$	Cell culture infectious dose (50%)
CHO	Chinese hamster ovary
CID_{50}	chimpanzee infectious dose (50%)
CPM	counts per minute
CFR	Code of Federal Regulations (U.S.; Chapter 21 refers to FDA)
CRA	clinical research associate
CRF	case report form
CTL	cytotoxic T lymphocyte
DCAC	Data Coordinating and Analysis Center
DDC	Department of Disease Control (Thai MOPH)
DMU	Data Management Unit, Mahidol University
DNA	deoxyribonucleic acid
DSMB	Data and Safety Monitoring Board
EDTA	ethylene diamine tetraacetic acid
EIA	enzyme immunoassay
ELISA	enzyme-linked immunosorbent assay
ERC	Ethical Review Committee
FDA	Food and Drug Administration (Thai or U.S.)
GCP	good clinical practice
GMT	geometric mean titer
HIV and HIV-1	human immunodeficiency virus, type 1
HLA	human leukocyte antigen
HMJF	Henry M. Jackson Foundation
HSRRB	Surgeon General's Human Subjects Research Review Board
ICH	International Conference on Harmonization
IRB	Institutional Review Board
ITT	intention-to-treat
LMM	Local Medical Monitor
LPA	lymphocyte proliferation assay
LSI	lymphocyte stimulation index
MHC	major histocompatibility complex
MOPH	Ministry of Public Health, Royal Thai Government
NAb	neutralizing antibody
NAT	nucleic acid test

NIH	National Institutes of Health (U.S.)
ORP HRPO	the Office of Research Protections, Human Research Protections Office
PBMC	peripheral blood mononuclear cells
PCR	polymerase chain reaction
PPA	per-protocol analysis
RCQ	Regulatory Compliance and Quality
ROC	USMHRP Regulatory Operations Center (Rockville, MD)
RNA	ribonucleic acid
RTA	Royal Thai Army
SAE	serious adverse event
SMM	Study Medical Monitor
SOP	standard operating procedure
TAVEG	Thai AIDS Vaccine Evaluation Group
$TCID_{50}$	tissue culture infectious dose (50%)
TCLA	T cell line adapted
UNAIDS	Joint United Nations Programme on HIV/AIDS
USAMMDA	U.S. Army Medical Materiel and Development Agency
USAMRMC	U.S. Army Medical Research & Materiel Command
USMHRP	U.S. Military HIV Research Program
VE	vaccine efficacy
VSI	Vaccine Trial Senior Investigator
WB	Western blot
WHO	World Health Organization
WRAIR	Walter Reed Army Institute of Research

2. ETHICS

2.1 INSTITUTIONAL REVIEW BOARDS (IRBS)

The Principal Investigator agrees to provide the IRBs with all appropriate materials, including the protocol(s), informed consent documents, investigators' brochures, teaching and recruiting documents, and any changes made to these documents subsequent to submission. This trial will not be initiated until appropriate IRB approvals of the protocol, informed consent documents, investigators' brochures and teaching/recruiting materials have been obtained in writing by the Principal Investigator and copies have been received by the Sponsor. Appropriate reports on the progress of the study by the Principal Investigator will be made to the IRBs and the Sponsor in accordance with applicable governing regulations and in agreement with policy established by the Sponsor.

Federalwide Assurance Numbers of participating IRBs:

MOPH:	FWA00001953
RTA Med Dept:	FWA00001813
Mahidol Univ, Fac of Trop Med:	FWA 00000926
ORP HRPO:	N/A

2.2 TEST OF UNDERSTANDING (WILL BE PERFORMED IN THE SCREENING PROTOCOL, RV148)

To ensure that subjects willing to participate in the trial fully understand the informed consent process, an educational session will be given to each volunteer. This session will cover general knowledge of HIV/AIDS, the HIV vaccines to be used in this trial, and all aspects of the clinical trial including the risks/benefits. The objective of this session is to provide participants with information they need to make a fully informed decision to participate in the trial and the issues related to such a decision. Comprehension will be evaluated through a Test of Understanding (see Appendix 17.4) and will be one of the eligibility criteria for enrolling into the study. The Test of Understanding will include questions related to the vaccine and trial, to include the cause of AIDS, mode of transmission of HIV, unproven protective efficacy of vaccine, possible induction of false positivity of HIV tests, and freedom to withdraw from the trial. (Greater detail regarding this Test and its administration can be found in the screening protocol.)

2.3 INFORMED CONSENT

A properly executed, written, IRB-approved informed consent (in Thai), in compliance with the Declaration of Helsinki, guidelines of the International Conference on Harmonization (ICH) and UNAIDS, and US law 21 CFR 50, and all participating IRBs (see Section 2.1 above) shall be obtained from each subject prior to entering the subject into the trial. The investigator shall provide a copy of the IRB-approved informed consent to the subject and the signed original shall be maintained in the volunteer's record file. Attention is directed to the basic elements that are required to be incorporated into the informed consent under US Federal Regulations for Protection of Human Subjects (21 CFR 50.25[a]). Additional elements of informed consent, if appropriate, must be included in the informed consent document (21 CFR 50.25[b]).

2.4 DATA AND SAFETY MONITORING BOARD

The Data and Safety Monitoring Board (DSMB) for this vaccine trial will be supported by the Sponsor. The Subcommittee for HIV Vaccine Development and Trials of the National AIDS Commission and the Sponsor will solicit names of prospective DSMB members from the MOPH, Mahidol University, the RTA Medical Department, the manufacturers, and the WRAIR/AFRIMS/NIH. The list of proposed DSMB members will be provided for consideration and approval to the Subcommittee and Sponsor. It is anticipated that the DSMB will be both Thai and international in its membership and that it will include individuals with expertise in biostatistics, the clinical and diagnostic aspects of HIV/AIDS. An unblinded, independent statistical consultant will meet with the DSMB to answer questions about safety and will also provide the DSMB with data for the interim safety and efficacy analyses. The chairperson of the DSMB will be charged with fulfilling the responsibilities defined in the DSMB charter.

It is understood that information provided to the DSMB is confidential and that all DSMB members are expected to adhere to the DSMB rights and responsibilities as defined by the charter.

3. INVESTIGATORS AND STUDY ADMINISTRATIVE STRUCTURE

Principal Investigator:
Dr. Supachai Rerks-Ngarm
Department of Disease Control
Ministry of Public Health
Nonthaburi, Thailand
Tel: 66-2-590-3006
Fax: 66-2-965-9569

Co-Principal Investigator:
Dr. Supamit Chunsutthiwat
Department of Disease Control
Ministry of Public Health
Nonthaburi, Thailand
Tel: 66-2-590-3370
Fax: 66-2-592-8432

Senior Investigators:
LTG Sorachai Nitayaphan
Royal Thai Army Expert, AFRIMS
RTA Component, AFRIMS
315/6 Rajvithi Road
Bangkok 10400, Thailand
Tel: +66 2 354 7586
Fax: +662-354 7848

Asst. Prof. Jaranit Kaewkungwal
BIOPHIC-Center of Excellence for Biomedical and Public Health Informatics
Tel/Fax: 66-2-354-9181/9187
The 60 Anniversary of His Majesty the King's Accession to the Throne Building, 3rd -4th floor,
420/6 Rajvithi Road
Bangkok, 10400, Thailand
Tel: +662 354 9181-86
Fax: +662 354 9187

Prof. Punnee Pitisuthithum, DTM&H
Chief, Clinical Trials Section
Vaccine Trial Centre
Faculty of Tropical Medicine, Mahidol University
420/6 Rajvithi Road
Bangkok, 10400 Thailand
Tel: +662 354 9173
Fax: +662 354 9174

Directors of Field Sites (See Appendix 17.2):
Director, Rayong Field Site:
Chief, Provincial Health Office
MOPH

Director, Chon Buri Field Site:
Chief, Provincial Health Office
MOPH

Study Medical Monitor/Alternate:
COL Jeffery Berenberg
Department of Medicine
Tripler Army Medical Center
1 Jarrett White Road
Honolulu, HI 96859-5000
Tel/Fax: 1-808-433-6527/2707

Local Medical Monitor:
Dr. Vichai Satimai
Director, Regional Office # 3
DDC, MOPH
Wachiraprakarn Road
Chon Buri 20000, Thailand
Tel: 66-038-271-881
Fax: 66-038-274-862

Manufacturers:
Sanofi Pasteur
Discovery Drive
Swiftwater, PA 18370
Contact: Dr. Sanjay Gurunathan
Tel/Fax: 1-570-839-6185/0934

Global Solutions for Infectious Diseases
800 Dubuque Ave.
S. San Francisco, CA 94080
Contact: Dr. Don Francis
Tel/Fax: 1-650-228-7900/7901

Sponsor (Study IND Holder):
Kenneth A. Bertram, MD, PhD
U.S. Army Medical Research and
Materiel Command (USAMRMC)
Division of Regulatory Activities and Compliance

US Army HIV Vaccine Product Manager;
LTC Robert J. O'Connell, MD
Department of Retrovirology
USAMC-AFRIMS
Bangkok, 10400 Thailand

1430 Veterans Drive
Fort Detrick, MD 21702-9232
Tel: 301-619-0317; DSN: 343-0317

Tel: 66-2-696-2700 x 4901
Fax: 66-2-644-4824

4. BACKGROUND AND RATIONALE

Introduction

The devastating impact of the HIV/AIDS epidemic is being increasingly felt in many countries across the world and represents one of the greatest public health challenges of our time. At the end of 2001, the Joint United Nations Program on HIV/AIDS (UNAIDS) estimated that 40 million adults and children worldwide are living with HIV/AIDS. One third of these estimated infections are in those aged 15-24 while 93% are aged 15-49. Approximately 14,000 HIV infections occur each day, predominantly in the developing countries of Sub-Saharan Africa, Asia and South America. More than 20 million people have died of AIDS so far (1). The Thai Working Group on AIDS Projections estimated that as of the year 2000 nearly one million Thais had been infected and nearly 700,000 were living with HIV/AIDS. It is estimated that during the year 2000 approximately 29,000 Thais were newly infected and 55,000 progressed to AIDS. About 15,000 children per year are born to HIV positive mothers in Thailand (2). There are indications that HIV transmission between spouses is now responsible for more than half of new infections (1), a reminder that both high- and low-risk groups need to be targeted in vaccine studies in this region.

Although antiretroviral therapy has prolonged and improved the quality of life of patients, it does not lead to eradication of HIV. The severe adverse reactions resulting from these treatments have led to poor adherence and the development of resistance to drug regimens. Furthermore, the problem of uncertainty of the durability of therapeutic effects still remains, as well as the high cost that makes it inaccessible to the vast majority of the world's HIV-infected people, especially in developing countries where the HIV-1 epidemic has hit hardest (3).

The development of an effective preventive vaccine against HIV-1/AIDS therefore offers the best hope to re-enforce the control and prevention of this infection (4). One major limitation to HIV vaccine development has been the genetic variability of the virus. HIV-1 exists as multiple genetic groups and subtypes. In the United States, the vast majority of HIV-1 strains are subtype B (Group M); in Thailand, while subtype B is also circulating, the majority of new infections are with subtype E (5). [Genetically, the designation "subtype E" is incorrect. The "subtype E" virus circulating in SE Asia is a recombinant of subtypes A and E, properly designated circulating recombinant form CRF01_AE. However we use the more common "subtype E" designation throughout this document.] Data from antibody cross-reactivity studies demonstrate that binding and neutralizing antibodies from subtype B- and E-infected subjects react preferentially with viruses from the same subtype (6,7). However, both specific knowledge of immune correlates of protection and an HIV-1 animal model that is validated for predicting vaccine efficacy in humans are lacking (6,8,9). In this setting, a historically valid approach is to empirically identify and test products that elicit humoral and cellular immune responses to viral strains prevalent within the region where the candidate vaccine will be evaluated.

The Aventis Pasteur live recombinant canarypox vector vaccine ALVAC-HIV (vCP1521) under study can express several HIV antigens and induce cell-mediated immunity (10a, 10b, 49). The VaxGen gp120 B/E envelope vaccine (AIDSVAX® B/E) under study elicits significant levels of neutralizing antibodies in healthy human volunteers against subtypes B and E in the Thai population (11).

HIV Vaccine Development in Thailand

In response to the epidemic, the Royal Thai Government developed and implemented a comprehensive plan for prevention and control of HIV; preventive HIV vaccines are an integral component of this plan (12). Consequently, the National AIDS Commission of Thailand established a Subcommittee for HIV Vaccine Development and Trials with responsibility for coordinating and overseeing efforts in this area.

In 1991, the World Health Organization (WHO) selected Thailand as a site for evaluation of candidate HIV vaccines (13). Since then, the WHO (and UNAIDS) has provided consultation to the Subcommittee for HIV Vaccine Development and Trials of the National Commission for the Prevention and Control of AIDS. Through multiple partnerships and collaborations, Thailand has actively carried out its National Plan. Seven phase I/II trials of preventive HIV vaccines have been completed (14, 14a). The first (and only) phase III HIV vaccine trial outside of North America and Europe completed enrollment in Bangkok during 2000. The combination of planning, collaboration and commitment to HIV vaccine development has put Thailand in a position of international leadership concerning HIV vaccine development.

The Thai AIDS Vaccine Evaluation Group (TAVEG) is made up of clinicians and scientists from the Royal Thai Army, Mahidol and Chiang Mai Universities, the U.S. Army and recently the Department of Disease Control of the Ministry of Public Health (MOPH) (15). In collaboration with Chiron Vaccines (Emeryville, CA), the TAVEG completed two phase I/II HIV vaccine trials (16, 17). The second of these trials was an evaluation of a vaccine construct (Chiron gp120 SF2/CM235 bivalent) designed specifically for the HIV subtypes (E and B) most prevalent in Thailand. Most recently, in collaboration with Aventis Pasteur (Swiftwater, PA, USA), Chiron Vaccines and VaxGen, Inc. (Brisbane, CA), two phase I/II clinical trials employing a subtype E canarypox prime with one of three candidate envelope subunit boosts were completed in 2001. The approach defined within this protocol incorporates genes/proteins from HIV-1 viruses isolated from individuals infected with Thai subtype E. This has been possible due to the years of effort, by multiple research teams, which have resulted in the availability of Thai E immunogens for clinical evaluation as candidate HIV vaccines. Results obtained from this trial will directly benefit Thailand and other countries in SE Asia where subtype E infections are prevalent and indirectly provide the world with crucial information on the efficacy of this prime-boost strategy of HIV vaccines. This protocol proposes to test the efficacy of the prime-boost concept using candidate vaccines matched for the Thai regional subtypes of HIV-1 (prime containing subtype B and E genes; boost consisting of both B and E subunit antigens).

AIDSVAX® B/E Envelope Vaccine Candidate

A variety of HIV-1 envelope (Env) vaccine candidates have been tested in both seronegative and seropositive populations. Studies of recombinant envelope glycoprotein vaccines expressed in Chinese hamster ovary (CHO) cells have demonstrated safety and immunogenicity. In February, 2003, the results of an efficacy trial of AIDSVAX® B/B gp120 vaccine evaluated primarily among homosexual men and a small number of high-risk women in the U.S., Canada and the Netherlands showed neglible efficacy (ITT efficacy of 5.7%, CI: -17% to 24%). The efficacy of AIDSVAX® BE gp120 vaccine in another trial among injecting drug users in Bangkok, Thailand in late 2003 showed no efficacy when it was used by itself, i.e. when it was not used in combination with another vaccine.

AIDSVAX® B/E is manufactured by VaxGen, Inc. It is a mixture of two highly purified glycoproteins produced by recombinant DNA procedures by the CHO cell expression of the gp120 envelope sequences from MN (subtype B, syncytia-inducing, CXCR4-dependent virus) HIV-1 and A244 (subtype E, macrophage tropic, CCR5-dependent virus) HIV-1 to produce the B/E vaccine. The

gp120 sequences are covalently bound to a 27-amino acid sequence found in the gD protein of herpes simplex virus type 1. High-yield clones of CHO cells are selected and grown in volumes between 2,000 and 10,000 liters. Through several purification steps, including immuno-affinity chromatography, essentially pure (99.9%) recombinant gp120 is obtained for formulation.

This bivalent AIDSVAX® B/E follows the development and testing in animals and humans of monovalent AIDSVAX® rgp120 products.

Monovalent AIDSVAX®. Two chimpanzees immunized with AIDSVAX® IIIB at 0, 1 and 8 months were protected from 10 times the CID_{50}, or 40 times the $TCID_{50}$, of HIV_{IIIB} at 3 weeks post 3rd vaccination (19). Similarly, 3/3 chimpanzees vaccinated with AIDSVAX® MN and challenged with 20 CID_{50} or 748 $TCID_{50}$ of HIV_{SF2} were protected from infection (20).

Monovalent AIDSVAX® IIIB and AIDSVAX® MN vaccines (adjuvanted with alum) have been studied in over sixteen Phase I and II trials for safety and immunogenicity for both preventive and therapeutic indications in the US and in Thailand. In addition, the protein has been mixed with an experimental adjuvant, QS-21, for prophylactic vaccine studies and has been administered intradermally (without adjuvant) as a delayed-type hypersensitivity (DTH) reagent in four Phase I clinical trials.

Monovalent AIDSVAX® vaccines have been given to over 1600 individuals in Phase I and II clinical trials. This group includes over 400 HIV-uninfected individuals including infants born to HIV-1-infected mothers and adults in the U.S. and Thailand at both low and high risk for exposure to HIV-1 through sexual transmission or IV drug use. This group also includes nearly 600 HIV-infected individuals including children, pregnant women, homosexual and heterosexual adults. The objective of these studies was to evaluate the safety and immunogenicity of AIDSVAX® in different populations.

The preventive vaccine studies have shown that doses of 100-600 μg of AIDSVAX® IIIB and AIDSVAX® MN are well tolerated and immunogenic, producing antibodies that both bind to and neutralize the homologous strain of HIV-1 in almost all recipients after two or three doses. For example, in the phase I study (AVEG 006) of AIDSVAX® IIIB, 9 of 10 (90%) volunteers who received two 300 μg injections developed anti-gp120 antibodies, while 10 of 10 (100%) volunteers who received 3 injections developed anti-gp120 antibody. Nine of 10 (90%) recipients' serum after the third injection neutralized the IIIB virus.

For AIDSVAX® MN recipients of either 300 μg or 600 μg dose (pooled data from AVEG 009 and 201), 99% of volunteers developed anti-gp120 antibodies after the 2nd injection (given 1 month after the first injection) and 99% of volunteers developed anti-gp120 antibodies after the 3rd injection (given 6 months after the first injection). MN HIV-1 virus neutralizing capability was detected in 85% of these volunteers after the 2nd injection and in 99% of these volunteers after the 3rd injection. The human responses to AIDSVAX® when compared to responses in chimpanzees that were protected from HIV-1 infection, showed considerable similarities. For example, the titers of HIV-1 envelope-specific antibodies in immunized humans were comparable to those of immunized chimpanzees protected from HIV-1 infection (20, 21).

Some cross-neutralization of other strains of HIV-1 (IIIB, SF-2) has also been observed in AIDSVAX® MN recipients (20, 22). In one study (AVEG 009), groups of 12 volunteers were given three different doses of AIDSVAX® MN and one group received a combination of 300 μg AIDSVAX® IIIB and 300 μg AIDSVAX® MN. Forty-six of 48 (96%) volunteers developed neutralizing antibodies to the MN strain after three doses, and 45 of 48 (94%) volunteers developed antibodies that neutralized SF-2 HIV-1 while 30 of 48 (63%) volunteers developed antibodies that neutralized IIIB

HIV-1. Positive gp120-specific cytotoxic lymphocyte (CTL) responses were rare in recipients of AIDSVAX® MN. AIDSVAX® given intramuscularly does induce antibodies on mucous membranes (22). AIDSVAX® has induced ELISA-reactive ("false positive") results in HIV-1 antibody ELISAs performed during clinical studies. These vaccine-induced, ELISA-reactive test results were transient and were easily distinguished from true HIV-1 infection by immunoblots. There is little, if any, chance that participants in gp120 vaccine trials could be misclassified as being HIV-infected, provided a positive HIV ELISA is followed by confirmatory immunoblot (23).

Bivalent AIDSVAX® B/E and B/B. AIDSVAX® B/E was shown to be highly immunogenic in rabbits, with high binding antibody titers after the second vaccination. These antibodies appear to neutralize homologous isolates in an adapted PBMC neutralization assay (23a).

Two Phase I/II trials to determine the safety and immunogenicity of AIDSVAX® B/B and AIDSVAX® B/E have been completed in the U.S. and Thailand, respectively (24,11). One of three doses of each bivalent vaccine was given at months 0, 1, 6 and 12. In the U.S. trial, 122 volunteers were immunized with either 100, 300 or 600 μg of AIDSVAX® B/B vaccine or 300 μg of AIDSVAX® B/E vaccine. For all dose groups, the most common reactogenicity, pain or tenderness at the injection site, was reported by 74% of subjects. Six serious adverse events were reported and none were attributed to vaccine. Two volunteers terminated participation in the AIDSVAX® B/B trial; one experienced erythematous, pruritic patches on both upper extremities, while the other had moderate redness, swelling, and pain at the injection site. The immune response as measured by V2 peptide ELISA assays to the bivalent vaccine is antigen specific. One hundred percent of subjects at the 300 μg dose of AIDSVAX® B/B seroconverted to GNE8-specific V2 antibodies and 96% of subjects seroconverted to MN-specific V2 antibodies after 3 doses.

In the Thailand trial, 92 volunteers were immunized with either 100, 300 or 600 μg of AIDSVAX® B/E vaccine. For all dose groups, the most common reaction, pain and/or tenderness at the injection site, was reported by 55% of subjects. Eleven serious adverse events were reported and none were attributed to vaccine. The immune response as measured by V2 peptide ELISA assays to the bivalent vaccine is antigen-specific with 100% subjects at the 300 μg dose seroconverting to A244-specific V2 antibodies and 80% of subjects seroconverting to MN-specific V2 antibodies after 3 doses.

Phase III AIDSVAX® B/E and B/B Studies

Phase III trials of AIDSVAX® B/B in North America and Europe (25) and AIDSVAX® B/E in Bangkok (26), are assessing the efficacy of these vaccines in high-risk populations in North America/Netherlands (mostly homosexual men) and Bangkok, Thailand (intravenous drug users). The North American/European trial was completed iin late 2002 with results showing neglible efficacy overall (ITT efficacy = 5.7%, CI: -17% to 24%) with evidence suggestive of higher efficacy among women and some racial groups. The efficacy of AIDSVAX® BE gp120 vaccine in another trial among injecting drug users in Bangkok, Thailand in late 2003 showed no efficacy when it was used by itself, i.e. when it was not used in combination with another vaccine. As of February 2003, nearly 5000 trial participants have received either AIDSVAX® B/B or AIDSVAX® B/E candidate vaccines with more than 33,000 doses of the vaccines administered. An independent DSMB has reviewed VaxGen's phase III trials every six months. Each trial has had seven DSMB safety reviews and received a positive safety review on each occasion. There have been two vaccine-related SAEs in the B/B trial, both being cases of cellulitis at the injection site that resolved with treatment, and no vaccine-related

SAEs in the B/E trial. Two cases of Guillian Barre syndrome have been reported among recipients of AIDSVAX®, one with B/B in the North American phase III trial and one in the RV135 phase II trial using ALVAC-HIV prime and AIDSVAX® B/E boost. Neither were considered related to vaccine. Social harm monitoring in both phase III trials show acceptable levels (27,28).

It is noteworthy that the study design of the trial being proposed here would differ from the current phase III trial being conducted in Thailand among high-risk intravenous drug users in that volunteers would be from communities with a low-incidence of HIV infection, and most HIV transmissions would be expected to be via heterosexual routes.

Canarypox HIV (ALVAC-HIV) Vaccine Candidate

ALVAC is a live canarypox vector into which multiple HIV-1 genes have been inserted, and which has undergone extensive safety and immunogenicity studies in humans. Like vaccinia virus, canarypox can accommodate large amounts of foreign DNA in its genome, and can infect mammalian cells and cause them to produce foreign proteins. In contrast to vaccinia virus, canarypox virus is host-range restricted. Thus, a safety feature of this construct is that in mammalian cells it fails to replicate and does not produce infectious progeny virus (29,30), suggesting that canarypox recombinants will not disseminate within the immunized recipient or be transmitted to unvaccinated contacts. High doses of canarypox virus have not caused significant adverse effects in a wide variety of animals, even in profoundly immunosuppressed animals (31), suggesting that canarypox recombinants are unlikely to cause disease in human recipients after intramuscular injection.

The Aventis Pasteur ALVAC-HIV vaccine (vCP1521) is a preparation of recombinant canarypox virus expressing the products of the HIV-1 *env*, *gag,* and *protease* genes. The genes are inserted into the canarypox C6 locus under the control of the vaccinia virus H6 and I3L promoters, respectively. The gp120 *env* sequence is derived from the Thai subtype E HIV-1 92TH023 strain, while the anchoring part of gp41, *gag,* and *protease* are derived from the HIV-1 strain LAI. Co-expression of Gag and Env, and appropriate *gag* processing by protease, in a vaccinia expression system results in the formation of virus-like particles that bud from the cell membrane (32). ALVAC-infected cells may present Env and Gag proteins in a near-native conformation (33). In addition, intracellular processing of foreign HIV-1 proteins via the MHC class I pathway facilitates stimulation of cytotoxic T-lymphocytes (CTLs). Part of the rationale for use of a subtype B *gag* in ALVAC-HIV (vCP1521) product is that portions of the *gag* gene are more conserved among virus subtypes. The vCP1521 construct incorporates the most conserved viral element (*gag*) with a Thai genotype E *env*. Studies have demonstrated good expression of the clade E Env element from vCP1521-infected human cells (J. Cox, unpublished data). Studies in Thailand using vCP1521 during two phase I/II trials (RV132 and RV135) have shown autologous CTL generation against vCP1521 derived antigens. Therefore, Gag CTLs elicited by vCP1521 should react with CTL epitopes on both subtype E and B virus-infected cells. Results of an AVEG-sponsored prime-boost trial (vCP205 alone or boosted with Chiron SF2 gp120/MF59) showed that CD8+ Gag CTLs from some vaccine recipients recognized target cells infected with non-subtype B viruses, including subtype E (34). The proportion of subjects with cross-reactive CTLs remains to be determined. Similarly, some Thai volunteers vaccinated with ALVAC-HIV vCP1521 developed CTL responses which had cross-clade activity.

Extensive testing of ALVAC candidate vaccines encoding a variety of different viral products has provided no evidence of toxicity in mammals (31). In general, anti-canarypox antibody titers are stable after the second injection without further increases in antibody response with subsequent injections. Use of canarypox as the live vector also avoids the problem of vaccinia immune status, which compromises the effectiveness of vaccinia when used as a live recombinant vector (35).

Rationale for Immune Priming with a Live Vectored Vaccine (Prime-Boost Strategy)

While gp120 antigens elicit a strong humoral response, they have usually not induced an anti-HIV CD8-specific CTL response. In contrast, live recombinant vaccinia virus constructs encoding HIV-1 genes can infect mammalian cells causing them to express HIV-1 proteins (36). Recombinant canarypox constructs elicit moderate to strong CD8-restricted CTLs in human volunteers, presumably due to antigen processing via the MHC class I pathway (37-38). Although ALVAC constructs generally elicit both antibody and CTL responses, the level of antibody can be significantly boosted by administration of a soluble protein antigen. In both the guinea pig and macaque models reported above, gp160 MN/LAI-2 significantly boosted antibody responses primed with ALVAC-HIV. Thus, one approach to inducing a combined humoral and cellular immune response is priming with an ALVAC construct and boosting with the appropriate soluble protein antigen (39,40). Studies utilizing the prime-boost concept and ALVAC constructs are described below.

Previous Studies with ALVAC-HIV Constructs and Subunit HIV-1 Env Vaccines

Aventis Pasteur has utilized ALVAC vectors to express genes from rabies, measles, cytomegalovirus, Japanese encephalitis viruses and HIV. Clinical trials with these constructs have demonstrated safety and immunogenicity. In over 1800 subjects receiving ALVAC-HIV constructs, more local reactions have been observed in recipients of vaccine than placebo or control recipients; however, there were no differences observed in the occurrence of systemic reactions among these groups. Additionally, there was no difference in the rates of local and systemic reactions across the various ALVAC-HIV constructs evaluated.

Safety data were pooled from seven AIDS Vaccine Evaluation Group (AVEG) studies using ALVAC-HIV vCP205 (AVEG022, AVEG022A, AVEG029, AVEG032, AVEG034, AVEG034A, AVEG202) and one WRAIR study (RV124) (Aventis Pasteur unpublished data; End of Phase II safety package – Section 8). There were 809 subjects enrolled in the seven AVEG studies and 101 subjects enrolled in Study RV124, for a total of 910 subjects. In total, four deaths were reported, none of which were considered related to vaccination. In the AVEG studies, 170 of 809 subjects reported 233 SAEs to the NIAID Division of AIDS Regulatory Operation Center, 61 of which were considered potentially related to vaccinations. Of these 61 SAEs, 8 were definitely related, 10 were probably related and 43 were possibly related to administration of the different products. Upon further evaluation, of the 61 SAEs, 45 occurred in subjects in the vaccine treatment group, 2 in the control group and 14 in the placebo group. Of the 45 SAEs in the vaccine treatment group, 7 were definitely related, 10 probably related and 28 possibly related to vaccination. The 7 definitely vaccine-related SAEs included 4 episodes of injection site reaction, 2 episodes of vasovagal reaction and 1 episode of a "cell-mediated allergic reaction". None of these 7 definitely vaccine-related SAEs resulted in death or a life-threatening condition. In study RV124, there were 9 subjects reporting 9 SAEs; none were considered related to vaccination. Generally, ALVAC-HIV-associated reactogenicity events have been mild and transient in nature. Aside from local reactogenicity events, there have been no observed patterns of excess adverse events attributable to AVAC-HIV vaccination.

Several phase I and II trials (Table 1) of ALVAC-HIV candidate vaccines alone or boosted with soluble protein have been conducted, or are ongoing (35,40,42,46-52). The six completed and fully analyzed studies have demonstrated that the HIV vaccines tested appear to be well tolerated, safe and immunogenic.

Table 1. Phase I/II trials of ALVAC-HIV test vaccines with or without protein boost

Country	4.1 TRIAL	No. of subjects	ALVAC-HIV Construct	Boost[1]	Status
France	VAC 01	20	vCP125	MN/LAI gp160	Completed
USA	AVEG 012	131	vCP125	SF2 gp120	Completed
France	VAC 03	25	vCP205	p24-V3 peptide	Completed
France	VAC 07	20	vCP300		Completed
USA	AVEG 022/22A	186	vCP205	SF2 gp120	Completed
USA	AVEG 026	119	vCP300	SF2 gp120	Completed
USA	AVEG 027	84	vCP205		Completed
USA	AVEG 029	22	vCP205	SF2 gp120	Completed
USA	AVEG 033	28	vCP205	None	Completed
USA	AVEG 202 HIVNET 014	420	vCP205	SF2 gp120	Completed
Uganda	HIVNET 007	40	vCP205	none	Completed
USA	RV124	92	vCP205	gp160 MN/LAI-2	Completed
Thailand	RV132	130	vCP1521	Chiron gp120 B/E or Aventis gp160 (E)	Completed
Thailand	RV135	125	vCP1521	VaxGen, Inc. gp120 B/E	Completed
USA	AVEG 034	18<br>32<br>32	vCP205,<br>vCP1433,<br>vCP1452	gp160 MN//LAI-2	Completed

1. Not all subjects in each trial received the protein boost.

vCP125 Prime-Boost Studies

The vCP125 vector containing gp160 MN has been tested in France (ANRS VAC 01) and in the US (AVEG 012). In ANRS VAC 01 study, 20 volunteers received vCP125 (10^6 $TCID_{50}$), which expresses the MN gp160 gene only, boosted with MN/LAI rgp160 (40). Greater than 90% of the subjects developed virus neutralizing antibodies and 40% developed detectable CTL. AVEG 012 tested vCP125 at two doses (10^6 and 10^7 $TCID_{50}$), with or without SF2 gp120, in 131 volunteers. In subjects that received vCP125 (10^7 $TCID_{50}$) only, 22% developed CTL and 50% developed anti-MN neutralizing antibodies. In subjects who received vCP125 boosted with SF2 gp120, almost all developed neutralizing antibodies and 37% developed CTL (35). In addition, neutralizing activity against the syncytia-inducing (SI) clade B primary isolate BZ167 (that was closely related genetically to the HIV-1 SF-2 and MN strains from which the vaccines were derived) was detected by two laboratories, using a PHA blast assay and a resting cell assay, in post-immunization sera of 6 of 9 recipients of ALVAC-gp160 and rgp120, in 2 of 7 rgp120 recipients, but in none of 5 recipients of ALVAC-gp160 alone (35, 41). Neutralizing activity against other SI or NSI HIV-1 strains was not detected by PHA-blast assays. However, neutralizing activity against three additional SI clade B primary isolates (SF-2, SF1-3, and SF-33) was detected in post-immunization sera from some recipients of ALVAC-gp160 and rgp120 but not in recipients of ALVAC-gp160 alone (44,45). Finally, the sequential immunization with ALVAC-gp160 and rgp120 resulted in more frequent and higher levels of HIV-1 lymphoproliferative activity to Env proteins, ADCC activity to HIV-1 MN and SF-2 strains, and CD4 blocking antibodies than did recipients of ALVAC-gp160 alone (35).

vCP205 Prime-Boost Studies

The recombinant canarypox virus ALVAC-HIV vCP205 expresses the products of multiple HIV-1 genes as follows: the *gag* gene expressing the Gag p55 protein of the HIV-1 LAI strain, the protease portion of the *pol* gene, expressing the p15 protein of the HIV-1 LAI strain, a part of the *env* gene expressing gp120 of the HIV-1 MN strain, and the anchoring transmembrane region of gp41 glycoprotein of the HIV-1 LAI strain.

The administration of ALVAC-HIV vCP205 has induced cellular and humoral responses in animals. This vaccine has the advantage of including structural gene products (Gag, Pol) other than Env. Moreover, *in vitro* transfection of the vector produces immunogenic virus-like particles in cells (32-34). In mice, this vaccine induced a cytotoxic response against target cells with the V3 peptide on their surface, as well as a lymphoproliferative response to gp160. In guinea pigs, a humoral response was induced against gp160 MN/LAI, a V3 MN peptide, and the p24 and p18 proteins of HIV-1. In macaques, humoral responses against Env and Gag proteins and neutralizing activity against the HIV-1 MN isolate were demonstrated. Further, ALVAC-HIV vCP205 has been shown in vitro to stimulate the expansion of CD8+ Env-/Gag-specific CTL precursors present in PBMCs derived from HIV seropositive individuals.

Twenty-five HIV-negative volunteers in France (ANRS VAC 03) received $10^{5.8}$ $TCID_{50}$ ALVAC-HIV vCP205 at 0, 1, 3 and 6 months or at 0 and 1 months followed by an HIV candidate peptide vaccine, CLTB-36, at 3 and 6 months (42). The vCP205 was well tolerated as was the CLTB-36. The vCP205 alone induced binding antibodies (ELISA) to rgp160 MN/LAI and to a V3 MN peptide as well as lymphoproliferation one month after the third injection and anti-Env and anti-Gag CTL activity was detected in some of the volunteers.

In AVEG Protocol 022 volunteers received $10^{5.8}$ $TCID_{50}$ vCP205 with or without a boost of HIV-1 SF-2 rgp120 (46). Following immunizations on a 0, 1, 3 and 6 month schedule, 26 of 42 subjects who received vCP205 demonstrated in vitro $CD8^+$ T cell responses, versus 3 of 17 who received the control ALVAC-rabies or gp120 vaccine only (P = 0.0003); 15 of these 26 demonstrated a CD8+ CTL response on two occasions post-vaccination. The frequency of CD8+ CTL response to HIV antigens was similar between vaccinia-naive and vaccinia-immune persons. Rgp120 immunization did not increase the CD8+ CTL response to HIV type 1 Env proteins, but rgp120 boosting markedly enhanced the titer and frequency of neutralizing antibodies to the MN strain of HIV. Overall, the combination of vCP205 and recombinant gp120 resulted in neutralizing antibodies in 93% and CD8+ T cell responses in 62% of subjects. It is of interest to note that two weeks post-fourth immunization, 72% of the volunteers had a positive ELISA (Abbott) and 40% had Env band reactive Western blots.

In this and all subsequent AVEG studies, neutralizing antibodies were measured by the vital dye neutralizing antibody assay. Seventy percent of the volunteers who received four immunizations of vCP205 developed HIV-1 MN neutralizing antibodies whereas, 52% of the volunteers receiving three immunizations of vCP205 developed HIV-1 MN neutralizing antibodies. Giving SF2 rgp120 simultaneously or after vCP205 increased HIV-1 MN and HIV-1 SF2 neutralizing antibodies positivity to 80-100% of the volunteers and augmented HIV-1 MN neutralization titers.

In AVEG 022A, volunteers received 10^7 $TCID_{50}$ of vCP205. One-half of the volunteers received five immunizations at 0, 1, 6, 9 and 12 months and the other half received 6 immunizations at 0, 1, 3, 6, 9 and 12 months. Twenty volunteers received only vCP205. Forty-two volunteers were immunized simultaneously with both vCP205 and SF2 rgp120. Another 22 volunteers received either 3 or 4 immunizations with vCP205, followed by two SF2 rgp120 immunizations. Finally, 42 volunteers received 2 immunizations with vCP205 followed by 3 or 4 immunizations of vCP205 and SF2 rgp120 given simultaneously. The 24 control volunteers received ALVAC-rabies instead of vCP205 or placebo (the MF59 adjuvant and saline vehicle for the SF2 rgp120) instead of SF-2 rgp120.

Anti-Env and/or anti-Gag CTL activity was detected in 58% of the vaccinees on at least one occasion; 30% had CD8+ CTL more than once. CD8+ CTL were detected similarly in low-risk and high-risk volunteers (61% and 56%, respectively). Two weeks after the 6-month vaccination, volunteers on the 0, 1, 3, 6-month schedule demonstrated significantly higher CD8+ CTL responses than those on the 0, 1, 6-month schedule (23/51, 45% vs. 12/48, 25%), suggesting that additional vaccination at 3 months may increase the induction of CTL responses. HIV neutralizing antibody results were consistent with previous trials of vCP205.

AVEG Protocol 029 was designed to examine response and tolerance to multiple immunizations with ALVAC vCP205 given over 3 months (47). Thirty-four volunteers received four immunizations with the 10^7 $TCID_{50}$ of vCP205 (22 volunteers) or the ALVAC-rabies control (12 volunteers) at 0, 7, 14, and 21 days followed by an HIV-1 SF-2 rgp120 immunization or placebo at 28 and 84 days. Two weeks after the second rgp120 immunization, 100% of the volunteers had HIV-1 MN neutralizing antibodies and 68% expressed anti-HIV CTL activity at some time point.

A US phase II trial (AVEG 202/HIVNET 014) tested vCP205 with and without an SF-2 rgp120 boost given at 0, 1, 3, and 6 months in 435 subjects, of whom 60 were of lower risk and 375 were at higher risk for acquisition of HIV-1 infection (48). The vaccines were generally well tolerated in these groups. More than 90% of subjects who received the combined regimen developed neutralizing antibody responses against homologous, T-cell line adapted virus. Approximately one-third of those who received vCP205-containing regimens developed anti-HIV CTL responses. There appeared to be no significant differences in immune responses among the higher and lower risk groups in the study.

In RV124, HIV seronegative volunteers received vCP205 as well as oligomeric gp160 MN/LAI-2 in polyphosphazene (49). Cumulative CD8+ CTL generation was 37% (95% CI, 23-52%) in the ALVAC-HIV groups. Neutralizing antibodies were detected to the autologous TCLA MN virus in 18 of 20 (90%) recipients of a sequential vCP205 prime + 50 mcg or 100 mcg boosts of gp160MN/LAI-2. In roughly 10% of vaccinees, primary isolate neutralizing antibodies have been detected at low levels.

vCP300 Prime-Boost Studies

A third canarypox-HIV recombinant, ALVAC vCP300, which expresses gp120 (MN), the transmembrane portion of gp41 (LAI), as well as HIV-1 LAI *gag*, *protease* and multiple CTL epitopes of *nef* and *pol* gene products, was tested in protocols ANRS VAC 07 and AVEG 026 (50). The inclusion of *nef* and *pol* epitopes in this vaccine was intended to cover more HLA types in a given population and possibly limit the emergence of CTL escape mutants shown to be transmitted in natural infection, and avoid clonal exhaustion of monoclonally expanded HIV-specific CTL clones. Some volunteers received HIV-1 SF2 rgp120 simultaneously with the vCP300 or following vCP300 on either a 0, 1, 3, 6 or a 0, 1, 6, 9 month schedule. Of the 140 volunteers in AVEG 026, 119 received vCP300 and of these, 85 also received rgp120. Data confirm the previously observed superiority of binding and neutralizing antibodies in volunteers receiving SF2 rgp120 as part of their vaccination regimen. Results indicate that CTL activity against HIV Env and Gag proteins was induced, while only limited Pol- and Nef-directed activity was detected. When Env and Gag CTL activities were analyzed at 6 and 12 months, there were no clear differences for each immunization regimen. At 12 months, CTL activity against Gag or Env was detected in 32% and 22%, respectively, of volunteers who received vCP300. The highest CTL rates (40%-63%) were detected at 12 months in volunteers immunized with four doses of vCP300 along with two or four doses of rgp120.

vCP1433 and vCP1452 Prime-Boost Studies

Complex ALVAC vectors designed to provide an increased and longer gene expression may result in more durable CD8+ CTL response against one or several targets. Two new recombinant canarypox virus constructs were developed: ALVAC-HIV (vCP1433) and ALVAC-HIV (vCP1452). Both vCP1433 and vCP1452 express *gag*, *protease*, *nef*, and *pol* genes and a part of the *env* gene expressing the gp120 and anchoring region of gp41. The ALVAC vCP1452 vector was modified by the insertion of two vaccinia virus coding sequences (E3L and K3L) to enhance the expression efficiency in ALVAC-infected human cells. vCP1433 and vCP1452 were evaluated in comparison with vCP205 in AVEG 034.

vCP1521 Prime-Boost Studies

Phase I/II trials (RV132 and RV135) of vaccine combinations that use vCP1521 as the prime have been carried out by the TAVEG in Thailand during 1999-2001 (51,52). Immunogenicity data from RV132 and RV135 suggest that the vCP1521 ALVAC-HIV vector, in combination with a variety of gp120 or gp160 subunit envelope boosts, induces immune responses that are consistent with predefined immunogenicity targets (LPA > 60% responders, CTL > 30% responders, and NAb > 70% responders) for advancement to a phase III trial.

In RV132 (vCP1521 + gp160 TH023 or gp120 CM235/SF2), 93% of gp160 TH023 vaccine recipients and 68% of gp120 CM235 vaccine recipients developed CM235-specific lymphoproliferative responses 2 weeks post-4th vaccination (a background rate of 3-4% positive responses was found at baseline and post-vaccination). 84% of gp160 TH023 vaccine recipients and 75% of gp120 SF2 vaccine recipients developed SF2-specific lymphoproliferative responses 2 weeks post-4th vaccination (background positivity was 5-10%). 87% of gp160 TH023 vaccine recipients and 55% of gp120 CM235 vaccine recipients developed TH023-specific lymphoproliferative responses 2 weeks post-4th vaccination (background positivity was 2-3%). In RV135, 58 and 67% of persons receiving the prime plus high or low dose boost developed A244-specific proliferative responses 2 weeks post-4th vaccination (background positivity was 6-7%). Proliferative responses to MN were problematic because of high levels of background proliferation to the MN protein. 62 and 60% of

persons receiving the prime plus high or low dose boost had positive proliferative responses; background positivity was 11-24%.

In RV132, NAb to the TCLA subtype E strain NPO3 was found in 84% and 89% of recipients of vCP1521 + gp160 TH023 or gp120 CM235/SF2, respectively. Neutralization of the TCLA SF2 strain was seen in 27% and 61% of recipients of vCP1521 + gp160 TH023 or gp120 CM235/SF2, respectively. Neutralization of the adapted CM244 primary strain was seen in 89% and 95% of recipients of vCP1521 + gp160 TH023 or gp120 CM235/SF2, respectively. 96% and 100% of vCP1521 + gp160 TH023 or vCP1521 + gp120 CM235/SF2 (respectively) prime-boost recipients had NAb against subtype E-adapted HIV-1. In RV135, NAb to the TCLA subtype E strain NPO3 was found in 31% of recipients of vCP1521 + 300μg/300μg AIDSVAX® B/E. Neutralization of the adapted CM244 primary strain was seen in 64%. 71% of prime-boost recipients had NAb against subtype E-adapted HIV-1. No placebo recipient in either RV132 or RV135 developed NAb against the HIV-1 subtype E strains tested.

In RV132, bulk HIV-specific CTL responses were detected in 16% of volunteers receiving vCP1521 + gp160 TH023 and 25% of those receiving vCP1521 + gp120 CM235/SF2. HIV-specific CD8+ CTLs were detected in 11% and 25% of prime-boost recipients in the gp160 TH023 + gp120 CM235/SF2 arms of RV132, respectively. Among placebo recipients, bulk and CD8+ HIV-specific CTLs were found in 10%.

In RV135, bulk HIV-specific CTL responses were detected in 23% of recipients of the vCP1521 + 100 mcg/100 mcg AIDSVAX B/E regimen and 28% among recipients of the higher dose of AIDSVAX B/E prime-boost regiment. Bulk HIV-specific responses were found in 7% of placebo recipients. HIV-specific CD8+ CTLs were identified in 23% of vaccinees receiving either boost. No placebo recipient had CD8+ HIV-specific CTL responses. If all ALVAC recipients in RV135 are considered in aggregate, 22 of 91 (24%) had CD8+ HIV-specific CTLs.

Vaccines were well tolerated by all volunteers. There were 7 SAEs in RV132 and 7 in RV135; all were assessed as not associated with vaccine.

Summary of ALVAC-HIV Vaccines

Available data have shown to date that ALVAC-HIV candidate vaccines are well tolerated, safe and induce HIV-specific CTL responses in 30-60% of volunteers, depending on the dose and gene products expressed. When given alone, ALVAC-HIV induces limited neutralizing antibody responses in many volunteers after 3 or 4 immunizations; the neutralizing antibody titers are modest. Prime-boost regimens with ALVAC-HIV and SF2 gp120/MF59 generate detectable neutralizing antibody against laboratory-adapted HIV-1 strains in almost all vaccine recipients, and the levels are significantly higher than with ALVAC-HIV alone. While neutralizing antibodies do not appear to be active across HIV-1 subtypes, CTL activity can be cross-clade reactive in some subjects.

Rationale for the Immunization Schedule

In previous studies, different immunization regimens have been used, with vaccination schedules as long as 12 months. To determine whether a short immunization schedule could be used to elicit immune responses, the immunogenicity data from volunteers who received 3 or 4 immunizations with vCP205 or vCP300 and 2 to 4 immunizations with SF2 gp120 within a 6-month period in AVEG phase I trials, Protocols 022A, 026 and 029 were compared.

Table 2. Summary of CTL and Neut. Ab Results in vCP205 and vCP300 Prime Boost Studies

		Cumulative CD8+ CTL (+/#)				
**Immunization Schedule**	**Prime-Boost Protocol***	**Env**	**Gag**	**Pol**	**Any**	**Neut Ab (MN)**
**0,1,3,6 mo**						+/# GMT**
**vCP205**	A A AS AS	6/20	9/20	7/20	10/20	14/14 323
**vCP205**	AS AS AS AS	11/20	10/20	6/19	13/20	15/15 54
**vCP300**	A A S S	2/17	2/17	2/17	4/17	17/17 193
**vCP300**	A A AS AS	1/14	3/14	0/14	4/14	14/15 372
**0,1,6 mo**						
**vCP205**	AS AS – AS	4/20	4/19	2/28	6/20	15/16 144
**vCP300**	AS AS – AS	2/15	4/16	2/16	7/16	15/16 88
**0,1,2,3,4,12 wk**						
**vCP205**	A A A A S S	8/22	2/16	7/20	15/22	21/21 323

*A = ALVAC
S = gp120 SF2, 50 μg in MF59 (Chiron Vaccines)
** GMT = Geometric Mean Titer

As shown in Table 2, the frequency of Env or Gag CD8+ CTLs varied, depending in part on the number of time points studied. The administration of vCP205 four times and SF2 rgp120 twice in AVEG Protocols 022A and 029 induced Env and/or Gag-specific CD8+ CTL responses more often, as well as higher geometric mean titers of HIV-1 MN-specific neutralizing antibodies, than the other regimens. Immunizations at 0, 1, 3 and 6 months require four visits, which is preferable for recruitment and logistical reasons to the six visits in AVEG 029. For these reasons, the immunization regimen to be employed in this study will be to administer the ALVAC vector at 0, 1, 3 and 6 months and the AIDSVAX® B/E subunit vaccine at 3 and 6 months.

Vaccine and Placebo Preparations

ALVAC-HIV (vCP1521) is a recombinant canarypox vector vaccine that was developed by Virogenetics Corporation (Troy, NY) and manufactured by Aventis Pasteur. The recombinant canarypox has been genetically engineered to express subtype E HIV-1 gp120 (92TH023) linked to the transmembrane anchoring portion of gp41 (strain LAI), and HIV-1 *gag* and *protease* (LAI strain). It is grown in chick embryo fibroblasts derived from pathogen-free chicken eggs. The preparation is lyophilized and stored between +2ºC to +8ºC at the clinical site. ALVAC-HIV (vCP1521) is formulated at a dose of $>10^6$ $CCID_{50}$. Prior to administration, the vaccine preparation is reconstituted with 1 mL of 0.4% saline (NaCl).

ALVAC Placebo (Aventis Pasteur) is supplied as a sterile, lyophilized product that consists of a mixture of virus stabilizer, and freeze drying medium. The diluent supplied for reconstitution of ALVAC-Placebo consists of sterile 0.4% NaCl, which is adjusted to a pH of 5 to 7 and conforms to established requirements for sterility, safety, and pyrogen testing. The volume preparation is 1 mL.

AIDSVAX® B/E, produced by VaxGen, Inc., is a bivalent HIV-1 gp120 envelope glycoprotein vaccine containing a clade B envelope from the HIV-1 strain MN and a clade E envelope from the Thai HIV-1 strain A244. The recombinant gp120s are produced in a genetically engineered CHO cell line, and subsequently purified by standard techniques including immunoaffinity chromatography. The gp120s have an apparent molecular mass of 120 kD, roughly 50% of which is due to glycosylation.

The sequences representing aa 12-485 (MN) and 12-484 (A244) are preceded by a 27 aa sequence derived from the gD protein of herpes simplex virus type 1. The envelope glycoproteins of MN and A244 are coformulated and adsorbed to aluminum hydroxide (alum) gel. AIDSVAX® B/E vaccine is supplied in single-use vials containing 300 μg/mL MN rgp120/HIV-1 antigen and 300 μg/mL A244 rgp120/HIV-1 antigen on alum adjuvant (600 μg/mL alum adjuvant) per 1 mL of vaccine formulation. Each 3 mL vial has a nominal content of 1 mL and is intended for a single vaccination.

AIDSVAX Placebo (VaxGen, Inc.) is 600 μg alum adjuvant in a volume of 1 mL.

5. OBJECTIVES

5.1 PRIMARY OBJECTIVES

- To determine whether immunizations with an integrated combination of ALVAC-HIV (vCP1521) boosted by AIDSVAX® gp120 B/E prevent HIV infection in healthy Thai volunteers.
- To determine whether immunization with this vaccine combination results in reduced HIV viral load among those acquiring HIV-1 infection, comparing vaccine recipients to placebo recipients.

5.2 SECONDARY OBJECTIVES

- To determine whether immunization with this vaccine combination results in an increased CD4 count measured among those acquiring HIV-1 infection, comparing vaccine recipients to placebo recipients.
- To confirm the safety of this vaccine combination in Thai volunteers.
- To evaluate whether participation in this HIV vaccine trial is associated with behavior change that may increase the risk of HIV infection.

6. INVESTIGATIONAL PLAN

6.1 STUDY DESIGN AND PLAN

This will be a community-based, randomized (vaccine:placebo = 1:1), multicenter, double-blind, placebo-controlled clinical trial. Screening of potential volunteers will be carried out under a separate protocol entitled "Screening and evaluation of potential volunteers for a trial in Thailand of a candidate preventive HIV vaccine" (RV148). Eligible volunteers will be enrolled over approximately two years. The statistical assumptions of the study will require that 16,000 persons enroll into the study (see Section 6.9). For purposes of sample size calculations the dropout rate was assumed to be 5% per 6-month period, including the vaccination phase. If the rate of dropout during immunization exceeds 5%, additional subjects will be recruited and enrolled to achieve 15,200 volunteers completing the 24-week vaccination phase. If it becomes obvious that 15,200 volunteers will not complete the vaccine phase in a timely manner, after 16,000 volunteers have been enrolled an instruction will be sent out to the sites to stop screening. Those already screened will be allowed to enroll so slightly more than 16,000 will finally be enrolled. The concern about the sample size will be balanced by increased efforts in the tracking and retention of those volunteers who have completed the vaccine phase. Vaccinations for each individual will occur over a 24-week period (0, 4, 12, 24

weeks). Women will be tested for pregnancy and pregnant volunteers will not be vaccinated. The volunteers will be followed with HIV testing every 6 months for 3 years after immunization. Blood will be collected for plasma (for diagnostics and HIV-specific antibodies) at 0, 24 and 26 weeks, and every 6 months during the follow-up phase. The blood collection at 0, 12 and 42 (and potentially 6) months will also be used for cryopreservation and archiving of PBMCs (for HIV-specific cellular immune responses). At week 24 and at each six-month follow-up visit, volunteers will have HIV testing, preceded by pretest counseling and followed (approximately 2-3 weeks later) by post-test counseling. Assessment of HIV risk behavior will be given at baseline, week 26 and at each 6-month follow-up visit. Education on risk behavior reduction will be given at each vaccination visit and at each post-test counseling visit.

Vaccine Trial Design

		Weeks			
Group	Number	0	4	12	24
I	8,000	ALVAC Placebo	ALVAC Placebo	ALVAC Placebo + AIDSVAX Placebo	ALVAC Placebo + AIDSVAX Placebo
II	8,000	ALVAC-HIV	ALVAC-HIV	ALVAC-HIV + AIDSVAX® B/E	ALVAC + AIDSVAX® B/E

6.2 RATIONALE FOR THE STUDY ARMS

The use of a placebo group is required to allow determination of vaccine safety and efficacy. There is no HIV vaccine proven to be efficacious and thus an equivalency trial is not appropriate.

It is noteworthy that two phase III efficacy trials have been conducted with AIDSVAX , one with the study vaccine AIDSVAX® B/E and the other with a similar product (AIDSVAX® B/B'). Prior to the completion of these trials, it was decided that if those trials show no efficacy, this study design remains justified because neutralizing antibodies induced by the prime-boost combination may differ in specificity than those induced by AIDSVAX® B/E alone, or may be protective when acting jointly with HIV-specific CTLs. Also, non-efficacy in the setting of an intravenous virus inoculum (in the case of the AIDSVAX® trial in Thailand) may not predict vaccine efficacy against sexual (transmucosal) transmission. Similarly, efficacy in the setting of intravenous transmission may not predict efficacy in the setting of transmucosal infection. If either phase III trial with AIDSVAX® shows efficacy, the data would be carefully evaluated by the investigators and sponsor in conjunction with regulatory, governmental agencies and ethical review boards in regard to the design and conduct of this phase III trial.

6.3 SELECTION OF STUDY POPULATIONS

The total study population consists of 16,000 (target number is 15,200 volunteers at completion of immunization) healthy Thai HIV-uninfected adults screened through a separate protocol (see Section 6.1 above). Subjects will be 18 through 30 years of age (inclusive) and HIV-uninfected (based on HIV-specific serology). Women will agree to practice effective contraception with informed consent during the six-month immunization period and the 3 months following the fourth vaccination, and will be tested for pregnancy prior to each vaccination. Individuals will be enrolled

during an estimated 2-year period (longer if necessary). This study population will be recruited from the provinces and districts listed in the Appendix (Section 17.2), and may be expanded throughout the two provinces if required to meet enrollment targets.

6.3.1 Inclusion Criteria

Volunteers will have been pre-screened as participants of a separate protocol. This will include questions regarding pregnancy and breast-feeding, serologic testing for HIV infection and passing of a Test of Understanding.

To enroll in this protocol, all of the following criteria must be met by the potential volunteer:

1. Possession of the 13-digit Thai National ID card.

2. 18-30 years of age (inclusive), male or female.

3. For women, a negative urine pregnancy test on the day of enrollment, as well as assurance that adequate birth control measures will be applied during the course of the injections and the three months after the last injection.

4. Absence of systemic disease or immunodeficiency as determined by medical history and directed physical examination.

5. Negative serology for HIV-1 infection within 45 days prior to enrollment.

6. Availability and commitment for 3.5 years of participation.

7. Able to understand the study (shown by receiving a passing score on the Test of Understanding administered under the screening protocol) and give written informed consent.

8. Enrollment in and referral from screening protocol, RV148**.**

6.3.2 Exclusion Criteria

Individuals will not be enrolled into the study if they meet any of the following criteria.

1. Previous participation in any HIV vaccine trial (unless the volunteer can provide documentation that he/she received placebo).

2. Active tuberculosis, other systemic disease process, or immunodeficiency as detected by medical history and directed physical examination that would, in the opinion of the investigator, impede compliance with study requirements or complicate the interpretation of adverse events.

3. Any significant finding that in the opinion of the investigator would increase the risk of having an adverse outcome from participating in this study or might interfere with the volunteer’s ability to successfully complete the study.

4. Occupational or other responsibilities that would prevent completion of 3.5 years of participation in the study.

5. History of anaphylaxis or other serious adverse reactions to vaccines, or allergies or reactions likely to be exacerbated by any component of the vaccine or placebo, including egg products and neomycin.

6. Women breast-feeding or pregnant (positive pregnancy test) or planning to become pregnant during the 9-month window between study enrollment and 3-months after the last vaccination visit.

7. Study site employees who are involved in the protocol and may have direct access to trial-related data.

8. Chronic use of therapies which may modify immune response, such as IV immune globulin and systemic corticosteroids (in doses of ≥ 20 mg prednisone equivalent for periods exceeding 10 days), and use of experimental drugs or vaccines.

9. Receipt of a non-HIV vaccine or immune globulins within 14 days.

6.3.3 Discontinuation of Subjects from Trial or Assessment

A subject may withdraw his/her consent to participate in the study at any time without prejudice. Additionally, the investigator may withdraw a subject if, in his/her clinical judgment, it is in the best interest of the subject, or if the subject cannot comply with the protocol (see also Section 6.4.5). If pregnancy or HIV infection is diagnosed, no further vaccinations will be given and procedures will be followed as in section 17.5 and 6.5, respectively. Wherever possible, the tests and evaluations listed for Visits 19 and 20 should be carried out if the subject refuses follow-up according to the protocol visit schedule. The Sponsor should be notified of all study withdrawals (as per SOP).

If a subject misses a vaccination visit (i.e., Visits 1, 3, 5 or 7), no further vaccinations will be performed, but the subject will continue to be followed according to the protocol visit schedule. A missed visit is defined according to the time windows listed by visit in section 6.6. With the approval of the Sponsor or his/her designee, vaccination outside the window prescribed and continuation in the trial will be allowed in exceptional situations (as per SOP). If a subject does not complete the immunization schedule secondary to an adverse event (including SAEs) or toxicity, he or she will continue to be followed according to the protocol visit schedule for safety, and, at a minimum, until the adverse event/toxicity is resolved and/or chronicity is established. If a volunteer has not returned to the clinical site for more than a year, the volunteer will be re-consented in order to document the volunteer's willingness to continue in the trial. A genuine effort, utilizing phone, mail and home visits (if volunteer has given permission), will be made to determine the reason(s) why a subject fails to return for the necessary visits. Attempts to contact volunteer will be documented. Under certain circumstances it may be difficult or impossible for a study volunteer to return to the Clinical site for a follow up visit. For these volunteers, any follow- up visits, including those that include a blood draw, may be done in a location other than the clinical site (with the volunteers' prior agreement). For subjects who become pregnant before completing the vaccine series, no further vaccinations will be given regardless of outcome. The subject will be followed for all remaining scheduled visits

according to the SOP for safety evaluation (see Appendix 17.5). The site will maintain contact with pregnant subjects to obtain pregnancy outcome information.

6.3.3.1 Prisoners: Volunteers who become prisoners will be allowed to continue to participate in the vaccine trial. Volunteers who become prisoners are considered a "vulnerable population" and are entitled to special management and consideration. The risks and the benefits to the volunteers who become prisoners and continue in the trial are presented in section 7.1. Volunteers who become prisoners will be managed specifically as follows:

Consenting: Volunteers who become prisoners and desire to continue in the vaccine trial need to document their willingness to continue in the trial. To do this they will re-sign the original consent form, in addition they will sign an addendum to the consent specifically for prisoners which clearly outlines the potential risks to a volunteer who has become a prisoner if they continue in the vaccine trial.

Confidentiality: The potential risk to a volunteer who becomes a prisoner relates to the interaction of the volunteer with the prison authorities and with other prisoners. If other personnel (guards, prison authorities, or other prisoners) in the prison are aware that the volunteer is participating in the vaccine trial they may treat the volunteer differently. They may discriminate against the volunteer and they may abuse the volunteer (either verbally or physically). In previous trials of HIV vaccines in Thailand (the BMA VAX 03) this was not a problem. Every effort will be made to preserve the volunteers' confidentiality about participating in the vaccine trial. Visits will be scheduled through the warden or the prison physician and visits will be accomplished in a private setting in the prison. The potential risk will be clearly explained to the volunteer.

Compensation: (See section 7.2). Volunteers who become prisoners and agree to continue in the vaccine trail will receive compensation for their participation. The money will be added to the prisoner account at the prison, in the same manner that all monies earned by prisoners is handled. The money will compensate them for the additional potential risks that they may face.

Clinical Care: Volunteers who are infected and who are prisoners will be entitled to the same level of care as volunteers who are not incarcerated. They will have CD4+ and viral load determinations to guide the initiation of treatment. They will not be recruited for RV-152 (Break Thru Protocol) while they are in prison. After they are released they will be offered the opportunity to enroll in RV-152.

6.4 VACCINES AND PLACEBOS AND THEIR ADMINISTRATION

6.4.1 Summary of Vaccines and Placebos

1 Aventis Pasteur ALVAC-HIV (vCP1521), $>10^6$ $CCID_{50}$/1 mL dose
•Recombinant canarypox that expresses subtype E (*env*) and B HIV genes (*env, gag, pro*) supplied as lyophilized product, reconstituted in 0.4% NaCl and given as a 1 mL intramuscular injection into the left deltoid muscle.

2. ALVAC Placebo
•ALVAC carrier, supplied as a lyophilized product, without virus, given as a 1 mL intramuscular injection into the left deltoid muscle.

3. AIDSVAX® B/E
•Bivalent HIV gp120 glycoprotein vaccine with subtype B (MN) and subtype E (A244) coformulated and administered in aluminum hydroxide gel at a combined dose of 600 μg (300 μg of each antigen) in 1.2 mL and given as a 1 mL intramuscular injection into the right deltoid muscle.

4. AIDSVAX Placebo
•aluminum hydroxide adjuvant, 1.2 ml per vial, given as a 1 ml intramuscular injection into the right deltoid muscle.

6.4.2 Identity of Investigational Products

A label is applied on the syringe of ALVAC-HIV or its Placebo, and it must mention:

ALVAC-HIV
RV144
Study #
Vaccination # <u>L Arm</u>
Date: Time:
Manufactured by Aventis Pasteur
Use within 2 hours
Investigational Use Only

A label is applied on the syringe of AIDSVAX® B/E or its Placebo, and it must mention:

AIDSVAX® B/E
RV144
Study #
Vaccination # <u>R Arm</u>
Date: Time:
Manufactured by VaxGen, Inc.
Use within 2 hours
Investigational Use Only

6.4.2.1 Vaccine Supplies

Component	Amount Per Vial
**ALVAC-HIV (vCP1521)**	>10^6 $CCID_{50}$
10 mM Tris HCl, pH 9	0.25 ml
lactoglutamate	0.25 ml
Freeze–drying medium according to formula 65-1-2	0.50 ml
Total volume per vial	1.00 ml
Storage: Store refrigerated at 2ºC to 8ºC	
Syringe containing 1.0 ml of 0.4% NaCl	
**ALVAC Placebo**	
10 mM Tris HCl, pH 9	0.25 ml
lactoglutamate	0.25 ml
Freeze drying medium according to formula 65-1-2	0.50 ml
Total volume per vial	1.00 ml
Storage: Store refrigerated at 2ºC to 8ºC	
Syringe containing 1.0 ml of 0.4% NaCl	
**AIDSVAX® B/E gp120**	
MN rgp120/HIV-1 antigen	300 μg/ml
A244 rgp120/HIV-1 antigen	300 μg/ml
Aluminum hydroxide (alum) gel in sterile suspension	600 μg/ml
Total volume per vial	1.2 ml
Storage: Store refrigerated at 2ºC to 8ºC	
**AIDSVAX Placebo**	
Aluminum hydroxide (alum) gel in sterile suspension	600 μg/ml
Total volume per vial	1.2 ml
Storage: Store refrigerated at 2ºC to 8ºC	

6.4.2.2 Reconstitution of Vaccines/Placebos

ALVAC-HIV (vCP1521)

a) Add 1.0 mL 0.4% NaCl from a prefilled syringe
b) Roll mixture gently in vial
c) Withdraw 1.0 mL ALVAC-HIV (vCP1521), using syringe with preattached opaque membrane.

ALVAC Placebo: as per active vaccine

AIDSVAX® B/E

a) Roll mixture gently in vial; do not shake
b) Withdraw 1.0 mL (300 μg dose/antigen) AIDSVAX® B/E

AIDSVAX Placebo: as per active vaccine

6.4.2.3 Dosage Administration

Doses of ALVAC-HIV or ALVAC Placebo must be kept at 2-8ºC until preparation (DO NOT FREEZE). The vaccine should be given within 2 hours of reconstitution of vaccine. Administer contents intramuscularly into the left deltoid after preparation of the site with alcohol.

Doses of AIDSVAX® B/E and AIDSVAX Placebo must be kept at 2-8ºC until preparation (DO NOT FREEZE. DO NOT SHAKE). The vaccine should be given within 2 hour of being drawn into a syringe. AIDSVAX® B/E or AIDSVAX Placebo are administered intramuscularly into the right deltoid after preparation of the site with alcohol.

All vaccine injections will be given by members of the research team who have received specific training in detection and treatment of anaphylaxis. Each district clinical site where injections occur will be supplied with appropriate medications for emergency use if anaphylaxis occurs. The hospital will provide further backup to the clinical site for additional emergency treatment.

ALVAC-HIV will be administered at:
- Week 0
- Week 4 (window: weeks 3-6)
- Week 12 (window: weeks 10-14)
- Week 24 (window: weeks 21-27)

ALVAC Placebo will be administered at:
- Week 0
- Week 4 (window: weeks 3-6)
- Week 12 (window: weeks 10-14)
- Week 24 (window: weeks 21-27)

AIDSVAX® B/E will be administered at:
- Week 12 (window: weeks 10-14)
- Week 24 (window: weeks 21-27)

AIDSVAX Placebo will be administered at:
- Week 12 (window: weeks 10-14)
- Week 24 (window: weeks 21-27)

No modification of dosage for any of the vaccine products used will be allowed in this study.

6.4.2.4 Disposition

Investigators based at the district hospitals, or their designees are responsible for maintaining an accurate inventory and accountability record of vaccine supplied for this study. At the conclusion of vaccine administration, all vaccine supplies (including used, unused or partially used vials and unused or partially used syringes of ALVAC-HIV, ALVAC Placebo, ALVAC diluent, AIDSVAX® B/E, and AIDSVAX Placebo) must be documented, returned to the manufacturer (if specifically requested), or destroyed per sponsor SOP. Remaining materials may not be administered to other subjects or be used for any other experimental *in vitro* or animal model studies.

6.4.2.5 Precautions to be Observed in Administering Study Vaccine

As with any parenteral vaccine, epinephrine (a.k.a. adrenaline), antihistamines and corticosteroids must be available for immediate use should an immediate hypersensitivity reaction, such as anaphylaxis, occur. All study vaccines must be injected intramuscularly. As per SOP and training, the vaccine/placebo will not be injected intravenously.

Vaccinations will not be administered if volunteer has acute illness which would interfere with assessment of vaccine reaction (for example, manifested by fever defined as oral temperature of $\geq$ 38.2ºC). Also, vaccinations will not be given into an area of skin/muscle that is traumatized or has acute inflammation.

6.4.3 Method of Assigning Subjects to Study Groups

A specific randomization list will be generated for each district clinical site. Each of these sites will receive a different, site-specific randomization list. Only the independent statistician (and the data-base developer) will have a complete set of randomization lists. Vaccine and placebo will be equally (allocation ratio 1:1) distributed in randomized blocks. The vaccine and placebo will be assigned specific codes within the randomization list provided to each pharmacy nurse to further minimize the possibility of unintentional unblinding or bias:

1) All vaccine and placebo vials will be labeled with one of six potential letters provided by the independent statistician to the manufacturers. No one except the independent statistician, a limited number of identified individuals and manufacturers' labeling personnel will know which codes represent active agent or placebo. Manufacturers will not have access to any data linking a volunteer with the randomization code.
2) Pharmacy nurses will be trained in GCP and instructed not to discuss randomization lists, codes, or volunteer assignments with study personnel. These pharmacy nurses will need to sign a confidentiality agreement. They will be the only person(s) on site who will know the randomizatioin code.
3) The randomization code will not appear on any label or source document leaving the pharmacy.

6.4.4 Blinding Procedure

Volunteers and investigative site personnel will not know who is receiving vaccine or placebo. Since the ALVAC-HIV vaccine and ALVAC placebo products are not identical in appearance, to preserve blinding the syringes will be pre-coated with an opaque material which masks the difference. In addition, the person preparing vaccine syringes will not be involved in the clinical assessment of volunteers and will be instructed to not comment on the appearance of experimental agent to clinic staff. For all volunteers, the volume of injection will be consistent.

The investigative site personnel, as well as all personnel involved in the data management, monitoring or conduct of the trial, will be blinded to the study vaccine assignment. The pharmacy nurse will be the only person at the site who will have access to the randomization code of volunteers and possibly observe a visual difference in the preparation of vCP1521 and ALVAC Placebo. Pharmacy nurses will not be told which visual characteristic is associated with active or placebo agents – only that such exists and is normal. Moreover, the pharmacy nurse is not in the notification loop for unblinding and must sign a confidentiality agreement not to discuss randomization lists, codes or volunteer assignments. In case of vaccine-related death or life-threatening serious adverse events (SAEs), knowledge of whether a volunteer received vaccine or placebo can be critical for the

interpretation of the significance of clinical findings and thus impact decisions regarding continuation in/with the trial. In such cases, the assignment of a volunteer may be unblinded. A request for unblinding, with its rationale, should be forwarded through the Vaccine Trial Senior Investigator (VSI) to the Principal Investigator (PI). The PI will evaluate the request and will notify the Chairman of the Data and Safety Monitoring Board (DSMB) and the Study Medical Monitor (SMM). The Chairman of the DSMB and the SMM will evaluate the request and will advise the Sponsor regarding a course of action. The Sponsor will decide to unblind or not unblind. In the case of the former, the Sponsor will authorize the independent statistician to provide this information to the PI. It should be noted that there are very few circumstances in which unblinding will be essential to the medical management of a vaccine (or placebo) recipient. Episodes of unblinding, whether accidental or intentional, will be reported by the site investigator with an explanation to the sponsor representative at AFRIMS who will inform the IRBs, DSMB and manufacturers. Follow-up of such volunteers will continue through the duration of the trial.

Since HIV-1 ELISA and Western blot results (see Section 6.7.3) may reflect vaccine or placebo assignment, access to such data will be limited to the laboratory personnel who are performing the tests and managing the data, the independent statistician and the DSMB. If diagnostic testing of original and verification samples reveal true HIV-1 infection, the volunteer (via the clinic staff/physician), the PI, the Study Medical Monitor, and the Sponsor will be informed by the laboratory. HIV-infected subjects will be informed about their HIV-1 infection, but the subject, study site, investigators, Sponsor, Study Medical Monitor, and manufacturer staff will remain blinded as to their assignment group until the study is closed and the database is locked.

Some volunteers may be tempted to know their assignment to vaccine or placebo through voluntary HIV testing. Volunteers will be actively discouraged from having HIV testing outside of the trial protocol. If specific needs arise, the research team will provide HIV testing and assist volunteers who need such results. Further, volunteers will be counseled that if they may have had a high-risk exposure to HIV, they should have HIV testing done and that this should be done through the vaccine trial system.

6.4.5 Prior and Concomitant Medications and Vaccines

Information regarding concomitant medications used in association with an adverse event will be collected and recorded in source documents and on CRFs (see Section 6.7.2.2). Information pertaining to non-HIV vaccines, immunoglobulin preparations, immunosuppressive medication, and antiretroviral drugs will be elicited at study visits and recorded on source documents and CRFs (including any AEs which occur). Other vaccines to prevent HIV-1 infection and chronic use of immunosuppressives are prohibited. Live-attenuated vaccines should not be administered within 2 weeks of study vaccination. Medically indicated subunit or killed vaccines (e.g., hepatitis A, hepatitis B or rabies) or immunoglobulins should be given at least 2 weeks before, or 2 weeks after, HIV vaccinations to avoid potential biologic interaction and/or confusion of adverse reactions. However, if rabies, tetanus or other types of vaccine are indicated in the post-exposure setting, it will take priority over the study vaccine. If subjects require a brief course (< 10 days) of immunosuppressive drugs during the study, they may continue scheduled vaccinations provided they are not given within 7 days of stopping immunosuppressive therapy. Volunteers requiring longer courses of chronic immunosuppressive therapy will be discontinued from vaccinations. (Corticosteroids given in doses of < 20 mg prednisone equivalent, or duration ≤ 10 days, are not considered immunosuppressive.)

6.5 REFERRAL AND MANAGEMENT OF HIV-INFECTED VOLUNTEERS

6.5.1 Referral and Follow-up Procedures

a) If an HIV infection is suspected in a volunteer according to the diagnostic algorithm (Section 6.7.3), the laboratory will inform the site investigator. The volunteer will have an appointment 2-3 weeks after original blood draw for a Verification Visit (and a second one, if the first is not positive).

b) At the Verification Visit(s), the volunteer will be counseled based on the need for repeat testing to determine results. Blood will be collected for repeat HIV diagnostics, CD4 T cell count, viral load, virus isolation, genetic sequencing and drug resistance testing, and archiving of plasma and PBMCs.

c) The diagnostic results of the verification visit(s) will be provided to the volunteer at the visit scheduled 2-3 weeks later. If negative, the volunteer will be counseled accordingly and will be asked to submit a new blood sample for repeat verification testing. If the outcome of both verification tests are negative, the volunteer will return to the routine protocol visit schedule. If either verification test is positive, the diagnosis of HIV infection is established and the volunteer will be given post-test counseling for HIV positivity. Respecting confidentiality, the volunteer will be counseled to voluntarily inform his/her potentially exposed partner(s) of the HIV test result; the vaccine trial team will offer assistance in counseling and HIV testing for the partner(s). Risk behaviors will be assessed by questionnaire and focused counseling provided. A new blood draw will be performed for CD4 count, RNA viral load, and for plasma and PBMC archiving. The volunteer will be referred to an MOPH HIV treatment unit for medical evaluation and management. Laboratory results of HIV diagnosis, CD4 T-cell count and RNA viral load will be sent in a confidential manner to the site investigator who will provide the results to the volunteer's physician at the MOPH hospital.

d) Those volunteers who become HIV infected will continue to participate in the trial. Through the remainder of the trial after HIV infection is diagnosed, follow-up visits will be scheduled every 6 months (consistent with original study schedule) for counseling and follow-up safety assessments, CD4 count, viral load, virus isolation (if not previously successful), drug resistance testing (if on anti-retroviral drugs), other genetic characterization of infecting viruses (sieve analysis) and archives of plasma and PBMC (see Section 6.7.3). Concomitant medications used in association with an adverse event will be elicited and recorded on source documents and CRFs. Laboratory results of CD4 count and viral load/drug resistance testing will be provided confidentially to the treating physician. The timing of these follow-up visits is intended to help maintain confidentiality of the HIV status of the volunteer. Throughout the duration of the trial, blinding will be maintained for these infected volunteers, as for uninfected volunteers in the trial.

e) It is recognized that few clinical endpoints (AIDS-defining illnesses, CD4 count < 200, death) will occur among infected volunteers during the duration of this protocol (53). In order to gather this important, but supplementary, information, it is intended that a separate protocol will be developed and submitted through the full review and approval process.

f) As the RV152 protocol, which follows breakthrough infections in RV144, is allowed access to RV144 information in order to provide additional data for analysis, viral load and CD4 data collected in RV152 can be utilized in RV144. In addition, clinical samples will be shared between RV144 and RV152 to address study objectives in the two studies.

6.5.2 Treatment of Volunteers HIV Infected during Trial

The volunteers who become HIV infected (intercurrent infections) during the immunization phase of the trial (which may be detected via other health care interactions outside the trial's planned visits) will have no further vaccinations, and will be counseled by the research team and referred to receive medical services from the Ministry of Public Health (MOPH) hospitals in accordance with the most current national Guidelines for treatment. The MOPH will be responsible for medical services related to management of conditions caused by HIV infection. The medical services include clinical examination by a physician, necessary laboratory tests, and standard medications in accordance with the most current National Guidelines for Clinical Management of HIV/AIDS in Adults and Children issued by the MOPH. These volunteers will continue to be followed under this protocol and AFRIMS will provide CD4 counts and viral load results to the responsible clinician as described in 6.5.1 above. After the vaccine trial is completed, HIV-infected volunteers will continue for the rest of their lives to receive the medical services for HIV/AIDS from the MOPH.

Volunteers who acquire HIV during their participation in the efficacy trial will be provided care according to the most current Guidelines established by the Thailand Ministry of Public Health. The care will be provided by the MOPH. Additionally, the Sponsor has offered to provide the Royal Thai MOPH additional funding to offset the Government's procurement cost of anti-retroviral drugs. These funds will be available upon separate agreement between the Sponsor and the MOPH.

6.6 STUDY PROCEDURES AT EACH VISIT

Visit 1, day 0

- Sign protocol consent form
- Medical history and directed physical examination (study nurse will take medical history; if indicated, physician will perform physical examination)
- Elicitation/recording of concomitant medications
- Participation impact elicitation
- Risk behavior assessment (baseline)
- Risk behavior reduction education
- Blood sample for archiving of plasma and PBMCs (blood volume: 16 mL)
- Urine pregnancy test for women prior to the vaccination
- Assessment of vital signs
- **First vaccination**: inject ALVAC-HIV or ALVAC Placebo into the left deltoid muscle within 2 hours after reconstitution.
- Observe volunteer for systemic or local reaction for 30 minutes post-vaccination; enter findings on appropriate CRF.
- Instruct subjects on how to complete 3-day reactogenicity diary card and to contact the research team at the hospital if any significant adverse event occurs (as per SOP).

Visit 2, day 7 (window: -4 thru +7 days)

- Clinical assessment (Adverse Event elicitation)
- Participation impact elicitation
- Review of diary card with volunteer; enter findings on appropriate CRF
- Elicitation/recording of concomitant medications

Visit 3, week 4 (window: -1 thru +2 weeks)

- Clinical assessment (Adverse Event elicitation)

- Elicitation/recording of concomitant medications
- Participation impact elicitation
- Risk behavior reduction education
- Urine pregnancy test for women prior to the vaccination
- Assessment of vital signs
- **Second vaccination**: inject ALVAC-HIV or ALVAC Placebo into the left deltoid muscle within 2 hours after reconstitution.
- Observe volunteer for systemic or local reaction for 30 minutes post-vaccination; enter findings on appropriate CRF.
- Instruct subjects on how to complete 3-day reactogenicity diary card and to contact the research team at the hospital if any significant adverse event occurs.

Visit 4, week 5 (window: -4 thru +7 days)
- Clinical assessment (Adverse Event elicitation)
- Participation impact elicitation
- Review of diary card with volunteer; enter findings on appropriate CRF
- Elicitation/recording of concomitant medications

Visit 5, week 12 ± 2 weeks
- Clinical assessment (Adverse Event elicitation)
- Elicitation/recording of concomitant medications
- Participation impact elicitation
- Risk behavior reduction education
- Urine pregnancy test for women prior to the vaccination
- Assessment of vital signs
- **Third vaccination**: inject ALVAC-HIV or ALVAC Placebo into the left deltoid muscle. Then inject AIDSVAX® B/E or AIDSVAX Placebo into the right deltoid muscle within a few minutes of the first injection. All injections must be within 2 hours of reconstitution.
- Observe volunteer for systemic or local reaction for 30 minutes post-vaccination; enter findings on appropriate CRF.
- Instruct subjects on how to complete 3-day reactogenicity diary card and to contact the research team at the hospital if any significant adverse event occurs.

Visit 6, week 13 (window: -4 thru +7 days)
- Clinical assessment (Adverse Event elicitation)
- Participation impact elicitation
- Review of diary card with volunteer; enter findings on appropriate CRF
- Elicitation/recording of concomitant medications

Visit 7, week 24 ± 3 weeks
- Clinical assessment (Adverse Event elicitation)
- Elicitation/recording of concomitant medications
- Participation impact elicitation
- Risk behavior reduction education
- Pre-test counseling

- Blood sample for HIV diagnosis and archiving of plasma (blood volume: 6 mL)
- Urine pregnancy test for women prior to the vaccination
- Assessment of vital signs
- **Fourth vaccination**: inject ALVAC-HIV or ALVAC Placebo into the left deltoid muscle. Then inject AIDSVAX® B/E or AIDSVAX Placebo in the right deltoid muscle within a few minutes of the first injection. All injections must be within 2 hours of reconstitution.
- Observe volunteer for systemic or local reaction for 30 minutes post-vaccination; enter findings on appropriate CRF.
- Instruct subjects on how to complete 3-day reactogenicity diary card and to contact the research team at the hospital if any significant adverse event occurs.

Visit 8, week 26 (2 weeks post-4th vaccination; Window: -2 days to +14 days)
- Clinical assessment (Adverse Event elicitation)
- Elicitation/recording of concomitant medications
- Risk behavior assessment
- Risk behavior reduction education
- Participation impact elicitation
- Post-test counseling
- Blood sample for archiving of plasma and possibly PBMCs (blood volume: 8 mL)
- Review of diary card with volunteer; enter findings on appropriate CRF

Visit 9, week 52 ± 4 weeks
- Clinical assessment (Adverse Event elicitation)
- Elicitation/recording of concomitant medications
- Risk behavior assessment
- Participation impact elicitation
- Pre-test counseling
- Blood sample for HIV diagnosis and archiving of plasma and PBMCs (blood vol: 16 mL)

Visits 11, 13, 15, 17, 19 corresponding to weeks 78, 104, 130, 156, 182, respectively (± 4 wk)

- Clinical assessment (Adverse Event elicitation)
- Elicitation/recording of concomitant medications
- Risk behavior assessment
- Participant impact elicitation
- Pre-test counseling
- Blood sample for HIV diagnosis and archiving of plasma (blood volume for each visit: 6 mL)
- For visit 19, PBMCs will also be separated and archived from this blood collection (vol: 8 mL).

Visits 10, 12, 14, 16, 18, 20 corresponding to approximately 2-3 weeks after the 6-monthly visits

Volunteers will go to the project clinical site approximately 2-3 weeks after Visits 9, 11, 13, 15, 17, 19 for post-test counseling, risk behavior reduction education, adverse event elicitation (including concomitant medications) and participation impact elicitation. Visit 20 will also include completion of participant impact elicitation and Termination CRF. As mentioned in section 6.3.3 the volunteer may be seen at a mutually agreed upon location for non-vaccine visits.

6.7 STUDY VARIABLES AND THEIR MEASUREMENT

6.7.1 Study Primary Endpoints

Prevention of HIV Infection

The acquisition of HIV infection. Detection of HIV-1 infection will be defined according to the HIV diagnostic algorithm (Section 6.7.3) utilizing serologic and nucleic acid technologies. Incidence of HIV infection will be compared in the vaccine and placebo-recipient groups.

And

Changes in HIV-1 Viral Load[1]

Plasma viral load post-seroconversion is an important predictor of clinical progression of HIV infection and of transmissibility (54-56). The trial will quantitate HIV plasma viral load at the time of diagnosis and through the remainder of the follow-up period. Results will be compared in vaccine and placebo recipients who become HIV-infected during the trial. The trial is powered to detect a 0.39 log10 difference in viral load between vaccine and placebo recipients (see Section 6.9.3.2).

Secondary Endpoints

Changes in CD4 Cell Count

CD4 counts add prognostic value to viral load in terms of rate of disease progression (57). Two CD4 cell counts will be obtained (at the verification blood draw and the notification blood draw) and through the remainder of the follow-up period. Results will be compared in vaccine and placebo recipients who become HIV-infected during the trial. The trial is powered to detect a 26% difference in CD4 count between vaccine and placebo groups (see Section 6.9.42).

Safety

Reactogenicity, the frequency of local and systemic reactions, and both AEs and SAEs will be compared between vaccine and placebo groups (see Section 6.9.4.1).

Risk Behavior and Social Impacts

Volunteers may believe that the vaccine is protective against HIV infection and therefore modify their behavior in such a way that they increase their risk of exposure to HIV. There are safeguards against such increases in HIV risk behavior. The first is the repeated counseling in regard to the vaccines' unproven efficacy, the use of placebos, behavioral risk reduction, along with HIV pre- and post-test counseling. Any risk-taking behavior reported during counseling will be noted and addressed, for example nonuse of condoms or use of IV drugs. The second is the use of baseline and interval behavioral risk assessments (which will be recorded on CRFs and entered into the database). Using these data, significant increases from baseline in risk-taking behavior noted among volunteers will be reported and interventions specifically designed to decrease this behavior will be implemented (see Section 6.9.4.1). In addition to being asked whether their risk behavior has changed over time, participants will also be asked whether they have experienced any favorable or unfavorable events associated with their participation in the vaccine trial. These participation impact events (PIEs) include employment, school or personal relationship problems or benefits, discrimination, and positive

---

[1] Please see Analytic Addendum, **Section 18**, for primary analyses.

or negative health care experiences. If a participant does experience a PIE, its date and resolution status will also be recorded on the PIE CRF and entered into the database. Periodic assessment and review of both risk behavior change and PIEs will be carried out by the DSMB.

6.7.2 Safety Assessments

6.7.2.1 Post-vaccination Events Monitored as Possible Reactions to Vaccine

Selected local and systemic adverse events are routinely monitored in vaccine clinical trials as indicators of vaccine reactogenicity. These include (at the injection site) erythema, induration, pain/tenderness, swelling and limitation of arm movement, and (systemically) temperature, tiredness, myalgia, arthralgia, headache and rash and are herein termed "*post vaccination reaction*". It is recognized that each of these events, and particularly those of a systemic nature, may under some circumstances, in any individual subject, have another clearly defined diagnosis as a cause for these symptoms (for example, dengue fever), Those that have a clearly recognized cause not-related to the vaccination will not be reported as "*post-vaccination reactions*".

Instructions to Subjects Regarding Unusual or Severe Signs or Symptoms

Subjects will be given diary cards on which they are to answer questions about reactions that occur in the 3-day period after a vaccination. These will be returned to the district clinical site where they were vaccinated. All subjects will be instructed to seek medical attention by study personnel within the district where enrolled, if possible, or at another MOPH facility, if unusual or severe signs or symptoms occur after vaccination. Staff at health centers will refer volunteers to the district hospital for further evaluation and treatment as appropriate. These
subjects, if possible, will be followed up clinically until resolution of symptoms.

6.7.2.2 Other Adverse Events

An adverse event (AE) is any undesired, noxious or pathological change in a patient or subject as indicated by physical signs, symptoms, and/or laboratory changes that occurs following administration of one of the vaccines, whether or not considered vaccine related. This definition includes intercurrent illnesses or injuries, and unexpected exacerbations of pre-existing conditions. Anticipated day-to-day fluctuations of pre-existing conditions that do not represent a clinically significant exacerbation need not be considered adverse events. Discrete episodes of chronic conditions occurring during a study period should be reported as adverse events in order to assess changes in frequency or severity.

History of all adverse events occurring up to visit 10 that have resulted in an encounter with a health care provider (physician, nurse, etc) will be elicited, recorded on source documents and transcribed onto CRFs. Adverse Events that are trauma-related can include less information, including less medication information, than do medical conditions. After visit 10, only AE's that are "medically significant" events, defined as requiring multiple visits (two or more) to a physician for the same condition, or that result in hospitalization or an emergency room visit, will be captured on source documents/CRF's. Medications will continue to be reported in association with AEs. Data on serious adverse events occurring through the whole period of the study will be collected and recorded on CRFs, as well as reported separately, per SOP, on SAE report forms. A subject with an SAE will be followed carefully until the condition is resolved or stabilized and/or chronicity is established. Any medication or other therapeutic measure taken to relieve symptoms of the medical problem will be recorded on the CRF with the report of the outcome on the SAE forms.

The *intensity* reported on the adverse event form will be determined by the research staff using the following guidelines:

- Mild (Grade 1): Transient or mild discomfort; no limitation in normal daily activity.
- Moderate (Grade 2): Some limitation in normal daily activity.
- Severe (Grade 3): Unable to perform normal daily activity.
- Life threatening (Grade 4): Life threatening
- Death (Grade 5): Death

The *relationship of vaccination* to adverse event (AE) will be determined based on the following definitions:

Not Related:
Vaccination administration and AE occurrence not reasonably related in time; OR
AE obviously explained by another cause.

Unlikely Related:
Vaccine administration and AE occurrence remotely related in time; AND
AE more likely explained by other causes than by vaccination.

Possibly Related:
Vaccine administration and AE occurrence reasonably related in time; AND
AE explained equally well by causes other than vaccination.

Probably Related:
Vaccine administration and AE occurrence reasonably related in time; AND
AE more likely explained by vaccination than by other mechanisms.

Definitely Related:
Vaccine administration and AE occurrence reasonably related in time; AND
Vaccination most likely explains the AE; AND
AE is consistent with pattern of vaccine-related events.

(In analyses, vaccine-related AEs are defined as those AEs that are Possibly, Probably or Definitely Related to vaccination.)

A <u>serious adverse event</u> (SAE) is defined as any untoward medical occurrence that:

1. Results in death.
2. Is life threatening (i.e., the subject was, in the opinion of the investigator, at immediate risk of death from the event).
3. Requires or prolongs inpatient hospitalization. Hospitalization for either elective surgery related to a pre-existing condition that did not increase in severity or frequency following initiation of the study or for routine clinical procedures will not be considered an SAE.
4. Results in persistent or significant disability/incapacity (i.e., the event causes a substantial disruption of a person's ability to conduct normal life functions).
5. All pregnancies associated with congenital anomalies/birth defects.
6. Is an important and significant medical event that, based upon appropriate medical judgment, may endanger the subject and may require medical or surgical intervention to prevent one of the other outcomes defined above.

AE and SAE Management and Reporting Procedures

Adverse events (AEs) will be continuously ascertained and reported via CRF and DataFax to the clinical study database. All blinded reports of AEs within the clinical study database will be summarized by the US Military HIV Research Program (USMHRP) Data Coordinating and Analysis Center (DCAC) quarterly for the independent statistician (for the DSMB) and the Study Medical Monitor (SMM), and annually to meet regulatory reporting requirements of USAMMDA and RCQ.

Copies of these annual summary reports will be forwarded to AFRIMS for provision to the Study Principal Investigator who will forward to Thai regulatory authorities as required. By request from the DSMB, the Independent Statistician will prepare any unblinded safety reports.

SAEs are AEs and therefore ascertained and managed as above. In addition, SAEs require special reporting (via non-CRF SAE reporting forms), review and management. All SAEs will be submitted as a preliminary SAE report, and follow-up SAE reports will be provided as necessary until event resolution. All SAEs will be reported in a timely manner consistent with governing regulatory requirements. All SAEs identified at any clinical site are to be reported immediately to the Vaccine Trial Senior Investigator (VSI) who will forward to the study Principal Investigator.

PI: Dr. Supachai Rerks-Ngarm
Department of Disease Control
Ministry of Public Health
Nonthaburi, Thailand
Tel: 66 2 590 3006
Fax: 66 2 965 9569

VSI: Dr. Punnee Pitisuttithum
Faculty of Tropical Medicine
Mahidol University
Bangkok, Thailand
Tel: 66 2 354 9173
Fax: 66 2 354 9174

All SAE reports will be completed and signed by the Vaccine Trial Senior Investigator. The VSI must complete and transmit an initial SAE report within 24 hours of notification for all SAEs that are greater than grade 3 in intensity. In addition, all SAEs that are both related and unexpected, no matter what grade, must be reported within 24 hours. All other SAEs should be reported, by the VSI, within one week of their identification (routine SAE reports). The VSI will send the SAE report to the PI, the SMM, and the USMHRP Regulatory Operations Center (ROC).

The SMM will review, and officially establish, the vaccine relatedness and expectedness of all SAEs. For unexpected SAEs, the Local Medical Monitor (LMM) will be consulted (through the VSI), and his/her concurrence/nonconcurrence will be indicated on the SAE reports. The LMM will prepare an additional report in two cases: 1) unexpected SAEs that are vaccine-related; 2) unexpected SAEs where the LMM does not concur with the SAE report prepared by the VSI. The LMM sends this report (within 10 days) to the VSI, who forwards it through the ROC to RCQ.

The SMM forwards reviewed SAE reports to the USMHRP ROC which is the centralized consolidator, coordinator and forwarder of all RV144 SAE reports (both individual and periodic summaries). ROC is responsible for immediate SAE coordination with/between the SMM, Aventis Pasteur and VaxGen (required for all SAEs deemed to be vaccine-related) in order to develop the SAE trend component of the SAE report. ROC also provides (via input from DCAC) the study status component of the report. ROC is responsible for forwarding SMM- and ROC-vetted SAE reports to the DSMB, USAMMDA, RCQ and AFRIMS in a manner so as to allow MRMC to meet governing US and Thai regulatory timelines, and in compliance with RV144 SOPs. For expedited SAE reports, ROC will provide the DSMB, USAMMDA, RCQ and AFRIMS with completed reports within 48 hours of the Sponsor's knowledge of SAE occurrence. USAMMDA and RCQ are (together) responsible for further providing expedited reports required by the US FDA, and RCQ is responsible for further reporting to the HSRRB. For routine (non-expedited) SAEs, ROC will provide USAMMDA and RCQ with summary reports every 30 days, and will provide quarterly summaries to the DSMB and AFRIMS. AFRIMS will forward all ROC-provided reports to the study PI (who then has responsibility for meeting Thai institutional and regulatory reporting requirements). Copies of all SAE reports (individual and periodic summaries) forwarded by ROC to the DSMB, USAMMDA,

RCQ and AFRIMS (and thus to PI and Thai authorities) will also be provided to Aventis Pasteur and VaxGen

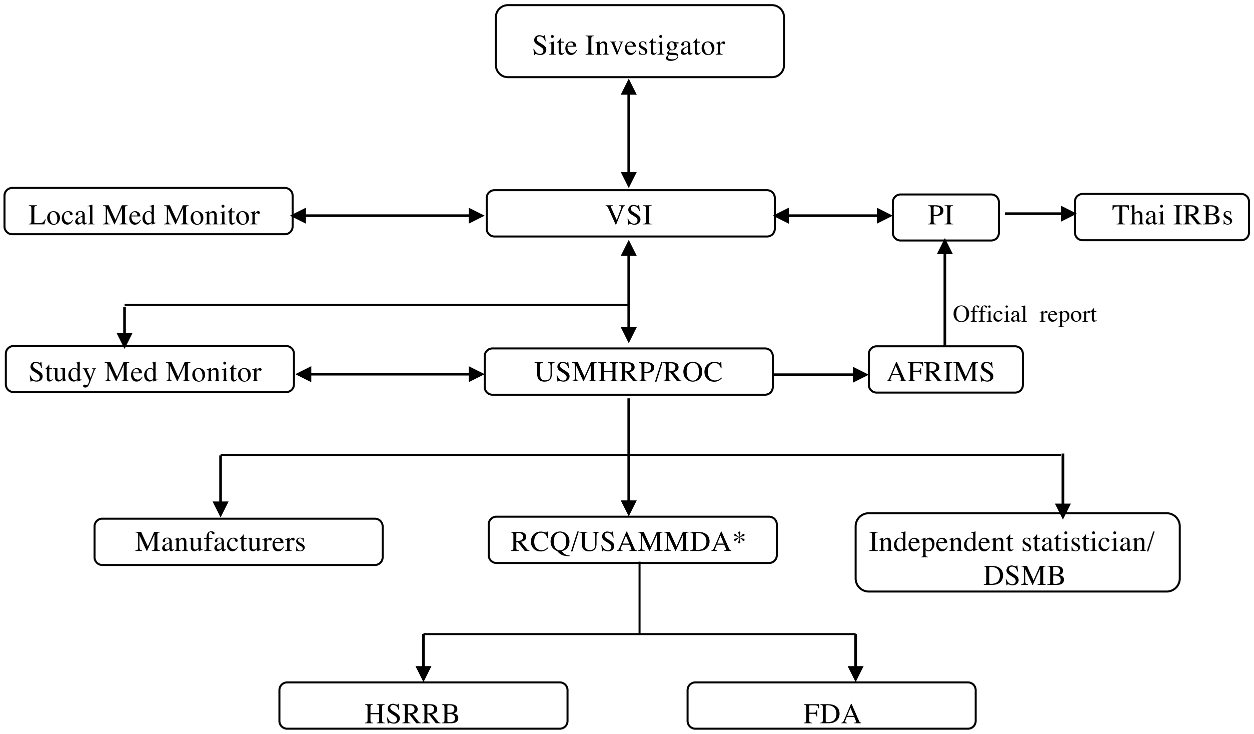

* Copies of FDA reports are also sent back to investigators and sites, as required.

ROC and DCAC will enter all SAEs into an administrative SAE database. An updated instance of the ROC/DCAC SAE database will be continuously available to USAMMDA and RCQ.

Hospitalization for normal, uncomplicated spontaneous vaginal delivery will not be considered an SAE. Pregnancy and pregnancy outcome are considered separate study events and are recorded on a pregnancy CRF. Adverse pregnancy outcomes will be recorded as SAEs.

Further vaccinations for any subject who has experienced a serious adverse event will be jointly decided upon by the study physician investigators and the Study Medical Monitor.

6.7.3 HIV Diagnostic Algorithm (see flow diagram below and Section 9.2)

Diagnostic HIV testing will utilize a sequence of validated tests that will differentiate between vaccine-induced seropositivity and true HIV infection. Information to the research staff of each vaccine trial site will not include the results of specific tests, but will state only HIV "infected" or "not infected", or that repeat testing is needed (as in the case of need for a verification specimen). Report of results will be delayed to at least ten days from blood collection so that the timing of HIV test reporting does not compromise the double-blind nature of the trial. (A result returned immediately after EIA testing could signal the clinical team that this specimen did not require the Western blot testing which would be needed if vaccine had induced an antibody response.)

An HIV-1 EIA assay will be utilized throughout the course of the protocol. If the EIA is reactive, the test will be repeated in duplicate. If repeatedly reactive, an HIV Western blot will be performed. If the Western blot is positive, HIV nucleic acid testing (Roche Amplicor or another clade-independent HIV RNA assay) will be performed on the plasma specimen stored at –70ºC. If the HIV RNA test is positive, a diagnosis of HIV infection is suspected, the research site is informed and the volunteer will be called back for counseling and repeat blood draw. A second blood specimen (verification specimen) will be obtained for complete repeat HIV diagnostics. If the second plasma specimen is positive both serologically and by nucleic acid testing, a diagnosis of HIV infection is considered established. If the verification specimen is not positive (negative or Western blot indeterminate), the volunteer will be counseled that one additional blood collection for retesting will be necessary. If results of this repeat verification specimen are positive, infection is established; if not positive, the volunteer will be informed that he/she is not HIV infected and will return to the protocol's regular visit schedule.

HIV Testing Algorithm

Diagnostic specimen (plasma)

↓
EIA → non-reactive → "Uninfected"
↓
Reactive
↓
EIA x 2 → both non-reactive → "Uninfected"
↓
Either reactive
↓
Western blot → not positive → "Uninfected"
↓
Positive[a,b]
↓
Nucleic Acid Test → negative → "Uninfected"
↓
Positive → perform quantitative RNA assay
↓
"Suspicious of HIV infection"
↓↓
↓↓

New volunteer appointment(s) for verification specimen(s)

→ A. Repeat testing algorithm, as above.

→ B. Blood also collected for CD4 count, HIV isolation, HIV RNA quantitation, genetic sequencing, drug resistance testing and archiving of plasma and PBMCs. (20 ml: 1 EDTA tube, 2 CPT tubes)

Verification specimen: **Neg (x 2)**[c] → "HIV uninfected"; return to routine F/U

Verification specimen: **Pos (x 1)** → "HIV infected"; inform site physician

→ 1) appt. for counseling and blood collection for CD4 and viral load and archiving of plasma and PBMCs (20 ml: 1 EDTA tube; 2 CPT tubes)

→ 2) refer to MOPH HIV Rx unit

→ 3) follow-up q 6 mo (from the first visit during which HIV infection was suspected) with counseling and blood draw for CD4, viral load, drug resistance testing (if on anti-retroviral drugs) and archiving of plasma and PBMCs. (20 ml: 1 EDTA tube; 2 CPT tubes)

[a] All Western blot-positive specimens will be tested in Thailand with one nucleic acid test (NAT) and in the United States with a second NAT. This redundancy will allow for further confirmation of study endpoints. If a discrepancy is found between the two NATs, the information will allow investigators to reassess a volunteer who may be infected but not yet diagnosed.

[b] At Visits 7 and 19, specimens with indeterminate patterns of reactivity on Western blot will undergo further analysis with NAT. If positive, these subjects will be requested to have a new blood collection for verification testing. The reason for this testing is to detect infected volunteers who have not developed diagnostic antibody responses (are in the "window period"). At Visit 7, this will provide maximal data for defining which subjects are uninfected at the completion of the immunization phase; at Visit 19, this will allow detection of these infections as part of the last follow-up visit.

[c] If the first verification specimen is found to be not HIV positive (negative or indeterminate), a second verification specimen will be collected and fully tested. If this repeat verification specimen is also negative, the volunteer is diagnosed as HIV uninfected; if positive, the diagnosis of HIV infection is considered established.

6.8 PROCEDURAL FLOW CHART

**Study Visit**	**1**	**2**	**3**	**4**	**5**	**6**	**7**	**8**	**9**	**10**	**11**	**12**	**13**	**14**	**15**	**16**	**17**	**18**	**19**	**20**
Week	0	1	4	5	12	13	24	26	52	54	78	80	104	106	130	132	156	158	182	184
Month	**0**								**12**		**18**		**24**		**30**		**36**		**42**	
**Administrative Requirements:**																				
Signed Consent Form	X																			
**Vaccinations:**	**X**		**X**		**X**		**X**													
Med. Hx ± Directed PE	X																			
Clinical Assessment (AE elicitation)	X	X	X	X	X	X	X	X	X	X	X	X	X	X	X	X	X	X	X	X
Diary Card Review		X		X		X		X												
Risk Assessment Questionnaire	X							X	X		X		X		X		X		X	
Participant Impact Elicitation	X	X	X	X	X	X	X	X	X	X	X	X	X	X	X	X	X	X	X	X
Risk Reduction Education	X		X		X		X	X		X		X		X		X		X		X
Pre-test Counseling							X		X		X		X		X		X		X	
Post-test Counseling								X		X		X		X		X		X		X
**Laboratory**																				
HIV Diagnostic Testing[1]							X		X		X		X		X		X		X	
Viral Load Test[2]							[X]		[X]		[X]		[X]		[X]		[X]		[X]	
CD4 Count[2]							[X]		[X]		[X]		[X]		[X]		[X]		[X]	
Urine Pregnancy Test	X		X		X		X													
**Archival specimens**																				
Plasma for HIV RNA							X		X		X		X		X		X		X	
Plasma for Thai National Repository[3]	X						X	X	X		X		X		X		X		X	
Plasma for Ab studies	X							X	X		X		X		X		X		X	
PBMCs	X							X	X										X	
**TOTAL BLOOD VOLUME PER VISIT (mL)**	16						6	8	16		6		6		6		6		8	

Total blood volume: 78 mL

[1] Will include standard EIA and WB; also nucleic acid tests if serology suggestive of infection.
[2] Will be performed in HIV-infected volunteers as part of verification blood draw, at time of giving diagnosis of HIV infection and then every six months until the completion of the trial.
[3] One specimen per volunteer at any time point.

6.9 ANALYTIC PLAN

6.9.1 Intent-to-Treat vs. Per-Protocol Analyses

Two types of efficacy analyses will be conducted: an intent-to-treat (ITT) analysis and a per-protocol analysis (PPA). The population for the per-protocol analysis is defined as individuals who meet all inclusion and exclusion criteria, complete the vaccination series and are HIV infection-free at the beginning of the 3-year follow-up period. Follow-up begins after the 24-week immunization period, following the last vaccination. The per-protocol analysis is restricted to volunteers who complied with the protocol by receiving all vaccinations within protocol-defined windows for vaccination.

The population for the intent-to-treat analysis is defined as all individuals who are randomized to either study arm during the accrual period. Unlike the per-protocol analysis, the intent-to-treat analysis will include individuals who have been randomized and become infected prior to the beginning of follow-up, i.e. during the immunization period. The intent-to-treat analysis will include individuals who did not comply with the protocol-defined vaccination schedule. Unlike the per-protocol analysis, the intent-to-treat analysis will include individuals who were not fully vaccinated.

Power and sample size calculations for the intent-to-treat analysis required making assumptions about vaccine efficacy during the vaccination phase, as well as during follow-up. In the following, it is assumed that vaccine efficacy during the vaccination phase is half of what is expected during follow-up.

6.9.2 Sample Size, Accrual and Follow-up

The target for recruitment is a total of 16,000 subjects. Individuals will be enrolled and randomly assigned to two study arms over approximately 2 years. The allocation ratio for the two study arms will be 1:1.

All participants will be followed for three years, after a 24-week immunization period, with assessment of HIV-1 infection occurring at semi-annual clinic visits. Power calculations are presented for a variety of assumptions. Sample size was determined using the conservative assumption that the annual HIV-1 seroincidence rate within the placebo arm will be approximately equal to the lower bound of the 95% confidence interval on incidence rates observed in studies within similar populations. A recent seroincidence cohort study of 20-40 year olds in Chon Buri Province found an incidence of 0.51/100 person-years, with a 95% confidence interval of 0.26 - 0.76/100 person-years. Among 20-30 year olds, the incidence was 0.68/100, with a CI lower bound of 0.34/100 P-Ys. Sample size calculations for this protocol assume an annual seroincidence of 0.34%, and that the rate of loss to follow-up will be 5% every 6 months including the vaccination phase. If the actual seroincidence rate is higher than the conservative estimate of 0.34%, then power will be higher. An exponential model is assumed for the time-to-infection data. In the study vaccine arm a piecewise relative hazard function was assumed in which vaccine efficacy was 25% during the 24-week immunization period and increased to 50% thereafter. Under these assumptions and with testing for an interim analysis and a final analysis as described in Section 6.9.3, a sample size of 16,000 is sufficient to allow power of 90.2% in an intent-to-treat analysis and 91.0% in a per-protocol analysis. A detailed explanation of sample size calculations can be found in Appendix 17.6.

The estimated dropout rate of 5% per 6 months is based on a cohort study carried out in Chon Buri Province during 1999-2001 (Benenson, et al., unpublished data). Assuming this loss to follow-up, we expect to accrue approximately 19,126 person-years of follow-up in each arm (vaccine and placebo) of the study, after the 24-week immunization period. As noted in Section 6.1, if the dropout rate during the vaccination phase exceeds 5%, additional subjects will be recruited and enrolled to

achieve 15,200 volunteers completing the 24-week vaccination phase, unless the length of time necessary to achieve 15,200 volunteers is unacceptable. Extending the enrollment period will have the effect of increasing the average time of entry into the study. The length of follow-up (3 years) will not change. If we assume that individuals enter the study uniformly throughout the enrollment period, extending the enrollment period by a specified amount should move forward the approximate time of the interim analysis by half of the number of months enrollment was extended. However, the exact timing of the interim analysis will depend to some extent on practical considerations, such as availability of the DSMB for review
of the interim analysis.

6.9.3 Analysis of Primary Endpoints

6.9.3.1 Primary Efficacy Analysis

The primary efficacy analysis will investigate protective efficacy of the investigational vaccine. Vaccine efficacy (VE) is defined as the relative reduction in HIV-1 infection incidence attributable to the vaccine. HIV infection is defined in Section 6.7.3. For analysis, the date of infection is defined as the midpoint between the draw dates of the last HIV-negative and first HIV-positive specimens. The draw date of the last negative specimen will be established by retrieving the last classified negative specimen and subjecting it to HIV nucleic acid testing (NAT). If the specimen is positive by NAT, previous specimens, beginning with the specimen from the draw immediately prior to this NAT-positive specimen, will be tested in reverse sequence until a negative NAT result is found. The draw date of the first positive specimen refers to the first specimen positive by NAT after the NAT-negative specimen is defined. In most cases this NAT-positive result will be from the same specimen that was determined to be diagnostically positive with the protocol's serologic/NAT testing algorithm. If a volunteer has an "HIV-positive specimen" and subsequently withdraws from the trial or is lost to follow-up such that no verification specimen is available for testing, this volunteer will be considered HIV infected for the purposes of the ITT and PPA analyses. Vaccine efficacy will be estimated using a continuous-time proportional hazards model using the exact procedure for handling ties. The models will be estimated using the SAS 'Phreg' procedure. Vaccine efficacy will be estimated using the estimated relative risk from the proportional hazards model [VE = 100 x (1-RR)]. Confidence intervals for vaccine efficacy will be obtained and will be based on transformations of confidence interval bounds for the relative risk parameter.

One interim efficacy analysis is planned. The purpose of the interim analysis is to monitor the trial for early, compelling evidence of definitive efficacy. The null hypothesis for the interim analysis is therefore specified as vaccine efficacy of 30%. The lower limit of the two-sided 95% confidence interval for vaccine efficacy will be determined using an O'Brien-Fleming error spending function boundary value (see Section 6.9.4). The interim analysis is scheduled at 2/3 information time. If the lower limit of the confidence interval exceeds the specified value of 30%, then the null hypothesis is rejected and the DSMB will consider a recommendation to stop the trial. Otherwise, the trial will continue to its planned conclusion and a final analysis will be conducted to evaluate the efficacy of the vaccine regimen.

The null hypothesis for the final analysis will be that true vaccine efficacy is equal to 0%. The statistical test at the planned conclusion of the trial will be a 2-sided 5% level test of the hypothesis that the risk of infection is the same at any given time in the two treatment groups. This test is equivalent to a 2-sided test of the null hypothesis that the hazard ratio is equal to one, or that the log hazard ratio is equal to zero. The test statistic will be compared to the O'Brien-Fleming error spending function boundary values determined for the end of the study period (see Section 6.9.4).

Two-sided 95% confidence intervals will be presented at the interim and final analyses to provide information about the precision of the estimates of vaccine efficacy.

Investigations of sample size and power were first conducted assuming no interim analysis. The null hypothesis used in the these power calculations matches the hypothesis in the final analysis of the actual planned design, that is, that true vaccine efficacy would be equal to 0%. Once the sample size for the trial was established, subsequent power calculations were conducted that took into account the interim analysis that was built into the design. In all cases, vaccine efficacy during immunization is assumed to be half the vaccine efficacy during follow-up. Probabilities were estimated through computer simulation of 10,000 data sets that were generated according to a proportional hazards model with 5% loss to follow-up occurring uniformly over each 6-month interval. Follow-up was assumed to occur over 3 years for each individual after a 24-week immunization period. An exponential model was assumed for the time-to-infection data with hazard rates determined by the assumed sero-incidence rates. The data were generated according to the model and then discretized to reflect the fact that determination of infection status will occur at 6-month intervals. Results were determined for both intent-to-treat and per-protocol analyses.

Table 3 summarizes estimates of the probability of rejecting the null hypothesis of 0% vaccine efficacy for a range of hypothesized true values of vaccine efficacy (assuming no interim analysis). In each of the 10,000 data sets, each with an assumed sample size of 8000 in each treatment group, a hypothesis test was conducted. The estimates in the second column of the table show how often in the 10,000 simulations the null hypothesis that vaccine efficacy is 0 was rejected for each assumed value of true vaccine efficacy. Under the null hypothesis that true vaccine efficacy is 0%, the probability of rejecting the null hypothesis is equivalent to the Type 1 error rate. Under the alternative hypothesis that true vaccine efficacy equals some non-zero value, the probability of rejecting the null hypothesis is equivalent to the power of the test. The statistical test upon which the calculations are based is a 2-sided 5% level test of the null
hypothesis that the risk of infection is the same at any given time in both groups.

Table 3. Power calculations/estimated probability of rejecting the null hypothesis H_o: VE = 0% for true VE ranging from 0 - 60% and median values for the lower limit (LL) of 2-sided 95% confidence intervals (CI) for VE when H_o was rejected (no interim analysis assumed)

**True VE (Phases: Vaccination/F-U)**	**Probability of rejecting H_o**		**Median LL of 2-sided 95% CI For VE when H_o rejected**	
	**Intent-to-Treat**	**Per-Protocol**	**Intent-to-Treat**	**Per-Protocol**
0% / 0%	4.9%	4.8%	4.6%	5.0%
15% / 30%	45.1%	45.1%	10.5%	11.4%
25% / 50%	90.8%	91.5%	23.0%	25.5%
30% / 60%	98.6%	98.6%	33.5%	37.2%

Note: annual seroincidence rate of 0.34% assumed.

Therefore, given the assumptions and considering an intent-to-treat analysis in the power calculations, the proposed trial size of 16,000 would have 90.8% power to detect a difference between vaccine and placebo at a 2-sided significance level of 0.05, if the true vaccine efficacy is 50% after full

immunization and 25% during immunization. Power will increase if true vaccine efficacy exceeds 50%, if the annual seroincidence rate exceeds 0.34%, or if loss to follow-up is less frequent.

Table 4 summarizes the final estimates of overall statistical power for varying hypothesized levels of true vaccine efficacy that are of practical interest. The final power estimation assumes that one interim analysis will be conducted at 2/3 information time. The null hypotheses for the interim and final analyses are as described above. Other provisions for the setup of the simulation exercise are the same as for the initial power estimation. By design, overall power is similar between the initial and final estimations for varying alternatives. When true vaccine efficacy is assumed to be 50% (with 25% in effect during the vaccination period), overall power is 90.2% and 91.0% for the intent-to-treat and per-protocol analyses, respectively. Given the strict testing criterion that is specified for the interim analysis, the probability is only 5.4% for detecting a statistically significance difference between the treatment groups in the early analysis (intent-to-treat) under this alternative. If the specification of the true vaccine efficacy is increased to 75%, (and 37.5% for the vaccination period), statistical power for the interim analysis is estimated to be 57.6% (intent-to-treat). Power is obviously negligible for interim testing (<1%) when true vaccine efficacy of 30% is considered, but the probability of accepting the null hypothesis in the interim analysis and subsequently rejecting the null hypothesis of 0% efficacy in the final analysis is 43.4% (intent-to-treat analysis) under this alternative.

Table 4. Final power calculations/Estimated overall power and estimated probability of rejecting the null hypothesis in the interim and final analyses with associated median estimates of vaccine efficacy (VE) and lower limit of the 2-sided 95% confidence interval (CI) for vaccine efficacy (VE) for varying levels of true vaccine efficacy (intent-to-treat ITT and per-protocol PP)

	**Interim Analysis**			**Final Analysis [1]**			
**Analysis Pop-ulation**	**Probability of rejecting H_0: VE=30%**	**LL of 95% CI (median*)**	**VE Estimate (median*)**	**Probability of rejecting H_0: VE=0%**	**LL of 95% CI (median*)**	**VE Estimate (median*)**	**Overall Statistical Power [2]**
True VE (Phases: Vaccination/F-U) = 15%/30%							
ITT	0.2%	32.9%	62.1%	43.4%	10.2%	36.9%	43.6%
PP	0.4%	33.6%	67.4%	43.5%	11.1%	39.9%	43.9%
True VE (Phases: Vaccination/F-U) = 25%/50%							
ITT	5.4%	34.5%	65.0%	84.8%	21.7%	46.4%	90.2%
PP	8.2%	36.2%	70.1%	82.8%	23.6%	50.2%	91.0%
True VE (Phases: Vaccination/F-U) = 37.5%/75%							
ITT	57.6%	41.0%	70.9%	42.4%	42.6%	63.2%	99.97%
PP	72.5%	45.5%	78.2%	27.5%	46.9%	68.5%	>99.99%

* Median value of the estimate when the null hypothesis is rejected.

[1] The presented probability of rejecting the H_0 in the final analysis is the probability of accepting the null hypothesis in the interim analysis AND rejecting the null hypothesis in the final analysis under the specified alternative.

[2] Overall statistical power refers to the probability of rejecting the null hypothesis under the specified alternative, at either the interim analysis (with H_0: VE=30%) or the final analysis (with H_0: VE=0%).

Note: annual sero-incidence rate of 0.34% assumed.

Confidence intervals were also calculated along with vaccine efficacy to show precision of the estimated vaccine efficacy in the estimations of statistical power. To get an idea of expected precision of estimated vaccine efficacy, median values for the lower limit of the 2-sided 95% confidence interval were obtained from the simulations under various assumed values of vaccine efficacy. When true vaccine efficacy was 50%, the median lower bound was equal to 23% among all simulations in which the null hypothesis was rejected using an intent-to-treat analysis with no interim testing (see Table 1). The median lower bound was 34.5% when the null hypothesis of 30% vaccine efficacy was rejected in the interim analysis (5.4% of the time) and the median estimate of vaccine efficacy was 65.0% (intent-to-treat), as shown in Table 2. Under this alternative, if the interim analysis null hypothesis was accepted and the null hypothesis of 0% vaccine efficacy was rejected in the final analysis, then the median lower bound estimate was 21.7% and the median vaccine efficacy estimate was 46.4%.

6.9.3.2 Viral load

There has been much discussion in the larger scientific community that suggests that viremic control may be a more realistic goal. It is well known, for example, that many established vaccines directly prevent morbidity and the consequences of the targeted disease without preventing infection (62).

After vaccination with various combinations of HIV vaccines, monkey challenge studies with chimeric SIV-HIV (SHIV) constructs have shown: 1) mitigation of disease rather than sterilizing immunity (reviewed in 63, 64-68); and 2) control of viremia rather than virus eradication (67,68). In humans infected with HIV there are substantial data that suggest that the level of HIV viral load shortly after resolution of acute infection is inversely correlated with disease progression (55, 56, 69-71). While it is unknown whether this would be similarly true for vaccinated individuals, animal studies using CD4 T cell loss or death as endpoints, provide further support for this hypothesis. Additionally, infectivity appears to be related to viral load, so that additional population benefits might accrue if viremia were controlled in vaccinated individuals (54,72,73).

Because of the potential advantages of a vaccine that successfully contributes to the control of viral replication, we propose to further highlight the importance of this endpoint by including viral load data in the primary analysis. This will maintain the commitment, as proposed in the study design, to ascertaining whether protection against infection occurs. Protection against infection remains a primary endpoint. Since the study size, monitoring plan, participant sampling etc. remain unchanged, this addendum only revises the way that already collected, supplementary data on viral load will be analyzed and interpreted.

To incorporate the viral load data into the primary analyses while maintaining the prior design structure, we will expand the experiment-wise error rate and include a test of viremia control. We will take advantage of the low variability, relative to the size of potentially important differences in viral load and add to the primary analysis a test for treatment differences in viral load with a two-tailed 1% Type 1 error rate. The study is adequately powered, in the absence of selection biases, to detect half-log differences in set-point viremia. Note that the randomization principle cannot be used to validate the significance levels of comparisons between selected subgroups, that is, there may be a selection bias. For example, if the vaccine protects only those individuals who are more likely to be protected from HIV-infection or disease progression regardless of vaccination, viral loads between HIV-infected vaccine and placebo recipients may spuriously appear to be lower in the placebo group. Thus we will

evaluate the vaccine causal effects, and viral load levels differences will be compared, protecting against maximal plausible selection bias (74,75).
The impact of this change will be to expand the experiment-wise Type I error rate, and it will be bounded by 6%. Thus 6% of the time, in the absence of a treatment effect, we will wrongly conclude one exists. The power of the revised final analyses is at least as great as that for the analysis used in the study design. Thus the benefits from the increased primary analysis Type I error are additional chances of claiming differences between vaccine and placebo when viral load is controlled, but infection rates are similar. A difference will be claimed when either infection-control or viremia-control occurs.

It is important to point out that results from the analysis of viral load data should be interpreted with caution. The analysis of this endpoint is a conditional analysis, since it is restricted to HIV-infected individuals, and these individuals are not a random sample from the HIV-infected population. There is a potential for selection bias since these individuals become infected post-randomization throughout the course of the study, and they are selected for the analysis because of their HIV infection status. In addition, due to the protocol-specified length of follow-up, the viral load endpoint is measured relatively early in the course of infection and limited inferences can be made about effects of vaccination on disease progression under this protocol.

Viral load measurements will be compared between HIV-infected vaccine and placebo recipients. Statistical tests will be carried out comparing the $\log_{10}$ viral load measurements obtained after the initial diagnosis of HIV infection at the set point (Section 6.7.1). A non-parametric Mann-Whitney test will be used for the comparison. The statistical power of a t-test for differences in the $\log_{10}$ viral load measurements between HIV-infected vaccine and placebo recipients was calculated. The calculations assume that vaccine efficacy is 25% during the immunization period and 50% after complete immunization, and that three viral load measurements are obtained on every participant who becomes infected during the trial. Detectable differences are calculated for 2-sided non-parametric Mann-Whitney tests at a significance level of 0.05. Under the anticipated study design there are expected to be approximately 43 infected vaccinees and 78 infected placebo recipients, including 10 vaccinees and 13 placebo recipients who become infected before complete immunization. Table 5 shows minimum detectable differences in $\log_{10}$ viral load for power of 80% and 90% for varying vaccine efficacy. Detectable differences were calculated using PASS 2000 with nonparametric adjustment to a t-test. Minimum detectable differences are presented for both an intent-to-treat analysis which includes those who become infected before full immunization, and also for a per-protocol analysis which excludes those who become infected during the immunization period. If vaccine efficacy is 50% and the viral loads in the two groups differ by 0.39 $\log_{10}$ or more, then this difference should be detectable with the current study design with at least 80% power.

Table 5. Detectable differences in HIV-1 viral load for 80% and 90% power – intent-to-treat and per-protocol analyses.

Type of Analysis	VE	Infected Vaccine Recipients (#)	Infected Placebo Recipients (#)	Detectable Difference	
				80% Power	90% Power
ITT	0.3	57	78	0.36	0.41
ITT	0.5	43	78	0.39	0.45
ITT	0.6	35	78	0.42	0.48
Per-Protocol	0.3	46	65	0.40	0.46
Per-Protocol	0.5	33	65	0.44	0.51
Per-Protocol	0.6	26	65	0.48	0.56

6.9.4 Analysis of Secondary Endpoints

6.9.4.1 Safety and Risk Behavior Data

Reactogenicity, adverse events and serious adverse events will be tabulated both overall and by study arm. Frequencies of specific safety events will be compared across study arms using a chi-square test to evaluate the null hypothesis that safety event rates are the same in both study arms.

Risk assessment data from the 'Baseline' and 'Interval' Risk Assessment CRFs will be tabulated both overall and by study arm. These questionnaires collect reported behavior data. The data will be analyzed for changes in behavior. Frequencies of specific risk behavior events at baseline and during follow-up will be compared across study arms using chi-square tests to evaluate the null hypothesis that the event rates remain the same. Specific risk behavior events will be compared (see Behavioral Risk Assessment). Repeated measures analyses of binary data will be performed to evaluate changes in the prevalence of these behaviors over time, overall and between the treatment groups. In addition, changes over time in proportions exhibiting specific behaviors will be assessed using McNemar's test. Participation impact events (from PIE CRFs) will also be tabulated overall and by study arm. Frequencies of events throughout the course of the study will be compared across study arms using chi-square tests to evaluate the null hypothesis that the event rates are similar in both study arms.

Previous studies using ALVAC-HIV (vCP1521) and AIDSVAX® B/E have shown that these vaccines alone and in combination (11,52) have good safety profiles. Previous and ongoing HIV vaccine studies conducted in Thailand have shown little evidence of increase in risk-taking behavior (27; TAVEG unpublished data). However, given the theoretical concern that antibodies generated by the prime-boost combination may prove to be enhancing, HIV infection rates in both groups will be monitored (as per previous analysis). In addition, the rate of disease progression as measured by viral load and CD4 count among infected vaccine recipients will be compared to the corresponding rates in placebo recipients.

The DCAC will provide data on safety and participation impact events quarterly to an independent statistician who is not a member of the DSMB. This statistician will have the ability to consult/confer with the DSMB if, in his/her opinion, there is significant asymmetry in the occurrence of HIV infection, AEs, SAEs, viral load and CD4 count at set point between the vaccine and placebo groups, or increased risk-taking behavior and adverse participation impact events. In the latter two cases, changes in counseling may need to be made to reduce risk-taking behavior or intervention from study staff may be needed to assist participants experiencing particularly unfavorable events due to their participation.

Interim safety monitoring will include looking for significantly higher rates of infection in the vaccine arm. Prior to the start of the trial, guidelines will be determined by the DSMB and the independent statistician that help to establish the need and frequency for DSMB safety reviews. The Z statistic for the hazard ratio at interim safety analyses will be compared to a continuous stopping boundary (58). A Type I error rate of 0.10 will be specified. Continuous stopping boundaries maintain Type I error and provide a flexible method for monitoring an ongoing trial by allowing for more frequent safety monitoring when the boundaries are almost crossed. If the boundary is crossed, a DSMB meeting will be convened and it is anticipated that the trial will be stopped. If the Z statistic approaches but does not exceed the continuous stopping boundary, this could raise concerns about the possibility of enhancement of the investigational vaccine, and the statistician will alert the DSMB chairperson. Such rules in addition to all relevant data will serve as guidelines for the DSMB in its decision-making
process.

6.9.4.2 CD4 T cell counts.

CD4+ T cell counts will be compared between infected vaccine recipients and infected placebo recipients. Statistical comparisons will be carried out using the nonparametric Mann-Whitney test. CD4+ T cell counts will be measured at the time of diagnosis of HIV infection and during subsequent follow-up. Statistical power of a test for differences in CD4+ T-cell counts between infected vaccine recipients and infected placebo recipients was calculated. The calculations assume that vaccine efficacy is 50% post-immunization (25% prior to full immunization) and that the comparison is made between CD4+ T cell counts measured 6 months post-diagnosis of HIV infection. The standard deviation was assumed to be approximately 235, and the mean among infected placebo recipients was assumed to be 505. The mean and standard deviation were obtained using the values reported for the mean and range of CD4+ T cell counts in subtype E infections (59). For the calculations below, the standard deviation was estimated to be the reported range divided by 4. Detectable differences were calculated for a 2-sided non-parametric Mann-Whitney test at a significance level of 0.05 for power of 80% and 90%. Detectable differences were calculated using PASS 2000 with nonparametric adjustment to a t-test. Under the anticipated study design, there are expected to be approximately 43 infected vaccinees and 78 infected placebo recipients, including 10 vaccinees and 13 placebo recipients who become infected before complete immunization. As Table 6 shows, if the CD4+ T-cell counts in the two groups differ by 26% or more, then this difference should be detectable by an intent-to-treat analysis with the current study design with at least 80% power if the expected number of infections occur.

Table 6. Detectable differences in CD4+ T cell counts for 80% and 90% power – intent-to-treat and per-protocol analyses.

				Detectable Difference (% of Placebo Mean)	
Type of Analysis	VE	Infected Vaccine Recipients (#)	Infected Placebo Recipients (#)	80% Power	90% Power
ITT	.30	57	78	118.7 (24%)	137.4 (27%)
ITT	.50	43	78	129.3 (26%)	149.6 (30%)
ITT	.60	35	78	139.1 (28%)	160.9 (32%)
Per-Protocol	.30	46	65	131.9 (26%)	152.6 (30%)
Per-Protocol	.50	33	65	146.4 (29%)	169.4 (34%)
Per-Protocol	.60	26	65	160.1 (32%)	185.3 (37%)

6.9.4.3 Correlates of protection and supplemental analyses.

Cytotoxic T lymphocytes (CTLs). Meaningful differences in CTL between infected and uninfected vaccine recipients would be difficult to detect. Only very large differences between the two groups would be detectable. To see this, if 20% of uninfected vaccine recipients develop CTLs (at a single time point) and a subset of 1000 uninfected recipients were assayed for CTL, then a study with 43 infected vaccine recipients would have 80% power to detect a significant difference if CTL rates were just 4.0% or less among infected vaccine recipients, using a two-sided two-sample comparison of proportions at significance level of 0.05. (Even if 5000 uninfected vaccine recipients were assayed for CTL, the study would have 80% power to detect a difference if CTL rates were just 4.3% or less among infected vaccine recipients.) Because of CTL test operating characteristics (sensitivity/specificy), CTL rates of approximately 5% or more are anticipated in both vaccine groups. Because of this and the relatively small expected number of infections among vaccinees, detecting CTL as a correlate of protection is not practical within current study parameters.

Neutralizing antibody (Nab). It may be possible to detect meaningful differences in Nab to T cell line-adapted or primary isolates, between infected and uninfected vaccine recipients. Development of neutralizing antibody will be compared between infected and uninfected vaccine recipients using a two-sided two-sample comparison of proportions. Assume for practical and economic reasons that only 150 of the uninfected vaccine recipients were assayed for Nab. Also assume that there are 43 infected vaccine recipients, as expected in the intent-to-treat analysis. Then if 70% of uninfected vaccine recipients develop Nab at a single time point, the study would have at least 93% power to detect a difference if Nab rates were 40% or less among infected vaccine recipients. If there are 33 infected vaccine recipients as expected in the per-protocol analysis, power would be 87% to detect the same difference.

6.9.5 Interim Efficacy Analysis (for interim safety analyses, see Section 6.9.4.1)

Under the study design assumptions, all participants will be followed for 36 months after the immunization phase with assessment for HIV infection occurring at six-month intervals. One interim efficacy analysis will be scheduled approximately 36 months after the beginning of the estimated 2-year accrual period, i.e. 36 months after the first enrollee completes the first visit. This time is estimated as the time at which the expected total patient exposure time is approximately 2/3 of what it is expected to be at the end of the trial. Results from the interim analysis will be presented to the

DSMB. The purpose of the interim analysis is to monitor the trial for early evidence of definitive efficacy. The following section outlines stopping rules assuming one interim efficacy analysis.

Two-sided O'Brien-Fleming error spending function boundary values for one interim analysis at 2/3 information time and at the end of the study period were determined using a Lan-DeMets spending function approach. Overall significance level was assumed to be 0.05. The software 'ld98.exe' that was used to calculate the values is described in Reboussin et al (60) and was downloaded from the University of Wisconsin website (/www.medsch.wisc.edu/landemets/). The absolute value for the two-sided O'Brien-Fleming error spending function boundary values is 2.5086 at 2/3 information time and 1.9929 at the end of the study period. If the timing of the interim analysis is changed from 2/3 information time due to practical considerations, then the boundary values will be recalculated.

The lower limit of the two-sided 95% confidence interval is used in the interim analysis to test the null hypothesis that vaccine efficacy is 30%. The method for determining repeated confidence intervals (61) draws upon the correspondence between statistical tests and confidence intervals. The lower limit of the two-sided 95% confidence interval for vaccine efficacy will be calculated as one minus the upper limit on the 95% confidence interval for the hazard ratio at the interim analyses. The upper limit of the two-sided 95% confidence interval for the hazard ratio will be determined using the O'Brien-Fleming boundary value that was computed for the analysis (i.e., 2.5086). At the interim analysis:

- If LL of 2-sided 95% CI for VE >30%, stop and reject the null hypothesis.

The null hypothesis for the final analysis will be that true vaccine efficacy is equal to 0%. The statistical test at the planned conclusion of the trial will be a 2-sided 5% level test of the hypothesis that the risk of infection is the same at any given time in the two treatment groups. The test statistic will be compared to the O'Brien-Fleming error spending function boundary values determined for the end of the study period.

If the null hypothesis at the interim analysis is accepted and after the final analysis:

- If $|Z_{final}| >= 1.9929$, reject the null hypothesis.

6.9.6 Interim Futility Analysis

At each meeting of the DSMB a futility analysis will be considered by the DSMB based on operational issues. The parameters used to determine futility are as follows:

1. Too few subjects volunteer for the trial
2. Too few subjects complete vaccination
3. Too few subjects complete follow-up
4. Too few cases of infection occur in the placebo group to detect an effect, and
5. Operational issues that compromise trial execution

At each DSMB meeting the conditional power will be evaluated. If conditional power is less than 10% under both the protocol assumptions and for the current trend then study closure would be recommended. The recommendation would be tempered if a trend for viral load control was observed and the precision of the estimate is inadequate (99%CI width >1.2log).

6.9.7 Additional Laboratory Studies and Data Analysis

RV144 assessed volunteer risk behavior at baseline and follow-up using a self administered behavioral questionnaire. Based on this assessment, 89% of volunteers reported one or less sexual partners and less than 5% reported high risk behavior (96). Hepatitis B can be transmitted through percutaneous or sexual exposure. Hepatitis C transmission almost exclusively occurs after

percutaneous exposure. These two infections are common in MSM and intravenous drug users. In addition, hepatitis C in intravenous drug users is associated with HIV infection. In Thailand, men who become HIV infected have a significantly higher rate of HCV infection (97). Herpes Simplex Virus – 2 (HSV-2) is also transmitted sexually and its prevalence is associated with past sexual activity. In addition, HSV-2 infection is associated with a two to fourfold increase risk of HIV acquisition (98). To better characterize a volunteer's behavioral risk classification during the trial and its impact on HIV acquisition and vaccine efficacy, we plan to analyze biomarkers (serology to HBV, Hepatitis C, HSV-2, syphilis) associated with HIV acquisition and high risk behavior using stored volunteer samples unlinked to any personal identifiers.

To evaluate whether pre-existing immunity against vaccine vector impacts on vaccine immune responses, we are also proposing to conduct anti-ALVAC antibody assay using stored volunteer samples.

The STEP study which tested a replicating defective Ad5 vector expressing HIV-1 Gag, Pol, and Nef found volunteers who received the vaccine and had high levels of pre-existing Ad5 antibody were at greater risk for acquiring HIV as compared to volunteers who received placebo (99). One possible mechanism for this enhancement in HIV infection is the preferential expansion by Adenovirus of memory CD4 T cell with mucosal homing phenotype that increases susceptibility to HIV infection in individuals with prior Adenovirus immunity (100). It is unclear whether this mechanism is specific to Adenovirus vector only or may apply to other non-replicating viral vectors. Analysis of RV144 result specifically did not show enhancement in HIV infection. However to exclude the possibility of uneven distribution of this potential confounder, we will conduct Ad5 antibody assay using archived samples.

Both Ad5 and anti-ALVAC antibodies will be determined using well established luciferas-based virus neutralization assay (101,102). Each assay will require 200ul of plasma. To determine distribution of baseline pre-existing Ad5 and anti-ALVAC antibodies, 200 random samples (100 placebos and 100 vaccinees) will be tested at baseline and 4 weeks post final vaccination.

As part of the pre-specified primary immunogenicity analysis, we have already conducted post vaccination CD4+ HIV-Specific intracellular cytokine staining (ICS), gp120 and p24 binding antibody; and lymphoproliferative assays on approximately 150 vaccine and 50 placebo recipients (96). Anti-ALVAC antibody assays will be conducted on the 150 individuals who received vaccine. To assess the impact of pre-existing anti-vector antibody on immunologic response, we intend to compare the proportion with ICS response and geometric mean binding antibody by presence or absence of pre-existing anti-ALVAC antibody.

To assess the impact of pre-existing on HIV acquisition, we intend to conduct a case control study comparing prevalence of baseline Ad5 and anti-ALVAC antibodies between volunteers who acquired HIV infection (case) and volunteers who remained free from HIV infection (controls). Controls will be matched to cases by gender, age and risk behavior at a 2:1 ratio.

6.10 DATA MANAGEMENT PLAN

The Data Management Unit (DMU) located at the Faculty of Tropical Medicine, Mahidol University, Bangkok, will be responsible for data management and analytic support for the trial. The data for study volunteers will be recorded on Case Report Forms (CRFs). A data management plan developed by the DMU and approved by the Sponsor will be implemented to cover procedures from handling completed CRFs to preparing cleaned datasets. The final dataset developed at the DMU will be the primary database for the trial. Laboratory tests will be performed at the Royal Thai and U.S.

Army laboratories at AFRIMS. The laboratory database will be maintained at AFRIMS with a password-protected version sent to the DMU as a backup.

Data management will be performed using DataFax technology (DataFax Inc., Hamilton, Ontario, Canada). The CRFs will be completed at clinical sites in the field and faxed to DMU. The DMU will compile the data and generate external QA/QC reports to be sent back to the clinical sites. Problems, if any, will be resolved with the clinicians and/or other responsible persons at the study sites and corrected CRFs faxed back to the DMU. The DMU will generate study progress and monitoring reports for designated personnel at the sites, as well as for related co-investigators.

Standard Good Clinical Practices (GCP) will be used to insure the accuracy, consistency, and reliability of the data. The study will be monitored for compliance with FDA regulations and GCP guidelines by designated personnel. The quality of the data will be monitored by the DMU and the Data Coordinating and Analysis Center (DCAC) of the U.S. Military HIV Research Program. The images of the CRFs and the raw data files will be periodically transferred by means of a password protected compact disc to the DCAC for validation of data integrity and generation of periodic safety reports. Any unresolved problems detected by the DCAC will be posed to the DMU to be incorporated into the QA/QC reports.

Cleaned datasets will be prepared by the DMU according to a pre-determined schedule of Data and Safety Monitoring Board (DSMB) meetings, and interim and final analyses. These cleaned and locked datasets will be sent to both the DCAC for summary and back-up and the independent statistician at EMMES Corporation (Rockville, MD) for interim and final analyses based upon a pre-approved data analysis plan. The independent statistician will provide analytic summaries based on the data analysis plan to the DSMB for review and consideration.

7. BENEFITS, COMPENSATION, RESEARCH-RELATED ILLNESS/INJURY, CONFIDENTIALITY AND ADVOCACY

7.1 BENEFITS

Benefits to the Individual Volunteer:

The volunteer will receive education and counseling about HIV/AIDS and the behaviors which could put a person at risk for acquiring or spreading this infection.

The volunteer will receive periodic HIV testing and counseling.

If the vaccines tested in this trial prove to be efficacious, the volunteers who received the placebos during the trial will be offered vaccination following completion of the trial with the HIV vaccines tested in this trial. The protocol does not cover vaccination of placebo recipients, but it is expected that they will be offered vaccination under a separate protocol under agreements between the MOPH and manufacturers.

Volunteers who become HIV infected during the trial will receive counseling in a confidential manner, and evaluation and treatment for their infection according to the National Guidelines (53).

Benefits to the Public Health of the Community:

The capacity of health care workers in the participating communities to provide counseling regarding HIV testing, risk behaviors and modes of transmission will be increased through participation in this vaccine trial.

Community members who are silently HIV infected will be detected through screening for this trial, and receive counseling as to HIV transmission in a confidential manner. Detection and counseling may decrease transmission of HIV in these communities and thus prevent potential infections.

7.1.1 Benefits and Risks to Volunteers who Become Prisoners

Benefits: Volunteers who become prisoners (if they choose to remain in the trial) will continue to receive counseling, risk reduction education, and testing for HIV. Those volunteers who become HIV infected will be followed identically to those in RV-144 who are not incarcerated and will receive the same level of care. The volunteers will be compensated for their participation and the money will be maintained in the prisoners account similarly to other money the prisoner may earn.

Risks: The potential risks to volunteers who remain in the trial are the interactions with prison authorities and other inmates. It is possible that the volunteer who chooses to stay in the trial will be discriminated against and may even be physically abused. This has not been a problem in other studies, and the fact that the volunteer continues in the study will be kept to a limited number of prison personnel.

7.2 COMPENSATION

Volunteers will be compensated for time lost from work, travel and meal expenses related to each scheduled study visit. Unusual travel expenses may be reimbursed if a receipt is presented. The standard compensation will be 500 baht per study visit (for time lost from work, travel, and meals).

7.3 RESEARCH-RELATED ILLNESS/INJURY

As stated in the consent form, participants who experience vaccine-related illness or injury will receive all necessary medical care and treatment free of charge provided by the Ministry of Public Health. Other than medical care, and other payments as stated in the consent form, there is no other compensation available from this research study.

7.4 CONFIDENTIALITY

Subject medical information obtained by this study will be maintained in a confidential manner, and disclosure to third parties other than those noted below is prohibited. Identifying information will be kept in locked file cabinets and password protected data bases which will be accessable only to the limited number of people with a justified need to know. Upon the subject's permission, medical information may be given to his/her personal physician or other appropriate medical personnel responsible for his/her welfare. Data generated by this study must be available for inspection upon request by representatives of the U.S. FDA, Thai national and local health authorities, the USAMRMC, VaxGen, Aventis Pasteur, and the approving IRBs, if appropriate.

7.5 ADVOCACY

Counseling of volunteers will include education regarding the potential social harm of "false positive" HIV tests and volunteers will be discouraged from having outside testing. All volunteers will receive an individual identification card indicating their participation in the HIV-1 vaccine trial. Volunteers will be advised to show this card if HIV testing is required or in other situations where they prefer to show their participation in the trial. All volunteers will be counseled periodically regarding the potential for testing positive on routine screening tests for HIV-1 as a consequence of participation in this trial and receiving this vaccine product. All volunteers will be offered further confirmatory testing and certification as to the nature of their vaccine trial participation whenever needed to address complications arising at home, at work, or in the community from routine screening for HIV-1.

The impact of participation in the trial will be monitored and tabulated. The trial staff will assist volunteers with specific problems identified. If general areas of concern are identified, efforts will be made by the research team to resolve the situation within the community.

8. VACCINE ACCOUNTABILITY

The Sponsor will be responsible for maintenance of the central vaccine storage facility and accurate records of vaccine lots received, held and shipped to clinical sites. The Principal Investigator or his/her designee must maintain accurate records of dates and quantities, and lots of product(s) received at clinical sites, to whom dispensed (subject-by-subject accounting), and accounts of any product accidentally or deliberately destroyed.

At the conclusion of the vaccine administration phase of the trial, all vaccine supplies (including used and unused vials) will be disposed of according to the procedures agreed upon (see Section 6.4.2.4). An overall summary of all vaccine supplies received, used and returned must be prepared at the conclusion of the study.

9. LABORATORY REQUIREMENTS

9.1 SPECIMEN PROCESSING AND STORAGE

1. Venipuncture will be done using standard sterile technique.
2. Blood will be collected into anticoagulant-containing tubes according to SOP; substitution of tube types may be made as long as these are appropriate and do not interfere with the performance of the studies in question. Tubes will be mixed by inversion immediately after collection.
3. Plasma will be separated within 30 hours. Plasma from visits 1, 8 & 9 will be divided into 5 equal aliquots; from other visits, into 4 equal aliquots.
4. PBMCs (from Visits 1, 9 and 19) will be processed and cryopreserved (-135ºC or lower) within 30 hours. There will be three equal aliquots. Procedures will be according to SOP. Additionally, PBMCs from Visit 8 may be archived although there would be no increase in volume of collected blood.

9.2 Laboratory Assays

HIV EIA: Both Thai and U.S. FDA-approved kits will be used. Clinical diagnosis of HIV infection will be based on testing plasma using a Thai FDA-approved EIA kit. Infection as a study endpoint will be based on a U.S. FDA-approved kit.

HIV Western blot: Both Thai and U.S. FDA-approved kits will be used. Clinical diagnosis of HIV infection will be based on testing plasma using a kit approved by the Thai FDA, such as the Sanofi New LAV Blot 1 or the Genelabs HIV Blot 2.2. Infection as a study endpoint will be based on a U.S. FDA-approved kit. Assay interpretation will be according to the manufacturer's instructions.

HIV nucleic acid tests: plasma collected in ACD or other suitable anti-coagulant will be assayed for HIV RNA using two NAT platforms. The Amplicor (Roche) version 1.5 kit has a detection range of 400 to 750,000 RNA copies/mL using the standard extraction procedure. The Procleix (Gen-Probe) TMA HIV Discriminatory test has a detection threshold of 100 RNA copies/mL.

CD4+ T cell count: CD4+ T cell count will be determined using EDTA anti-coagulated blood and standard flow cytometry according to U.S. CDC Guidelines for CD4 enumeration (57).

Immunogenicity. As measures of vaccine immunogenicity (lot-to-lot variation), the following assays may be utilized: binding antibody to HIV Gag, Env and/or canarypox; ELISpot, CTL and/or other assays of cellular immune response to vaccine antigens.

Immunologic correlates of protection. If this trial shows efficacy, correlates of protection may be sought using stored plasma and/or cryopreserved PBMCs. Assays for cellular immune response are improving rapidly and the specific assays to be employed with the limited specimens stored during the trial will be determined based on the level of validated technology at the time. Assays of humoral immunity assessed for a correlate of protection will include neutralizing antibody to subtype E and/or B HIV-infected cells, both lab-adapted strains and primary isolates. As the study found modest efficacy in preventing HIV infection, determination of correlates of protection using the most up to date and validated immunologic assays is critical. Assays that cannot be performed by the Sponsor or MOPH-TAVEG collaborators are conducted under contract with MHRP for the MOPH-TAVEG collaboration by outside research institutes in accordance to the RV144 MHRP – Thai MOPH MTA. Blinded, coded samples without personal identifying information are provided to recipient institutions for analysis only after issuance of MTA, U.S. Army ethical review and receipt of IRB approval from the institute contracted to conduct the assay.

Establishment of serum repository. The establishment of a sample repository to house and distribute coded specimens collected under RV 144 and RV 152 for future studies related to the objectives the protocols will be provide a mechanism for streamlining these collaborative scientific efforts. According to the Materials Transfer Agreement of 30 March 2009, the samples are reposed by the MHRP under the joint trusteeship of the Ministry of Public Health and the U.S. Army. Access to coded samples will be through submission of a project proposal to a joint Research Review committee composed of RV144 investigators. The Research Review Committee will require that a representative of the trustees (MOPH and the U.S. Army) be present and approve all requests. The committee will review the proposed study for scientific validity, availability of samples, and relevance to study objectives of the original protocols.

The committee will keep minutes of all meetings held for review of project proposals, review MTAs and agreements for transfer of materials, and ensure investigators receiving samples from this repository have local IRB approvals for the research by their institutions. A summary of the studies, their results and all associated regulatory documents will be provided in the annual continuing review report for RV 144.

All samples sent to laboratory investigators will be coded and contain no personal identifiers. The link between samples and research data will be maintained solely by Emmes Corporation under the responsibility of the Study Statistician (Dr. Don Stablein or designee). The Study Statistician will sign the attached affirmation that the link between personal identifiers and study data will not be shared under the rules of the repository. Investigators may ask the Study Statistician for information, but it will only be provided in a way that does not violate the terms of the code. For this reason, laboratory work arising from this repository will meet regulatory criteria for non-human subjects research (NHSR). The ethical committees or IRBs approving this amendment agree that the studies approved by the Research Review Committee of the Repository will not have specific IRB approval. While there will not be an IRB of record for studies approved by the Research Review Committee, the repository will be managed in a manner consistent with DoD and other regulatory body policies, and the recipient institution's IRB will have review and approving authority over these NHSR studies. An annual report of activities will be provided to ethical committees and IRBs reviewing this protocol and additional information on specific studies may be requested at any time.

Virology. The strains of infecting HIV may be studied to compare genotypic, phenotypic, and serotypic characteristics between infected vaccinees and placebo recipients. Infecting viruses will be classified according to categorical and continuous metrics that measure similarity to the antigens delivered in the candidate vaccines.

Host genetics. In conjunction with the immunogenicity and viral sequence analyses, the evaluation of host factors that may influence susceptibility to, or the course HIV-1 infection, or may contribute to the strength and breadth of adaptive immune responses will also be analyzed. Currently, polymorphic human genes of interest include human leukocyte antigen (HLA) Class 1 and 2 alleles as well as Killer Immunoglobulin Receptor (KIR) genes and potentially others. HLA Class I typing of study participants provides an opportunity to evaluate whether any of the frequent alleles (i.e. those present at 5% or greater that could exert population-level effects) or common extended haplotypes, influence the course of HIV-1 infection. Studies of the natural history of HIV-infection have consistently demonstrated the positive effect of HLA-B*57 alleles (76-80) In addition, both Class I and Class II responses are associated with both cellular and humoral vaccine responses (79,81-85). The innate immune system operates in parallel with adaptive immunity, and at some levels innate and adaptive immune systems have a direct interaction. Innate immunity may be a powerful controller of very early damage from HIV infection, and the natural killer (NK)-cells that mediate this effect are controlled by a series of receptors on their surfaces, called Killer Immunoglobulin-like Receptors (KIR, see reviews by Parham (86,87)). KIR genes are highly polymorphic and have been associated with differential rates of HIV disease progression. Hence, it may be useful to explore KIR genetic diversity in this study (88-90). It is possible that KIR may work synergistically with a vaccine to limit early immune destruction and spare the adaptive immune responses generated by vaccination. Similarly, polymorphisms in Fc gamma receptor (FcγR) have now been associated with differential

susceptibility to HIV infection in infants, and in recipients of AIDSVAX (91,92). The host restriction genes TRIM5α and APOBEC provide initial resistance to viral infections in general, and polymorphisms in these genes have now been associated specifically with altered HIV disease progression (93-95).

In addition, recent work from Division of Retrovirology, WRAIR has found a significant relationship between FcγR, KIR, and TRIM5α and acquisition of infection in HIV negative cohorts in East Africa (Kijak et al, unpublished data). These findings underscore the importance of innate immune responses in the prevention of HIV infection and mandate analysis of the vaccine and placebo recipients to ensure equal distribution of these polymorphisms was accomplished by randomization.

Genetic polymorphisms associated with susceptibility to HIV infection or with HIV disease progression should be evaluated; to include typing for immunogenetic markers, polymorphisms associated with host cell restriction and other gene directed analyses, as well as genome wide association studies. HLA, KIR, and FcγR, TRIM5α, and APOBEC3G typing will be done on all volunteers who become HIV-infected as well as a sample of approximately 500 HIV-negative volunteers for comparison, stratified by vaccination status. Additional genetic studies will be addressed with future amendments or study-specific protocols.

10. CASE REPORT FORMS

Case report forms (CRF) will be provided for each subject. All entries and corrections on CRFs and source documents should be made in ink. Correction of data on CRFs and source documents may be made only by drawing a single line through the incorrect data and writing the correct values, allowing the original text to remain legible. Each correction must be initialed and dated by the person making the change. If corrections are made after review and signature by the investigator, he/she must be made aware of the changes and document this awareness. For those CRF changes that are not obvious, a brief reason for the changes will be added.

It is the policy of the Sponsor that the study data must be verifiable to the source data, which necessitates access to any original recordings, laboratory reports, and subjects' records. The investigator must therefore agree to allow access to subjects' records, and source data must be made available for all study data. Note that certain source data will be directly recorded onto DataFax-compatible CRFs (e.g., behavioral risk assessments), as specified in SOPs.

11. STUDY MONITORING AND COMPLIANCE

All aspects of the study will be carefully monitored by the sponsor or authorized representatives of the sponsor, with respect to current Good Clinical Practices (GCP) and Standard Operating Procedures (SOP) for compliance with applicable government regulations. These individuals will have access, both during the trial and after trial completion, to review and monitor all records necessary to ensure integrity of the data, and will periodically review progress of the study with the Principal Investigator or designated representative(s).

Monitoring will be performed by Sponsor representatives at two levels. Within Thailand, AFRIMS' staff (CRAs) will perform internal monitoring of source documents and CRFs. External monitoring will be carried out by Sponsor (USAMMDA) representatives and/or contracted clinical research organization. At regular intervals, a team from the Sponsor, in association with manufacturer representatives, will audit trial conduct, visit the study sites and audit a subset of documents according to SOP.

Every attempt must be made to follow the protocol, and to obtain and record all data requested for each subject at the specified times. However, reasons may warrant the failure to obtain and record certain data, or to record data at the times specified. If this becomes necessary, the reasons for such must be clearly documented on the source document.

12. RETENTION OF RECORDS

Because data from this clinical trial may be used to support regulatory filings in several countries throughout the world, the policy concerning record retention reflects the current ICH guidelines. To comply with these guidelines, it is requested that the investigator arrange for the retention of case report forms, source records, and other supporting documentation for a minimum of 2 years after the last approval of a marketing application in an ICH region or at least 2 years have elapsed since formal discontinuation of clinical development of the investigational product.

Volunteer identities will be entered into the U.S. Army's Medical Research and Materiel Command's Volunteer Registry Database utilizing demographic information collected at the beginning of a volunteer's participation in the trial and completed after close of study and unblinding. This confidential database is intended to ensure that research volunteers can be adequately warned of risks and provided new information about the vaccines as it becomes available.

13. USE OF INFORMATION AND PUBLICATION

It is understood by the investigators that the information generated in this study will be used by the sponsor in connection with the development of the product and therefore may be disclosed to government agencies in various countries. To allow for the use of information derived from the study, it is understood that the investigator is obliged to provide the sponsor with complete test results, all study data, and access to all study records.

The sponsor and manufacturers recognize the importance of communicating medical study data and therefore encourages their publication in reputable scientific journals and presentation at seminars or conferences, while protecting the integrity of the ongoing trial.

Any results pursuant to the conduct of this protocol and/or publication, lecture, manuscripts based thereon, shall be exchanged and discussed by the investigators, the manufacturers' representative(s) and the WRAIR/USAMRMC prior to submission for publication or presentation. Results from investigations shall not be made available to any third party by the investigating team outside the publication procedure as outlined above. Within any presentation or publication, confidentiality of individual subjects will be maintained, with identification by subject code number and initials, if applicable.

14. PROTOCOL AMENDMENTS

Amendments to the protocol will be made only after consultation and agreement between Sponsor and investigators. The only exception is where the investigators consider that a subject's safety is compromised if action is not immediate. In such circumstances the investigators must inform the Sponsor and all overseeing review boards within 5 working days after the emergency occurred. All amendments to the protocol and revisions of the informed consent document, must be reviewed and approved by the MOPH IRB and HSRRB prior to their implementation. These changes will also be submitted to other IRBs and regulatory agencies.

15. REFERENCES

1. UNAIDS. AIDS Epidemic Update: December 2001. UNAIDS Website [unaids.org].

2. Thailand's response to AIDS – "Building on success, confronting the future". World Bank Report, November 2000.

3. Quinn TC. Global burden of the HIV pandemic. Lancet 1996; 348:99-106.

4. Esparza J, Bhamarapravati N. Accelerating the development and future availability of HIV-1 vaccines: why, when, where, and how? Lancet 2000; 355: 2061-6.

5. Mason CJ, Kitiripornchai S, Markowitz LE, et al. National surveillance of HIV-1 prevalence and subtype in young Thai men. J AIDS 1998; 19:165-73.

6. Mascola JR, Louwagie J, McCutchan FE, et al. Two antigenically distinct subtypes of human immunodeficiency virus type 1: viral genotype predicts neutralization serotype. J Infect Dis 1994; 169:48-54.

7. VanCott TC, Bethke FR, Artenstein AW, et al. Serotyping international HIV-1 isolates by V3 peptides and whole gp160 proteins using BIAcore. Methods: A Companion to Methods Enzymol. 1994; 6:188-98.

8. McCutchan FE, Salminen MO, Carr JK, Burke DS. HIV-1 genetic diversity. AIDS 1996; 10 (suppl 3):S13-20.

9. Mascola JR, McNeil JG, Burke DS. AIDS vaccines: are we ready for human efficacy trials? JAMA 1994; 272:488-9.

10a. Klein M. AIDS and HIV vaccines. Vaccines 1999; 17(Suppl 2):S65-70.

10b. Klein M. The prime-boost concept applied to HIV preventive vaccines. AIDS 1997; 11 (Suppl A):S127-37.

11. Pitisuttithum P, Chernow M, Berman P, *et al*. Initial Report of a Phase I/II Trial of AIDSVAX® B/E rgp120 HIV Vaccine - Bangkok, Thailand. *XIII International AIDS Conference, Durban, South Africa, 9-14 July 2000* [Abstract WePpA1362].

12. National AIDS Committee. National Plan for Prevention and Alleviation of HIV/AIDS, 1997-2001. Bangkok, 1997 [ISBN 974-7896-44-3].

13. Esparza J. Development of WHO-sponsored sites for HIV vaccine evaluation. AIDS Res Hum Retrovir 1993; 9:S133-4.

14. Nitayaphan S, Brown AE. Preventive HIV vaccine development in Thailand. AIDS 1998; 12 (suppl B):S155-61.

14a. Thongcharoen P. HIV Vaccine Evaluation Centers in Thailand. Report on International Seminar on Nuclear War and Planetary Emergencies, 24th session. Erice, Italy, August 1999, pages 59-67.

15. Thongcharoen P, et al. Future planning for HIV vaccine evaluation of Thai AIDS Vaccine Evaluation Group (TAVEG) in Thailand. 12th World AIDS Conference, Geneva [abstract 33222].

16. Nitayaphan S, Khamboonruang C, Sirisophana N, et al. A phase I/II trial of HIV SF2 gp120/MF59 vaccine in seronegative Thais. Vaccine 2000; 18:1448-55.

17. Pitisuttithum P, Nitayaphan S, Tongcharoen P, et al. Safety and immunogenicity of combinations of recombinant subtype E and B HIV-1 envelope gp120 vaccines in healthy Thai adults. J Infect Dis 2003; 188:219-27.

18. Reference deleted with update of protocol.

19. Berman PW, Gregory TJ, Riddle L, et al. Protection of chimpanzees from infection by HIV-1 after vaccination with recombinant glycoprotein gp120 but not gp160. Nature 1990; 345:622-5.

20. Berman PW, Murthy KK, Wrin T, et al. Protection of MN-rgp120-immunized chimpanzees from heterologous infection with a primary isolate of human immunodeficiency virus type 1. J Infect Dis 1996; 173:52-9.

21. Belshe RB, Graham BS, Keefer MC, et al. Neutralizing antibodies to HIV-1 in seronegative volunteers immunized with recombinant gp120 from the MN strain of HIV-1. JAMA 1994 ; 272: 475-80.

22. Gorse GJ, Yang EY, Belshe RB, Berman PW. Salivary antibodies induced by human immunodeficiency virus type 1 recombinant gp120 vaccine. Clin Diag Lab Immunol 1996; 3: 769-73.

23. Eastman D, Sheppard HWC, Dondero D, et al. Evaluation of commercial HIV diagnostic assays with sera obtained from volunteers immunized with recombinant gp120 based subunit vaccines. *XIII International AIDS Conference, Durban, South Africa, 9-14 July 2000*. [Abstract MoPeA2135].

23a. Berman PW, Huang W, Riddle L, et al. Development of bivalent (B/E) vaccines able to neutralize CCR5-dependent viruses from the United States and Thailand. Virology 1999;265: 1-9.

24. Judson FN, Mayer K, Gorse GJ, et al. Safety and immunogenicity evaluation of HIV-1 vaccines AIDSVAX® B/B and AIDSVAX® B/E - preliminary results. *XIII International AIDS Conference, Durban, South Africa, 9-14 July 2000*. [Abstract WePpA1363].

25. Harro C, Judson F, Brown SJ, *et al*. Successful recruitment and conduct of the first HIV vaccine efficacy trial in North America and Europe. *XIII International AIDS Conference, Durban, South Africa, 9-14 July 2000*. [Abstract WeOrC556].

26. Choopanya K, Bangkok Vaccine Evaluation Group (BVEG). Initiation of a Phase III efficacy trial of bivalent B/E rgp120 HIV vaccine (AIDSVAX® B/E) in Bangkok, Thailand. *XIII International AIDS Conference*, *Durban, South Africa, 9-14 July 2000*. [Abstract WeOrC555].

27. Pitisuttithum P, Bangkok Vaccine Evaluation Group (BVEG). Social harm monitoring among injecting drug users (IDUs) in a phase III HIV vaccine trial in Bangkok, Thailand - preliminary results. *XIII International AIDS Conference*, *Durban, South Africa, 9-14 July 2000*. [Abstract TuOrD448].

28. Jermano J. Monitoring of social and behavioral harms in the Phase III Trial of AIDSVAX B/B. *XIII International AIDS Conference, Durban, South Africa*, *9-14 July 2000*. [Abstract ThOrD679].

29. Taylor J, Paoletti E. Fowlpox virus as vector in non-avian species. Vaccine 1988; 6: 466-8.

30. Baxby D, Paoletti E. Potential use of non-replicating vectors as recombinant vaccines. Vaccine 1992; 10: 8-9.

31. Plotkin SA, Cadoz M, Meignier B, et al. The safety and use of canarypox vectored vaccines. Dev Biol Stand 1995; 84:165-70.

32. Haffar O, Garrigues J, Travis B, Moran P, Zarling J, Hu S-L. Human immunodeficiency virus-like, nonreplicating, gag-env particles assemble in a recombinant vaccinia virus expression system. J Virol 1990; 64:2653-2659.

33. Fang Z-Y, Kuli-Zade I, Spearman P. Efficient human immunodeficiency virus (HIV)-1 Gag-Env pseudovirion formation elicited from mammalian cells by a canarypox HIV vaccine candidate. J Infect Dis 1999; 180:1122-32.

34. Ferrari G, Humphrey W, McElrath MJ, et al. Clade B-based HIV-1 vaccines elicit cross-clade cytotoxic T lymphocyte reactivities in uninfected volunteers. Proc Natl Acad Sci USA 1997; 94:1396-401.

35. Clements-Mann ML, Weinhold KL, Matthews TJ, et al. HIV-1 immune responses induced by canarypox (ALVAC)-gp160 MN, SF2 rgp120, or both vaccines in seronegative adults. J Infect Dis 1998; 177:1230-46.

36. Moss B. Vaccinia virus: A tool for research and vaccine development. Science 1991; 252:1662-7.

37. Egan MA, Pavlat WA, Tartaglia J, et al. Induction of human immunodeficiency virus type 1 (HIV-1)-specific cytolytic T lymphocyte responses in seronegative adults by a nonreplicating, host-range-restricted canarypox vector (ALVAC) carrying the HIV-1 MN *env* gene. J Infect Dis 1995; 171:1623-7.

38. Fleury B, Janvier G, Pialoux G, et al. Memory cytotoxic T lymphocyte responses in human immunodeficiency virus type I (HIV-1)-negative volunteers immunized with a recombinant canarypox expressing gp160 of HIV-1 and boosted with a recombinant gp160. J Infect Dis 1996; 174:734-8.

39. Excler JL, Plotkin S. The prime-boost concept applied to HIV preventive vaccines. AIDS 1997; 11(suppl A):S127-37.

40. Pialoux G, Excler JL, Riviere L, et al. A prime-boost approach to HIV preventive vaccine using a recombinant canarypox virus expressing glycoprotein 160 (MN) followed by a recombinant glycoprotein 160 (MN/LAI). AIDS Res Hum Retroviruses 1995; 11:373-81.

41. Zolla-Pazner S, Alving C, Belshe R, et al. Neutralization of a clade B primary isolate by sera from human immunodeficiency virus--uninfected recipients of candidate AIDS vaccines. J Infect Dis 1997; 175:764-74.

42. Salmon-Ceron D, Excler JL, Finkielsztejn L, et al. Safety and immunogenicity of a live recombinant canarypox virus expressing HIV-1 gp120 MN tm/gag/protease LAI (ALVAC-HIV, vCP205) followed by a p24E-V3 MN synthetic peptide (CLTB-36) administered in healthy volunteers at low risk for HIV infection. AIDS Res Hum Retroviruses 1999; 15:633-45.

43. Tartaglia J, Excler JL, El Habib R, Limbach K, Meingnier B, Plotkin S, Klein M. Canarypox virus-based vaccines: prime-boost strategies to induce cell-mediated and humoral immunity against HIV. AIDS Res Hum Retroviruses 1998; 14 (Supp.3): S291-8.

44. Zolla-Pazner S, Xu S, Burda S, Duliege AM, Excler JL, Clements-Mann ML. Neutralization of syncytium-inducing primary isolates by sera from human immunodeficiency virus (HIV)-uninfected recipients of candidate HIV vaccines. J Infect Dis 1998; 178: 1502-6.

45. Verrier F, Burda S, Belshe R, Duliege AM, Excler JL, Klein M, Zolla-Pazner S. A human immunodeficiency virus prime-boost immunization regimen in humans induces antibodies that show interclade cross-reactivity and neutralize several X4, R5 and dual-tropic clade B and C primary isolates. J Virol 2000; 74:10025-33.

46. The AIDS Vaccine Evaluation Group 022 Protocol Team. Cellular and humoral immune responses to a canarypox vaccine containing human immunodeficiency virus type 1 env, gag, and pro in combination with rgp120. J Infect Dis 2001; 183:563-70.

47. Belshe RB, Gorse GJ, Mulligan MJ, et al. Induction of immune responses to HIV-1 by canarypox virus (ALVAC) HIV-1 and gp120 SF2 recombinant vaccines in uninfected volunteers. AIDS 1998; 12:2407-15.

48. Belshe RB, Stevens C, Gorse GJ, et al. Safety and immunogenicity of a canarypox vectored HIV-1 vaccine with or without gp120: a phase 2 study in higher and lower risk volunteers. J Infect Dis 2001; 183:1343-52.

49. Kim J, Robb M, Cox J, et al. Humoral and cellular HIV-specific responses induced by the prime-boost combination of Aventis-Pasteur ALVAC-HIV (vCP205) and oligomeric HIV-1 gp160MN/LAI-2 in HIV-uninfected adults. Eighth Conference on Retroviruses and Opportunistic Infections, Chicago, IL, 5-8 February 2001. [Abstract 179].

50. Evans TG, Keefer MC, Weinhold KJ, et al. A canarypox vaccine expressing multiple HIV-1 genes given alone or with rgp120 elicits broad and durable CD8+ CTL responses in seronegative volunteers. J Infect Dis 1999; 180:290-8.

51. Suriyanon V, Gurunathan S, Thoongcharoen P, et al. Safety and tolerability of live recombinant ALVAC-HIV (vCP1521) priming with either gp160 or gp120 boosting in Thai seronegative adults. Sixth International Congress on AIDS in Asia and the Pacific, Melbourne, Australia, 5-10 Oct 01. [Abstract 1034].

52. Nitayaphan S, Pitisuttithum P, Karnasuta C, et al. Safety and immunogenicity of an HIV subtype B and E prime-boost vaccine combination in HIV-negative Thai adults. J Infect Dis 2004, in press.

53. Rangsin R, Chiu J, Khamboonruang C, et al. The natural history of HIV-1 infection in young Thai men after seroconversion. J Acquir Immunoe Defic Syndr 2004; 36:622-9.

54. Quinn, TC, Wawer MJ, Sewankambo N, et al. Viral load and heterosexual transmission of HIV-1. N Engl J Med 2000; 342:921-9.

55. Daar ES, Lynn H, Donfield S, et al. Effects of plasma HIV RNA, CD4+ T lymphocytes, and the chemokine receptors CCR5 and CCR2b on HIV disease progression in hemophiliacs. J AIDS 1999; 21:317-25.

56. Lyles RH, Munoz A, Yamashita TE, et al. Natural history of HIV-1 viremia after seroconversion and proximal to AIDS in a large cohort of homosexual men. J Infect Dis 2000; 181:872-80.

57. Centers for Disease Control and Prevention. Guidelines for performing single-platform absolute CD4+ T-cell determinations with CD45 gating for persons infected with HIV. MMWR 2003; 52(RR02); 1-13.

58. Betensky. Construction of a Continuous Stopping Boundary from an Alpha Spending Function. Biometrics 1998, 54: 1061-1071

59. Hu DJ, Vanichseni S, Mastro TD, et al. Viral load differences in early infection with two HIV-1 subtypes. AIDS 2001; 15:683-91.

60. Reboussin DM, DeMets DL, Kim K, Lan KK. Programs for computing group sequential boundaries using the Lan-Demets method: Version 2. Technical Report, Department of Biostatistics, University of Wisconsin.

61. Jennison C, Turnbull BW. Group sequential methods with applications to clinical trials. Boca Raton: CRC Press, 1999.

62. Clements-Mann ML. Lessons for AIDS vaccine development from non-AIDS vaccines. AIDS Res Hum Retroviruses 1998; 14(Suppl 3):S197-203.

63. Robinson HL. New hope for an AIDS vaccine. Nature Rev Immunol 2002; 2:239-250.

64. Barouch DH, et al. Control of viremia and prevention of clinical AIDS in rhesus monkeys by cytokine-augmented DNA vaccination. Science 2000; 290:486-492.

65. Amara RR, et al. Control of a mucosal challenge and prevention of AIDS by a multiprotein DNA/MVA vaccine. Science 2001; 292:69-74.

66. Shiver JW, et al. Replication-incompetent adenoviral vaccine vector elicitis effective anti-immunodeficiency virus immunity. Nature 2002; 415:331-335.

67. Barouch DH, et al. Reduction of simian-human immunodeficiency virus 89.6P viremia in rhesus monkeys by recombinant modified vaccinia virus Ankara vaccination. J Virol 2001; 75:5151-5158.

68. Robinson HL, et al. Neutralizing antibody-independent containment of immunodeficiency virus challenges by DNA priming and recombinant pox virus booster immunization. Nature Med 1999; 5:526-534.

69. Mellors JW, et al. Prognosis in HIV-1 infection predicted by quantity of virus in plasma. Science 1996; 272:1167-1170.

70. Mellors JW, et al. Plasma viral load and CD4+ lymphocytes as prognostic markers of HIV-1 infection. Ann Intern Med 1997; 126:946-54.

71. Gilbert PB, et al. What constitutes efficacy for a human immunodeficiency virus vaccine that ameliorates viremia: issues involving surrogate end points in Phase 3 trials. J Infect Dis 2003; 188:179-93.

72. Gray RH, et al. Probablility of HIV-1 transmission per coital act in monogamous, heterosexual HIV-1 discordant couples in Rakai, Uganda. Lancet 2001; 342:921-9.

73. Longini IM, et al. Measuring vaccine efficacy for both susceptibility to infection and reduction in infectiousness for prophylactic HIV-1 vaccines. J Acquir Immun Defic Syndr Human Retrovirol 1996; 13:440-7.

74. Hudgens M, Hoering A, Self S. On the analysis of viral load endpoints in HIV vaccine trials. Stat Med. 2003; 22:2281-98.

75. Gilbert P, Bosch R, Hudgens M. Sensitivity analysis for the assessment of causal vaccine effects on viral load in HIV vaccine trials. Biometrics. 2003; 59:531-41.

76. Bailey JR, Williams TM, Siliciano RF, Blankson JN. Maintenance of viral suppression in HIV-1-infected HLA-B*57+ elite suppressors despite CTL escape mutations. J Exp Med 2006 May 15;203(5):1357-69.

77. Flores-Villanueva PO, Yunis EJ, Delgado JC, Vittinghoff E, Buchbinder S, Leung JY et al. Control of HIV-1 viremia and protection from AIDS are associated with HLA-Bw4 homozygosity. Proc Natl Acad Sci U S A 2001 April 24;98(9):5140-5.

78. Hendel H, Caillat-Zucman S, Lebuanec H, Carrington M, O'Brien S, Andrieu JM et al. New class I and II HLA alleles strongly associated with opposite patterns of progression to AIDS. J Immunol 1999 June 1;162(11):6942-6.

79. Kaslow RA, Rivers C, Tang J, Bender TJ, Goepfert PA, El Habib R et al. Polymorphisms in HLA class I genes associated with both favorable prognosis of human immunodeficiency virus (HIV) type 1 infection and positive cytotoxic T-lymphocyte responses to ALVAC-HIV recombinant canarypox vaccines. J Virol 2001 September;75(18):8681-9.

80. Kiepiela P, Leslie AJ, Honeyborne I, Ramduth D, Thobakgale C, Chetty S et al. Dominant influence of HLA-B in mediating the potential co-evolution of HIV and HLA. Nature 2004 December 9;432(7018):769-75.

81. Craven DE, Awdeh ZL, Kunches LM, Yunis EJ, Dienstag JL, Werner BG et al. Nonresponsiveness to hepatitis B vaccine in health care workers. Results of revaccination and genetic typings. Ann Intern Med 1986 September;105(3):356-60.

82. Desombere I, Willems A, Leroux-Roels G. Response to hepatitis B vaccine: multiple HLA genes are involved. Tissue Antigens 1998 June;51(6):593-604.

83. Ferrari G, Neal W, Jones A, Olender N, Ottinger J, Ha R et al. CD8 CTL responses in vaccines: emerging patterns of HLA restriction and epitope recognition. Immunol Lett 2001 November 1;79(1-2):37-45.

84. Gelder CM, Lambkin R, Hart KW, Fleming D, Williams OM, Bunce M et al. Associations between human leukocyte antigens and nonresponsiveness to influenza vaccine. J Infect Dis 2002 January 1;185(1):114-7.

85. Paris R, Bejrachandra S, Karnasuta C, Chandanayingyong D, Kunachiwa W, Leetrakool N et al. HLA class I serotypes and cytotoxic T-lymphocyte responses among human immunodeficiency virus-1-uninfected Thai volunteers immunized with ALVAC-HIV in combination with monomeric gp120 or oligomeric gp160 protein boosting. Tissue Antigens 2004 September;64(3):251-6.

86. Parham P. Immunogenetics of killer-cell immunoglobulin-like receptors. Tissue Antigens 2003 September;62(3):194-200.

87. Parham P. MHC class I molecules and KIRs in human history, health and survival. Nat Rev Immunol 2005 March;5(3):201-14.

88. Alter G, Martin MP, Teigen N, Carr WH, Suscovich TJ, Schneidewind A et al. Differential natural killer cell-mediated inhibition of HIV-1 replication based on distinct KIR/HLA subtypes. J Exp Med 2007 November 26;204(12):3027-36.

89. Martin MP, Gao X, Lee JH, Nelson GW, Detels R, Goedert JJ et al. Epistatic interaction between KIR3DS1 and HLA-B delays the progression to AIDS. Nat Genet 2002 August;31(4):429-34.

90. Martin MP, Qi Y, Gao X, Yamada E, Martin JN, Pereyra F et al. Innate partnership of HLA-B and KIR3DL1 subtypes against HIV-1. Nat Genet 2007 June;39(6):733-40.

91. Brouwer KC, Lal RB, Mirel LB, et al. Polymorphism of the Fc receptor IIa for IgG in infants is associated with susceptibility to perinatal HIV-1 infection. AIDS 2004; 18:1187-1194.

92. Forthal DN, Gilbert PB, Landucci G, Phan T. Recombinant gp120 vaccine-induced antibodies inhibit clinical strains of HIV-1 in the presence of Fc receptor-bearing cells and correlate inversely with HIV infection rate. J Immunol 2007; 178:6596-6603.

93. Speelmon EC, Livingston-Rosanoff, Li SS, et al. Genetic association of the antiviral restriction factor TRIM5α with human immunodeficiency virus type 1 infection. J Virol 2006; 80:2463-2471.

94. Javanbakht H, An P, Gold B, et al. Effects of human TRIM5α polymorphisms on antiretroviral function and susceptibility to human immunodeficiency virus infection. Virology 2006; 354:15-27.

95. An P, Bleiber G, Duggal P, et al. APOBEC3G genetic variants and their influence on the progression to AIDS. J Virol 2004; 78:11070-11076.

96. Rerks-Ngarm S, Pitisuttithum P, Nitayaphan S, Kaewkungwal J, et al. Vaccination with ALVAC and AIDSVAX to prevent HIV-1 infection in Thailand. N Engl J Med. 2009 Dec 3;361(23):2209-20.

97. Paris R, Sirisopana N, Benenson M, et al. The association between hepatitis C virus and HIV-1 in preparatory cohorts for HIV vaccine trials in Thailand. AIDS. 2003;17(9):1363-7.

98. Corey L, Wald A, Celum CL, et al. The effects of Herpes Simplex virus-2 on HIV-1 acquisition and transmission: A review of rwo overlapping epidemics. J AIDS 2004; 35:434-445.

99. Buchbinder SP, Mehrotra DV, Duerr A, et al. Efficacy assessment of a cell-mediated immunity HIV-1 vaccine (the Step Study): a double-blind, randomised,placebo-controlled, test-of-concept trial. Lancet 2008; 372:1881-93.

100. Benlahrech A, Harris J, Meiser A, et al. Adenovirus vector vaccination induces expansion of memory CD4 T cell with a mucosal homing phenotype that are readily susceptible to HIV-1. PNAS 2009 Nov 24;106(47):19940-45.

101. Duke-Cohan JS, Wollenick K, Witten EA, *et al.* The heterogenicity of human antibody response to vaccinia virus revealed through use of focused protein arrays. Vaccine 2009; 27(8):1154-65.

102. Sprangers M, Lakhai W, Koudstaal W, *et al*. Quantifying adenovirus neutralizing antibodies by luciferase-transgene detection: addressing pre-existing immunity to vaccine and gene therapy vectors. J. Clin Microbiol; 41:5046-5052.

16. SIGNATURE PAGE

A Phase III Trial of Aventis Pasteur Live Recombinant ALVAC-HIV (vCP1521) Priming With VaxGen gp120 B/E (AIDSVAX® B/E) Boosting in Thai HIV-uninfected Adults

I have read the foregoing protocol and agree to conduct the study as outlined. In addition, I agree to conduct the study in compliance with all applicable regulations and guidelines as stated in the protocol and other information supplied to me.

[signature] 22 - 04 - 16

Signature of Principal Investigator **Date**

On behalf of the Ministry of Public Health of the Royal Thai Government, I authorize the Principal Investigator to carry out this protocol with full support of the appropriate Ministry staff and infrastructure.

Name, Title and Signature of Representative, MOPH **Date**

On behalf of U.S. Army Medical Research and Materiel Command, I confirm that the Sponsor will comply with all obligations as detailed in all applicable regulations and guidelines. In addition, I will ensure that the investigator is informed of all relevant information that becomes available during the conduct of this study.

Signature of Sponsor's Representative **Date**

As the Independent Statistician, and on behalf of The EMMES Corporation, I confirm that all statistical and analytic reports will be prepared consistent with the protocol's analytic plan and provided exclusively to the Data and Safety Monitoring Board in support of the execution and analysis of this study.

Name, Title and Signature of Independent Statistician **Date**

17. APPENDICES

17.1 STATEMENT OF OBLIGATIONS OF CLINICAL MONITORS, CLINICAL INVESTIGATORS AND MEDICAL MONITORS

The Sponsor or his/her designated representative, will:

1. Conduct a pre-investigation visit to:
   a. Establish the acceptability of the facilities and record this in a written report (memorandum or form).
   b. Discuss the proposed clinical trial with the investigator, supply the case report forms, the Investigator Brochure, and the draft protocol for review and approval.
   c. Discuss with the investigator U.S. FDA requirements with respect to informed consent, Institutional Review Board (IRB) approval of the trial, the protocol, including protocol amendments and informed consent changes.
2. Conduct periodic on-site visits to:
   a. Assure adherence to the protocol.
   b. Review case report forms and medical records for accuracy and completeness of information.
   c. Examine pharmacy records for documentation of: quantity and date of receipt of investigational vaccine, disposition and accountability data for vaccine administration to each subject, loss of materials, contamination, etc, and unused supplies.
   d. Record, report (summarize) observations on the progress of the trial and continued acceptability of the facilities; prepare an on-site visit report.
   e. Review investigator files for required documents, i.e., protocols, protocol amendments, IRB approvals (protocols, amendments, informed consent, etc), IRB charter and membership, communications to and from the IRB and the sponsor.

<u>Clinical Investigators (including PI and Senior Investigators: Vaccine Team Senior Investigator (VSI), Laboratory Senior Investigator and Data Management Senior Investigator)</u>

1. Institutional Review Boards (IRB)
   The investigator must assure the monitor that the IRB:
   a. Meet U.S. FDA regulations as defined in 21 CFR Part 56.
   b. Have authority delegated by the parent institution and found in IRB by-laws, operation guidelines or charter to approve, or disapprove, clinical trials and protocols including informed consent and other documents (protocol amendments, information to be supplied to subjects concerning informed consent, etc).
   c. Comply with proper personnel makeup of IRB.
   d. Convene meetings using acceptable rules of order for making decisions, recording such decisions and implementing them.
   e. Files contain (a) documentation of its decisions such as are found in IRB minutes and correspondence, (b) written guidelines or by-laws governing IRB functions, (c) protocols, (d) protocol information to be supplied to the subject, (f) correspondence between IRB and investigator (consent changes, protocol amendments, etc).
2. Informed Consent of Human Subjects.
   The investigators must assure monitor that the informed consent for a subject:
   a. Meets U.S. FDA regulations as defined in 21 CFR Part 50 Informed Consent.

b. Has been approved by the IRB, including, when required, information to be given to the subject regarding the trial he/she is enrolled in.

(1) Informed consent includes the basic elements and any additional elements necessary.

(2) The subject and a study site representative sign the form and the subject is given a copy.

3. Storage and Dispensing of Vaccine Supplies.

The investigators (or pharmacist) must assure (demonstrate to) the monitor that:

a. Adequate and accurate written records show receipt and disposition of all vaccine supplies, including dates, serial or lot number, quantities received, each quantity dispensed, administered or used with identification of each subject.

b. Purpose and reasons are given in written records for vaccine disposal, i.e., the amount contaminated, broken, or lost, etc, and quantity returned to the sponsor.

4. Case Report Forms.

The investigators must assure the monitor that:

a. Case report forms, when completed, accurately reflect the medical records on each subject or patient.

b. Case report forms and medical records will be accessible to the monitor or U.S. FDA inspectors' on-site visits.

5. Files and Records.

The investigators must assure the quality, integrity, and content of his/her files that will be inspected by the monitor and may be inspected by U.S. FDA inspectors. The files must contain, at a minimum:

a. Correspondence to and from IRBs, Sponsor and the monitor.

b. Documents that include:

(1) IRB-approved protocols.

(2) IRB-approved protocol amendments.

(3) IRB-approved informed consent and information supplied to the subject or subject.

(4) IRB charter, membership, and their qualifications.

c. Documents and records must be retained for the period of time defined in Section 12 above.

b. Clinical supplies:

(1) Record of receipt, date and quantity, batch or lot number.

(2) Disposition dates and quantity administered to each subject.

(3) Inventory records.

Study Medical Monitor

1. The Medical Monitor oversees the progress of the clinical trial and ensures that it is conducted, recorded, and reported in accordance with the protocol, standard operating procedures (SOPs), GCP, and the applicable regulatory requirements.

2. The Medical Monitor must be a physician qualified by the training and/or experience required to review care to research subjects for conditions that may arise during the conduct of the research, and who monitors human subjects during the conduct of research.

Local Medical Monitor

1. The Local Medical Monitor serves as an independent physician who can be approached for medical information by volunteers, act as their advocate and assess their medical care for events which occur during the course of the trial. He also cooperates with the Study Medical Monitor to oversee the progress of the clinical trial and ensure that it is conducted, recorded, and reported in accordance with the protocol, standard operating procedures (SOPs), GCP, and the applicable regulatory requirements.

2. The Local Medical Monitor must be a physician qualified by the training and/or experience required to provide care to research subjects for conditions that may arise during the conduct of the research, and who monitors human subjects during the conduct of research.

Regulatory Operations Center (ROC)

Centralized consolidator, coordinator and forwarder of study data, especially serious adverse event (SAE) information, to the DSMB, USAMMDA, RCQ, AFRIMS, Aventis Pasteur and VaxGen. ROC is an element of the USMHRP and located within the Division of Retrovirology, WRAIR.

17.2 VACCINE TRIAL STUDY SITES

Chon Buri Province

Si Racha District

Bang Lamung District

Phan Thong District

Sattahip District

Rayong Province

Muang District (including MOPH STD Clinic)

Ban Chang District

Klaeng District

Ban Khai District

17.3 INFORMED CONSENT PROCESS AND CONSENT FORM (ATTACHED)

Selected communities in Rayong and Chon Buri Provinces will be provided with information regarding the planned vaccine trial. This will include group meetings with video presentations and question/answer sessions. Interested individuals will be further informed and offered enrollment into a screening protocol carried out through their local MOPH health center or district hospital. Volunteers who pass the screening process, which includes a Test of Understanding, and continue to be interested in participation will be given additional specific information about the phase III trial, a copy of the consent form and given an appointment at the district clinical site for enrollment and Visit 1 of this trial. During the approximately two weeks prior to Visit 1, the volunteer will consider the study and consent form, and discuss with family or others as preferred. At Visit 1, the research team will address any additional concerns of the volunteer prior to his/her giving written consent to enroll in the vaccine trial.

17.4 TEST OF UNDERSTANDING (FROM SCREENING PROTOCOL RV148)

17.5 PREGNANCY REPORTING

Study volunteers who become pregnant during the course of this study will continue on the study with all scheduled visits, but no further immunizations will be given. For all subjects who become pregnant during the trial, a Pregnancy Report CRF should be completed as soon as possible. Sites should maintain contact with pregnant subjects to obtain pregnancy outcome information for the Pregnancy Follow-up CRF. If a volunteer is pregnant at the last study visit (V20), the pregnancy outcome will not be recorded on the CRF so the study record can be sealed. Additional pregnancy outcome data will be passively collected, recorded on source documents, and reported to the sponsor and regulatory authorities.

Specific information to be collected includes the following:

1. Pregnancy Report
   - Date of last menstrual period, date pregnancy confirmed, estimated date of confinement, history of children born with congenital abnormalities, and history of spontaneous abortions.
2. Pregnancy Follow-up
   - Date of delivery or termination, outcome of pregnancy (i.e., spontaneous abortion, therapeutic abortion, ectopic pregnancy, stillborn delivery, liveborn delivery) and the presence or absence of congenital abnormalities in the infant.
3. Abnormal Pregnancy Outcome-mother (To be completed in the event of a delivery of an infant with congenital abnormalities.)
   - Complications during pregnancy, labor and delivery; information regarding prenatal care, infections, illnesses, and medications taken during pregnancy; use of drugs, alcohol, or other conditions during the course of the pregnancy.
4. Abnormal Pregnancy Outcome-infant (To be completed in the event of a delivery of an infant with congenital abnormalities.)
   - The sex, weight, estimated gestational age, and description of abnormalities present in the infant.

17.6 DETAILED DESCRIPTION OF SAMPLE SIZE CALCULATIONS

Power calculations were performed through computer simulation of 10,000 data sets using the SAS system. Sample size was determined for the intent-to-treat analysis. The calculations assumed an annual sero-incidence of 0.34% and a rate of loss to follow-up of 5% every 6 months during the follow-up period, or a cumulative dropout rate of 30.2% over 3 years of follow-up plus the vaccination phase. The rate of 5% includes volunteers who remain available for follow-up but did not receive all vaccinations within the protocol-specified time periods. The same rate of 5% was assumed for both the intent-to-treat and per-protocol analyses, since it is difficult to project what part of the 5% will be from loss to follow-up and what part from non-compliance with the vaccination schedule. Therefore the rate of 5% is an upper bound on loss to follow-up for the intent-to-treat analysis since this analysis includes individuals who do not comply with the vaccination schedule. In addition, it is assumed that 5% will be lost to follow-up during the 24-week vaccination period prior to the beginning of follow-up. Length of follow-up after the end of the vaccination period was assumed to be 3 years. An exponential model was assumed for the time-to-infection data and a uniform model was assumed for

the time-to-dropout data. Vaccine efficacy was assumed to be 25% during the 6-month vaccination period and 50% thereafter.

For each of the 10,000 simulated datasets, a sample of specified size was generated with half belonging to the vaccine arm and half belonging to the placebo arm. For each individual, a time-to-infection variable was generated from an exponential distribution. An inverse transformation method was applied to a SAS uniform random variate. For individuals in the placebo arm, the exponential parameter was set equal to –log(1-IR) where IR is the annual placebo sero-incidence rate. For each individual in the study vaccine arm, the exponential parameter was set equal to that in the placebo arm, multiplied by a factor of (1-VE), where VE is the assumed vaccine efficacy. In the study vaccine arm, a piecewise relative hazard function was assumed in which VE was 25% over the 6-month vaccination period and increased to 50% during the follow-up period.

For each individual, a binary dropout indicator variable was generated using the SAS random binomial function 'ranbin' with probability equal to the cumulative dropout rate where a dropout rate of 5% per 6 months is assumed over the course of the study. For individuals who dropped out of the study, time to dropout was generated uniformly over the time period including 24 weeks of vaccination and 3 years of follow-up. If the time to dropout was less than the time to infection, the infection status of the individual was considered censored. In addition, if the time to infection was greater than the length of follow-up, then the data for that individual was considered censored.

For purposes of sample size calculation, the time to infection and time to dropout data were discretized to reflect the fact that infection and dropout status will be measured at 6-month intervals rather than in continuous time. For example, if an individual's time to infection was 1.73 years, a discretized time to infection variable was created and set equal to 2 years, since the infection will be detected at the first semi-annual clinic visit after the actual time of infection.

Vaccine efficacy (VE) was estimated in each simulated dataset using a discrete failure time regression model which specifies a complementary log-log model for the hazard probability at each potential failure time (corresponding to semi-annual clinic visits). The models were estimated using the SAS 'Genmod' procedure. Vaccine efficacy was estimated using the estimated odds ratio from the discrete failure time regression model [VE = 100 x (1-OR)]. Confidence intervals for vaccine efficacy were estimated and based on transformations of confidence interval bounds for the odds ratio parameter.

Power was investigated with one interim analysis built into the study design. One interim analysis was planned for 36 months after the beginning of the accrual period, when approximately 2/3 of the follow-up information is expected to be available. Test statistics were compared to the O'Brien Fleming type boundary values in a spending function, Lan-DeMets, approach obtained from a program called 'ld98.exe' available from the University of Wisconsin website (/www.medsch.wisc.edu/landemets/). O'Brien-Fleming boundary values were calculated for one interim analysis at 2/3 information time and at the end of the study period. Overall significance level was assumed to be 0.05. Power was defined as the frequency of rejecting the null hypotheses under the specified alternative value for vaccine efficacy. The null hypothesis was rejected if it was rejected at the interim analysis, or accepted at the interim analysis and then rejected at the final analysis.

In each simulated data set, a hypothesis test of vaccine efficacy was conducted twice, first with data available at the time of the interim analysis and second with data available at the end of the follow-up period. The null hypothesis for the interim analysis was specified as true vaccine efficacy of 30%. In each simulation, the lower limit of the two-sided 95% confidence interval was compared to the specified value of 30%. The lower limit of the confidence interval was computed using the O'Brien-Fleming spending function boundary for one interim analysis at 2/3 information time. The

null hypothesis for the final analysis was that true vaccine efficacy would be equal to 0%. The statistical test at the final analysis was a 2-sided 5% level test of the hypothesis that the risk of infection is the same at any given time in the two treatment groups. The test statistic was to be compared to the O'Brien-Fleming error spending function boundary values for a final analysis with one preceding interim analysis at 2/3 information time. The test statistic at the final analysis in these simulations was evaluated only if the null hypothesis at the interim analysis was accepted. Initial investigations of sample size and power were conducted assuming no interim analysis. The null hypothesis for the analysis in those simulations matches the hypothesis in the final analysis for the actual planned design, that is, that true vaccine efficacy would be equal to 0%. The statistical test in the simplified simulations was a conventional 2-sided 5% level test of the hypothesis that the risk of infection would be the same in the two treatment groups.

17.7 REPOSITORY MANAGEMENT

Background:
Subject specimens and data collected during the Phase III Trial of Aventis Pasteur Live Recombinant ALVAC-HIV(vCP1521) Priming With VaxGen gp120 B/E (AIDSVAX® B/E) Boosting in HIV-uninfected Thai Adults may be used in compliance with terms of the Consent Documents signed by the volunteers. Volunteers in this study consented to donate their blood that was left over after testing from this project for use only in tests which address additional questions within the objectives of this study and are approved by the appropriate ethical review committee.

Establishment and Purpose of the RV144 Sample Repository Appendix:
The purpose of this appendix is to describe the policy and procedures to be followed when using subject specimens and data collected in this study. Ownership/stewardship, storage, use, distribution and disposition of human specimens and associated data for research purposes must adhere to Thai, U.S. federal, and U.S. Department of Defense requirements for the protection of human subjects. The RV144 protocol will remain open for 5 years with renewal subject to relevant IRB review and approval.

Collection/deposits:
The sample repository shall contain specimens collected under the RV144 vaccine trial. These specimens include plasma and peripheral blood mononuclear cells labeled the codes that can be linked to personal identifiers. The specimen archive database is maintained in the Department and does not contain identifiers. The linkage information is held in two places: 1) the linkage between subject study numbers and personal identifiers is contained in the study Vaccine Registry Database (VRD) as determined by Army regulation 70-25 and mandated in the protocol; 2) the data management center BIOPHICS. These linkages will be maintained for at least 75 years in order to contact volunteers in case of new safety data that could impact on their health. The study database is maintained because these volunteers might be of interest in studies that address additional questions within the objectives of this study and that are approved by the appropriate ethical review committee

Specimen and data storage:
The RV144 data and specimens will be stored at the HIV Vaccine Research Center for the Ministry of Public Health-Thai Vaccine Evaluation Group (MOPH-TAVEG)[i] investigators by the U.S. Army

component of the Armed Forces Institute for Medical Research Department of Retrovirology. The HIV Vaccines Product Management Office (HIVV PMO), USAMMDA, FT Detrick MD–shall have the responsibility to ensure proper housing, maintenance, and compliance with regulations governing storage and release of material, including maintenance of regulatory documents, specimen and data release approvals, and shipping logs. Research samples will be stored at the repository until the material has been exhausted, the repository is discontinued or the study is closed.

The HIVV PMO is responsible for provision of physical and network security, and continuous freezer monitoring. The U.S. Federal government provides the resources to maintain the repository, and repository management is administered either through direct U.S. Government support or through a specified contracting vehicle (such as a cooperative agreement, grant, sub-award, or contract). The RV144 samples and data are owned, as specified in the Materials Transfer Agreement signed by the U.S. Army and the Ministry of Public Health, by the MOPH-TAVEG investigators and jointly guaranteed by The Ministry of Public Health, and the U.S. Army. Within the repository, one aliquot per volunteer is reserved for use by the Thai National Government. Data and specimen management must permit samples and data from volunteers who have withdrawn consent to be identified for appropriate action. In addition, although these specimens will be under the stewardship as above, their use and release are governed by the procedures below.

Sample Storage:
Specimens will be cryopreserved in Thailand at a facility that will be equipped with restricted access, backup electricity generator, manual and electronic freezer monitoring, and staff are available for response at all times. Detailed records of temperature storage, equipment maintenance, transfer and chain of custody for all samples will be maintained on site. Electronic storage and backup of specimen data will be managed using systems developed and maintained by the Department without use of third party vendors for data storage.

Sample Destruction:
Specimens may be stored for the duration of the approved protocol. All relevant IRBs must specifically provide their consent for sample destruction, otherwise the reposed samples will be maintained, but no samples will be released, and all work on samples must be suspended, unless funds sufficient for maintenance can no longer be provided.
Access to specimens:
Investigators who wish to access RV144 data or samples must follow the processes contained in this appendix. Release of data or specimens will not occur until all of the following steps have been satisfied. It is expected that multiple requests will seldom be simultaneously received; in which case, the scientific processes below will include prioritization on the basis of scientific merit.

Step 1. The Principal Investigator/Sponsor: will administratively review each study proposal to assess whether the study addresses the objectives of RV144 as specified in the Voluntary Consent Form Version 4.7, 1 December 2005 and it is of sufficient importance to warrant use of study specimens and or data.

Step 2. Scientific review process (Membership Charter): An RV144 Scientific Review Committee (SRC) consisting of no fewer than 3 Subject Matter Expert scientists (including at least one Thai) not directly involved with the Study research will be appointed by the RV144 Steering Committee chaired

by the Sponsor. An SOP or charter for the SRC, to include member qualifications, quorum requirements, review/approval processes will be maintained at the Repository and will be available for review. The SRC will review a 1-2 page concept sheet provided by the research investigator(s). At a minimum, the concept sheet must include: research investigators' names and affiliations, scientific rationale, hypothesis to be tested, material and data requested, methods, source and availability of funding, and timeline for completion of work. Use of repository specimens and/or data requires completion of a scientific merit review by the RV144 Scientific Review Committee. The scientific review committee will provide approval, disapproval, or request clarifying revisions after assessing scientific validity of the hypothesis, appropriateness of the methods and specimens and data requested.

Research Ethics Committee/ Institutional Review Board Determination
Each project must have either an EC/IRB approval for research where identified data and/or specimens are proposed to be used or a "research not involving human subjects" determination for studies using specimens and or data that are coded or de-identified. It is required that the research investigator provide the IRB determination to the Principal Investigator and the Product Manager or designee. A listing of all studies that used RV144 specimens and/or data (identified, coded or deidentified) must be included in the annual continuing review report and will be retained at the Repository.

Agreements:
A written agreement governing the transfer and use samples and/or data for publication and presentations, which contains RV144 MOPH-TAVEG material transfer agreement (MTA) as an Appendix, will be in place prior to sample shipment.

Audit:
Any of the regulatory bodies overseeing this research may schedule an audit of the agreements overseeing the management and transfer of samples.

Availability of Specimen Determination:
The HIVV PMO will compile a sample set considering both the concept sheet and availability of specimen for particular volunteers and timepoints. In the case that the final aliquot for a particular volunteer-timepoint pair would be exhausted by the proposed project, the HIVV PMO will collaborate with the Scientific Review board, the PI, and the investigators to reach consensus on last-sample use or to identify other volunteer-timepoint pairs that could satisfy the requirements of the proposed research without consuming the final material. A sample set will be produced indicating specific samples and data to be released. The HIVV PMO will ensure that all of the above requirements are met before forwarding the sample set for final approval by the principal investigator.

Principal Investigator Approval:
Final release authority rests with the RV144 principal investigator or designee on behalf of the MOPH and MOPH-TAVEG, who will provide explicit approval for sample set release.

Distribution/Use:
After approval, release and distribution of approved sample sets will be the responsibility of the Chief of the Specimen Processing Laboratory in the Department of Retrovirology, who serves as the

Repository Laboratory Manager. The Department will maintain a tracking database that will characterize every specimen according to volunteer study number, timepoint, specimen volume, and for samples sent, recipient, and specimen type. In some cases, research proposals may require data that is not contained in the specimen repository database. In this case, distribution of data will be managed by the contract statisticians agreed to by the PI and Product Manager. In all cases, only limited datasets necessary for proposed analyses will be provided.

Restrictions on Sample and Data Usage at the Investigative Site:
Samples must be used or sent back to the repository, and may not be shared with third parties apart from separate approval as above. Study-associated and laboratory-generated data may be stored by research investigators indefinitely. The study data will be provided to the RV144 investigators for storage. In the unlikely event that something of health value is discovered through testing of samples, the investigator will notify the Principal Investigator. Notification of patients will only occur following consultation with participating institutions' EC/IRBs, specified in RV144 Section 2.1 relevant IRBs.

Investigators will be responsible for:
Submission of the concept sheet,
Obtaining appropriate scientific and IRB approval prior to initiation of repository research activities, to include shipment,
Ensuring that all research studies that utilize repository specimens and data have obtained separate IRB approval or determination for their proposed use of the repository,
Ensuring that processes are in place for the return of leftover specimens,
Ensuring that a relevant agreement or (such as a contract, Material Transfer Agreement, or Data Use Agreement) when applicable, has been executed prior to the transfer of research materials,
Having current CITI or equivalent training for themselves and staff members,
Payment for all costs associated with packaging and shipment of samples and/or data, and return of data and unused samples.

IRB Review for the Repository Appendix to RV144:
Approval of this Appendix by participating institutions' EC/ IRB, specified in RV144 Section 2.1will trigger constitution of the processes specified herein. Projects executed under this Appendix will be summarized on an annual basis with a report to participating institution's EC/ IRB inclusive of: the project title, the research investigators and affiliations, assays or analyses performed, samples or data sent, disposition of the data and/or samples, publications arising from this work, and any intellectual property arising from this work.
A summary of the collaborator IRB determinations will be stored at the Repository and available for audit by regulatory authorities.

Genetic testing:
Host immunogenetics may be performed in sub-studies, but the precise targeted regions must be specified in the concept sheet to avoid unnecessary assessment of genetic signatures that could affect a subject's employment, insurance coverage, etc. In the unlikely event that information of health value is discovered through testing of samples, the research investigator(s) will notify the Principal Investigator. Notification of patients will only occur following consultation with participating institutions' EC/IRBs, specified in RV144 Section 2.1.

Intellectual Property:
Any intellectual property or any other benefits must be managed according to international standards and relevant agreement(s) – e.g., data or materials transfer agreements, contracts. All such agreements must include the following statement: "Ownership of modification and direct/indirect derivatives of materials, and income arising from the commercializing the direct/indirect derivatives of the materials will be negotiated in good faith by the parties hereto depending upon (a) their relative contribution to the creation of said modifications and derivatives, and (b) applicable laws and regulations relating to inventorship."

---

[i] MOPH-TAVEG Collaborating Institutions: Ministry of Public Health (MOPH), Thailand: Vaccine Trials Centre, Faculty of Tropical Medicine, Mahidol University, Thailand; Data Management Unit (Biophics), Faculty of Tropical Medicine, Mahidol University, Thailand; Siriraj Hospital, Mahidol University, Thailand; RIHES U.S. Military HIV Research Program, U.S. Army Medical Component, AFRIMS, Thailand; Royal Thai Army, Thai Component, Armed Forces Research Institute of Medical Sciences (AFRIMS), Thailand; Research Institute for Health Sciences, Chiang Mai University.
